# Chromosome-level genome assembly of *Pinus massoniana* provides insights into conifer adaptive evolution

**DOI:** 10.1093/gigascience/giaf056

**Published:** 2025-05-30

**Authors:** Hu Chen, Xinghu Qin, Yinghao Chen, Haoyu Zhang, Yuanheng Feng, Jianhui Tan, Xinhua Chen, La Hu, Junkang Xie, Jianbo Xie, Zhangqi Yang

**Affiliations:** Guangxi Forestry Research Institute, Guangxi 530002, China; Key Laboratory of National Forestry and Grassland Administration on Cultivation of Fast-Growing Timber in Central South China, Guangxi 530002, China; Guangxi Laboratory of Forestry, Guangxi 530002, China; Guangxi Key Laboratory of Superior Timber Trees Resource Cultivation, Guangxi 530002, China; State Key Laboratory of Tree Genetics and Breeding, College of Biological Sciences and Technology, Beijing Forestry University, Beijing 100083, China; National Engineering Research Center of Tree Breeding and Ecological Restoration, College of Biological Sciences and Technology, Beijing Forestry University, Beijing 100083, China; School of Ecology and Nature Conservation, Beijing Forestry University & The Capital Biodiversity Conservation Institute, Beijing 100083, China; China (BJFU) -UK (St Andrews) International Joint Machine Learning Laboratory for Biodiversity Research, Beijing Forestry University, Beijing 100083, China; Guangxi Forestry Research Institute, Guangxi 530002, China; Key Laboratory of National Forestry and Grassland Administration on Cultivation of Fast-Growing Timber in Central South China, Guangxi 530002, China; Guangxi Laboratory of Forestry, Guangxi 530002, China; Guangxi Key Laboratory of Superior Timber Trees Resource Cultivation, Guangxi 530002, China; State Key Laboratory of Tree Genetics and Breeding, College of Biological Sciences and Technology, Beijing Forestry University, Beijing 100083, China; National Engineering Research Center of Tree Breeding and Ecological Restoration, College of Biological Sciences and Technology, Beijing Forestry University, Beijing 100083, China; The Tree and Ornamental Plant Breeding and Biotechnology Laboratory of National Forestry and Grassland Administration, Beijing Forestry University, Beijing 100083, China; Guangxi Forestry Research Institute, Guangxi 530002, China; Key Laboratory of National Forestry and Grassland Administration on Cultivation of Fast-Growing Timber in Central South China, Guangxi 530002, China; Guangxi Laboratory of Forestry, Guangxi 530002, China; Guangxi Key Laboratory of Superior Timber Trees Resource Cultivation, Guangxi 530002, China; Guangxi Forestry Research Institute, Guangxi 530002, China; Key Laboratory of National Forestry and Grassland Administration on Cultivation of Fast-Growing Timber in Central South China, Guangxi 530002, China; Guangxi Laboratory of Forestry, Guangxi 530002, China; Guangxi Key Laboratory of Superior Timber Trees Resource Cultivation, Guangxi 530002, China; Guangxi Forestry Research Institute, Guangxi 530002, China; Key Laboratory of National Forestry and Grassland Administration on Cultivation of Fast-Growing Timber in Central South China, Guangxi 530002, China; Guangxi Laboratory of Forestry, Guangxi 530002, China; Guangxi Key Laboratory of Superior Timber Trees Resource Cultivation, Guangxi 530002, China; Guangxi Forestry Research Institute, Guangxi 530002, China; Key Laboratory of National Forestry and Grassland Administration on Cultivation of Fast-Growing Timber in Central South China, Guangxi 530002, China; Guangxi Laboratory of Forestry, Guangxi 530002, China; Guangxi Key Laboratory of Superior Timber Trees Resource Cultivation, Guangxi 530002, China; Guangxi Forestry Research Institute, Guangxi 530002, China; Key Laboratory of National Forestry and Grassland Administration on Cultivation of Fast-Growing Timber in Central South China, Guangxi 530002, China; Guangxi Laboratory of Forestry, Guangxi 530002, China; Guangxi Key Laboratory of Superior Timber Trees Resource Cultivation, Guangxi 530002, China; State Key Laboratory of Tree Genetics and Breeding, College of Biological Sciences and Technology, Beijing Forestry University, Beijing 100083, China; National Engineering Research Center of Tree Breeding and Ecological Restoration, College of Biological Sciences and Technology, Beijing Forestry University, Beijing 100083, China; The Tree and Ornamental Plant Breeding and Biotechnology Laboratory of National Forestry and Grassland Administration, Beijing Forestry University, Beijing 100083, China; Guangxi Forestry Research Institute, Guangxi 530002, China; Key Laboratory of National Forestry and Grassland Administration on Cultivation of Fast-Growing Timber in Central South China, Guangxi 530002, China; Guangxi Laboratory of Forestry, Guangxi 530002, China; Guangxi Key Laboratory of Superior Timber Trees Resource Cultivation, Guangxi 530002, China

**Keywords:** *Pinus massoniana*, conifer evolution, genomic assembly, repeat sequences, gene family expansion, oleoresin biosynthesis, population genetics

## Abstract

*Pinus massoniana*, a conifer of significant economic and ecological value in China, is renowned for its wide adaptability and oleoresin production. We sequenced and assembled the chromosomal-level *P. massoniana* genome, revealing 80,366 protein-coding genes and significant gene family expansions associated with stress response and plant–pathogen interactions. Long-intron genes, which are predominantly presented in low-copy gene families, are strongly linked to the recent long terminal repeat burst in the *Pinus* genome. By reanalyzing population transcriptomic data, we identified genetic markers linked to oleoresin synthesis, including those within the *CYP450* and *TPS* gene families. The results suggest that the genes of the resin terpene biosynthesis pathway can be activated in several cell types, and the oleoresin yield may depend on the rate-limiting enzymes. Using a multiomics algorithm, we identified several regulatory factors, including PmMYB4 and PmbZIP2, that interact with *TPS* and *CYP450* genes, potentially playing a role in oleoresin production. This was further validated through molecular genetics analyses. We observed signatures of adaptive evolution in dispersed duplicates and horizontal gene transfer events that have contributed to the species adaptation. This study provides insights for further research into the evolutionary biology of conifers and lays the groundwork for genomic-assisted breeding and sustainable management of Masson pine.

## Introduction

Conifers, an ancient lineage of seed plants, play a crucial role in terrestrial ecosystems globally. Among them, *Pinus massoniana* (NCBI:txid88730) stands out as a dominant species in southern China, valued for its timber, pulpwood, and especially its rich oleoresin production [1], which significantly contributes to the national economy. Oleoresin not only contributes to the national economy but also serves critical ecological functions, acting as a defensive mechanism against pests and pathogens [2]. Given its ecological and economic significance, understanding the genetic underpinnings of *P. massoniana*’s adaptation to diverse environments becomes essential, particularly in relation to stress responses and oleoresin biosynthesis.

Recent strides in conifer genomics have shed light on the evolutionary adaptations of these species, unraveling unique characteristics and mechanisms that set them apart from other plant lineages [3–6]. However, existing studies predominantly focus on a limited array of conifer species such as *Picea abies, Pinus taeda*, and *Picea glauca* [7–9], which have provided valuable insights but also face several limitations. These genomes are typically large, complex, and rich in repetitive sequences, such as transposable elements (TEs), which complicate genome assembly and annotation [9]. Early sequencing efforts, often relying on short-read technologies, resulted in fragmented and incomplete genomes, requiring more advanced methods like long-read sequencing and Hi-C for improved assembly. For example, the *P. abies* genome was initially assembled using short-read sequencing, resulting in a fragmented assembly with large gaps [3]. Even with advances in sequencing technologies such as long-read sequencing (e.g., PacBio), gaps remain in functional annotation, with limited studies on specific traits like oleoresin biosynthesis or complex metabolic pathways. Moreover, many conifer genome studies, such as those on *P. taeda*, have focused primarily on general genomic features or stress-resistance traits like cold tolerance [10], leaving other critical traits, such as the biosynthesis of secondary metabolites (e.g., oleoresin), underexplored. In contrast, *P. massoniana*’s broad ecological range and oleoresin biosynthesis make it an ideal model for studying both evolutionary biology and functional genomics, particularly for understanding stress resistance and metabolic pathways unique to pine species.

*P. massoniana* has developed several adaptive strategies to thrive in diverse and challenging environments. Under low light and drought conditions, it modulates the production of secondary metabolites, such as flavonoids and terpenoids, which play crucial roles in defense mechanisms and stress tolerance [11]. Additionally, *P. massoniana* forms symbiotic relationships with ectomycorrhizal fungi like *Suillus luteus*, enhancing its nutrient absorption and resistance to soil-borne pathogens [12]. These adaptations collectively enable *P. massoniana* to withstand various environmental stresses, contributing to its ecological success and economic importance in subtropical regions.

The exceptional ability of *Pinus* spp., including *P. massoniana*, to synthesize oleoresin—a complex blend of turpentine and rosin crucial for defense against biotic and abiotic stresses—sets them apart [13]. This oleoresin not only ensures the tree’s survival but also holds substantial value for various industries, serving as a raw material for chemical and food sectors and a key precursor in biofuel production [14, 15]. Notably, *P. massoniana* accounts for 70% of China’s total oleoresin yield, underscoring its significance to the national economy [1]. The evolutionary origins of this biosynthetic machinery in conifers, a trait uncommon in most flowering plants, pose another intriguing question that our study aims to address. The sequencing of the *P. massoniana* genome represents a significant advancement in conifer biology. This chromosomal-level assembly provides crucial insights into the molecular mechanisms driving both resin biosynthesis and defense responses within the *Pinus* genus. By understanding the genetic pathways behind resin production and how conifers defend against pathogens and environmental stress, this research opens new avenues for improving conifer resilience and optimizing the production of resin-based compounds. Although the large genome size in conifers presents challenges for genome-wide analyses and sequencing efforts, the sequencing and analysis of these large genomes provide valuable insights into their evolution, adaptation, and the unique features that allow them to dominate various ecosystems around the world. Additionally, the study of the *P. massoniana* genome can offer new avenues for forestry and breeding due to their economic and ecological importance.

In this study, we present the first chromosome-level assembly of the *P. massoniana* genome, leveraging the power of next-generation sequencing technologies and advanced bioinformatics tools. We also performed genomic analyses based on genomic sequences, large-scale RNA sequencing (RNA-seq) data of 156 biological samples, and 204 transcriptomic data of wild accessions. Our analysis delves into the structural composition of the genome, the expansion of gene families, the evolution of conifer species, the key genes associated with adaptative traits, and the key factors involved in regulating resin terpene biosynthesis. The results provide insight into the genomic features and molecular mechanisms related to the resin terpene biosynthesis mechanism of *P. massoniana*. Our study paves the way for future studies on the evolutionary biology of conifers and has practical implications for the sustainable management and utilization of these ecologically and economically important species.

## Materials and Methods

### Plant materials

**Figure 1:**
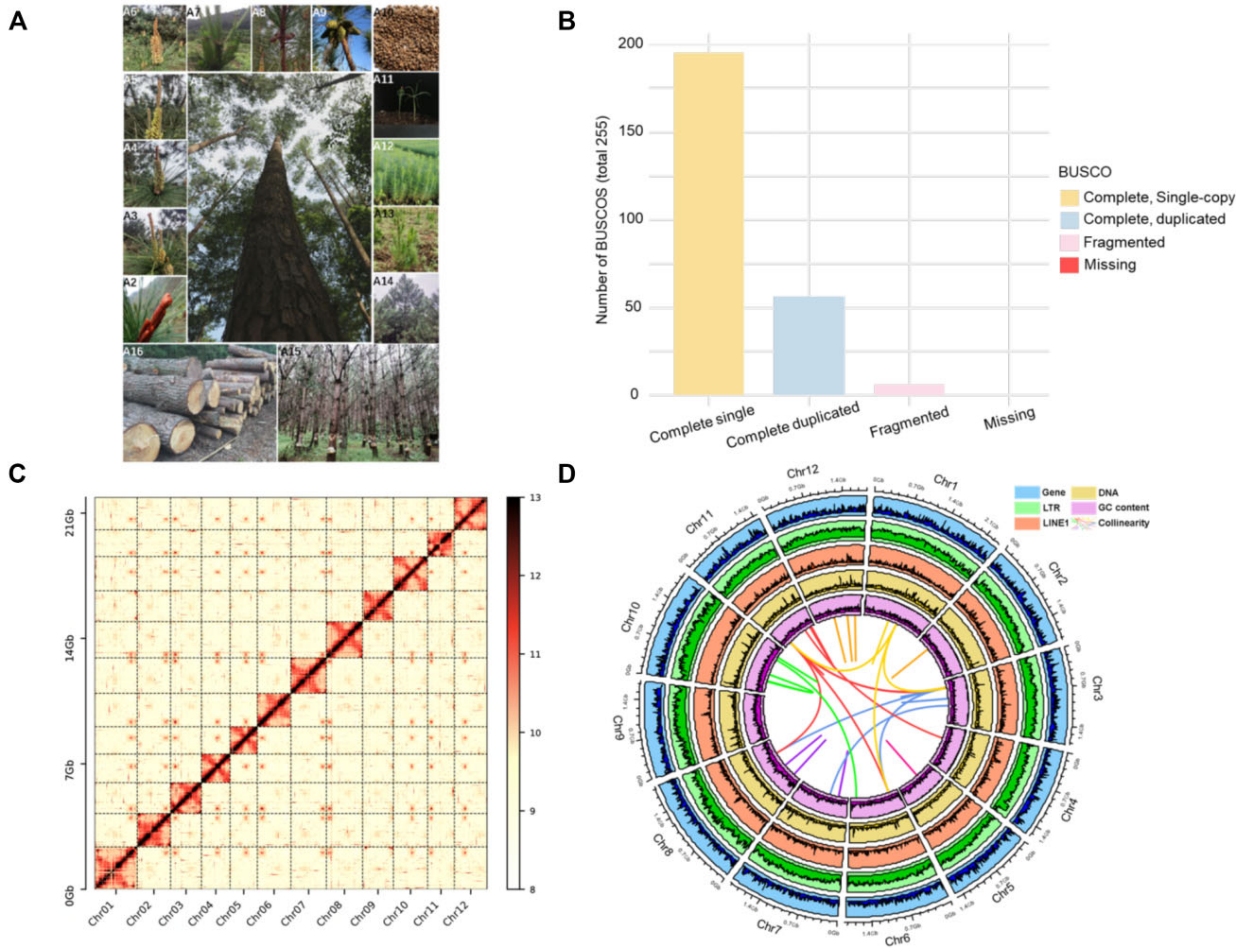
Genome assembly and features of *P. massoniana*. Morphology of *P. massoniana* (A1), *P. massoniana* cones at different developmental stages (A2–A9), different growth stages from seed to mature tree (A10–A14), resin tapping (A15), and timber (A16). (B) Genome quality assessment results by using BUSCO for *P. massoniana*. (C) Hi-C contact matrices of the 12 pseudomolecules of the final assembly. (D) Distribution of *P. massoniana* genomic features. The tracks from outer to inner circles represent different genomic features as indicated.

For our genomic study, we procured samples from a single, 6-year-old *P. massoniana* tree of the Songyun variety (Fig. 1A). This 6-year-old *P. massoniana* tree was selected for its representation of the mature growth phase when oleoresin production peaks, ensuring a robust dataset for investigating stress responses and metabolic activities relevant to oleoresin biosynthesis. This variety has undergone official audit by the Office for the Protection of New Varieties of Plants under the State Forestry Administration of the People’s Republic of China. The tree is situated in Nanning, China, with precise geographic coordinates of 23°10′N latitude and 107°59′E longitude. To obtain a representative sample, we collected approximately 50 grams of the current year’s mature needles from the midsections of the tree’s cardinal aspects—north, south, east, and west—and pooled them to ensure a uniform sample for analysis (Fig. 1A).

### DNA extraction

The genomic DNA (gDNA) was isolated from the pooled needle samples using the DNeasy Plant Mini Kit from QIAGEN, following the provider’s guidelines optimized for library construction and sequencing. The yield and purity of the extracted gDNA were evaluated using a Nanodrop One spectrophotometer (NanoDrop Technologies) and further confirmed by assessing the integrity with an Agilent 4200 Bioanalyzer (Agilent Technologies).

### Genome survey and genome size estimation

#### Genome survey

To estimate the genome size, heterozygosity, and repeat content, we used Jellyfish (RRID:SCR_005491) v2.1.4 [16] with the parameters of “-t 10 -C -m 41 -s 22G” to generate a 41 *k*-mer frequency distribution. Genome size (G) was estimated by G = *k*num/*k*depth, where the *k*num represents the total number of *k*-mers, and the *k*depth denotes the *k*-mer depth of the peak frequency of the *k-*mer distribution. Depth of *k*-mer = 1 is considered an error, and this error rate was used to calculate and correct the genome size. Then, the genome size, heterozygosity, and repeat content were estimated by the genomeScope [17] from the short sequencing data using Jellyfish v2.1.4 with the parameters of “-t 10 -C -m 41 -s 22G.” We also estimated the genome size by using the flow cytometry experiment, and nuclei were released by chopping the young needles and analyzed with the Beckman-Coulter Moflo XDP Cell Sorter (RRID:SCR_019665) according to the manufacturer’s instructions.

### Short-read sequencing

For the short-read sequencing, the qualified gDNA was randomly cut into fragments of approximately 350 bp in length. The size was further verified by using the Agilent 2100 Bioanalyzer (RRID:SCR_018043; Agilent Technologies), followed by end repair, polyadenylation, adapter ligation, target fragment selection, and PCR amplification using the Nextera XT DNA Library Prep Kit. Then, the Qubit 2.0 Fluorometer (Life Technologies) and Agilent 2100 (RRID:SCR_018043; Agilent Technologies) were used to check the preliminary quantitative and insert size of the library. Sequencing was performed on the BGI BGISEQ-500 platform (RRID:SCR_017979).

### Pacific Biosciences Technologies (PacBio) and Hi-C sequencing

#### PacBio library construction and sequencing

Pacific Biosciences Technologies (PacBio) libraries were constructed with a SMRTbell Template Prep Kit 1.0 (Pacific Biosciences) and the SMARTbell Damage Repair Kit (Pacific Biosciences). Sequencing was performed on the PacBio Sequel platform. In brief, the gDNA was sheared into fragments (∼20 kb) using a Covaris g-Tube (Covaris). The templates were size-selected using BluePippin (Sage Science) to enrich large DNA fragments (>15 kb), followed by primer annealing and the binding of SMRT bell templates to polymerases with the Sequel Binding Kit.

### Hi-C library construction and sequencing

For Hi-C sequencing, fresh needle samples were fixed in 1% formaldehyde to maintain the 3-dimensional structure of genome. The gDNA was extracted and digested with restriction endonuclease MboI. The sticky ends of the digested fragments were biotinylated, diluted, and ligated randomly. The ligated DNA was sheared into 300- to 600-bp fragments, blunt-end repaired, and purified. The libraries were sequenced on the BGI BGISEQ-500 platform, and 150-bp paired-end reads were generated.

### Genome assembly and chromosome anchoring

A hybrid strategy was used to assemble the genome sequence. The PacBio reads were used for initial contig assembly in Smartdenovo (RRID:SCR_017622) v. 2.3.1 software [18]. Next, the assembled contigs were polished 3 times using Nextpolish (RRID:SCR_025232) v. 1.3.1 software [19] based on the short reads. Subsequently, Hi-C sequencing data were used to anchor the draft genome with Juicer (RRID:SCR_017226) v. 1.6. To estimate genome size and heterozygosity, Jellyfish v. 2.1.4 [16] and Genomescope (RRID:SCR_017014) [17] software were used (*k*-mer = 41).

### Genome evaluation

We used short genomic sequencing data and the Isoform-sequencing (Iso-seq) full-length transcripts to evaluate the quality of assembly. The quality-controlled short genomic reads were mapped to the genome assembly using BWA-MEM (RRID:SCR_010910) [20], and information on the mapping ratio was collected. Evaluation by Iso-seq was performed in 2 steps. First, Iso-seq data were assembled into high-quality, full-length transcripts using SMRT-Analysis (RRID:SCR_002942) v.2.3. These full-length transcripts were then aligned to the genome using BLAT (RRID:SCR_011919) [21] to evaluate the structural accuracy of the assembly. In addition, the BUSCO (RRID:SCR_015008) (v4.1.4) [22] with embryophyta_odb10 and eukaryota_odb10 database was used to check the assembly quality and the gene annotation with genome and protein modes, respectively.

### Gene prediction

The hybrid approaches were used to predict protein-coding genes: homology-based search, *de novo* gene prediction, and RNA sequencing–aided annotation. (i) The assembled genome sequence was used for homology-based prediction using GeMoMa (RRID:SCR_017646) v1.8 [23] with default parameter based on 9 homologous species (*Amborell trichopoda, Oryza sativa, Pinus tabuliformis, Pseudotsuga menziesii, Arabidopsis thaliana, Cycas panzhihuaensis, Pinus lambertiana*, and *Ginkgo biloba*). (ii) SNAP (RRID:SCR_007936) [24] and AUGUSTUS (RRID:SCR_008417) v3.4.0 [25] were used for *ab initio* gene predictions. (iii) To improve gene prediction, we downloaded all the public available transcriptome samples from the public database. Then, the next-generation sequencing (NGS) transcriptome data were further assembled by Trinity, and the assembled transcripts were further processed using Tansdecoder v5.5.0 to obtain putative protein sequences. We used GMAP (RRID:SCR_008992) v2018-05-30 [26] to align the reads to the assemble genome and then used Transdecoder v5.5.0 to predict Open Reading Frame (ORF) in the transcripts to define putative protein sequences for Iso-seq data. Finally, all acquired results were combined and revised using EVM (RRID:SCR_008992) v1.1 [27] and Maker (RRID:SCR_005309) v3.01.03 [28]. The completeness of the *P. massoniana* genome sequence was estimated using BUSCO v5.0 software [22].

### Gene family expansion and contraction

Based on the phylogenetic tree, gene family expansion and the contraction of orthologous gene families were inferred using CAFÉ (RRID:SCR_005983) v.4.2. A random birth and death process was used to model gene gain and loss along each lineage in the phylogenetic tree. To make inferences over a whole phylogeny, a probabilistic graphical model was used to estimate the probability of transitions in gene family size from parent to child nodes [29] in the phylogenetic trees of *P. massoniana* and 12 other *Pinus* species.

### Analysis of synteny between *P. massoniana* and *P. tabuliformis*

We used JCVI (RRID:SCR_017650) v. 1.1.14 and MCscan [30] to identify syntenic gene pairs and blocks between *P. massoniana* and *P. tabuliformis*. The coding sequence (CDS) and genome annotation gff3 files of the 2 species were the input data, and we used “jcvi.compara.catalog ortholog” with default parameters to identify syntenic blocks for each pair. Next, “jcvi.compara.synteny screen” with the parameters –minspan = 30 –simple was used to filter syntenic blocks.

### Repeat annotation and long terminal repeat insertion time estimation

Repetitive and transposable elements in the *P. massoniana* genome were identified by RepeatMasker (RRID:SCR_012954) v.4.0.7 [31] using *de novo* libraries constructed by RepeatModeler (RRID:SCR_015027) [32]. Intact long terminal repeats (LTRs) were identified by LTR_FINDER (RRID:SCR_015247) v.1.0.6 [33] with default parameters. Then, all LTR pairs were aligned by using MUSCLE (RRID:SCR_011812), and the nucleotide distance (K) between them was estimated by using the distmat program in the EMBOSS (RRID:SCR_008493) package. The insertion time was calculated as T = K/2r, where the rate of nucleotide substitution (r) used for gymnosperm species was 2.2 × 10^–9^.

### Gene duplication analysis

Protein sequences were aligned all-versus-all using BLAST (RRID:SCR_004870) (v.2.2.28; -e 1e^−10^; -max_target_seqs 5). Next, the all-versus-all BLAST results and the gff3 files were used as input data for DupGen_finder [34] software; we used the default parameters to identify different modes of duplicated gene pairs. Syntenic regions with collinearity of paralog pairs were identified using MCScanX (RRID:SCR_022067) [35]. We analyzed the distribution of synonymous substitutions per site (Ks) for each paralog to evaluate recent whole-genome duplication (WGD) in *P. massoniana*. ParaAT v.2.0 [36] with the default parameters was used to construct multiple protein-coding DNA alignments. KaKs_Calculator (RRID:SCR_022068) v.2.0 [37] with the default parameters was used to calculate the Ks value for each paralog pair.

### RNA sequencing data analysis

The raw RNA-seq data of *P. massoniana* were quality-filtered using fastp (RRID:SCR_016962) software with default parameters [38]. Then, clean reads were mapped to *P. massoniana* using Hisat2 (RRID:SCR_015530) v2.0.9 [39] with the parameter –dta -x -p to generate read alignments for each sample. Gene expression levels were normalized using the number of transcripts per kilobase million reads by the StringTie software (RRID:SCR_016323) (v.1.3.5) with default settings [40].

### Functional enrichment analysis

To perform functional enrichment analysis, Gene Ontology (GO) terms and KEGG pathways were assigned to the genes using eggNOG-mapper (RRID:SCR_021165) v2 [41] with default parameters. R package clusterProfiler (RRID:SCR_016884) v3.0.4 [42] was used to perform GO and KEGG enrichment analysis of the expanded genes.

### Phylogenetic and domain analyses of terpene synthase and CYP450 proteins

MEME suite (RRID:SCR_001783) [43] with default parameters was employed to identify the conserved motifs of the terpene synthase (TPS) and CYP450 proteins. To construct the phylogenetic tree, multiple alignments were carried out using Muscle v3.6 [44], and iqtree2 (RRID:SCR_017254) [45] was then used to create maximum likelihood phylogenetic trees with parameters -T AUTO -st AA -bb 1000; bootstrap values were obtained by 1,000 bootstrap replicates. Phylogenetic trees were both visualized with iTOL (RRID:SCR_018174).

### Single nucleotide polymorphism calling

The transcriptome sequencing data [2] of 204 wild accessions from 10 main distribution regions were reanalyzed for single nucleotide polymorphism (SNP) calling. Filtered reads were mapped to the genome sequence, using SOAPaligner (RRID:SCR_005503) (SOAP2, version 2.20) with default options [46], which were used for SNP calling.

### Phylogenetic and population genetic analysis

To construct a phylogenetic tree, a dataset comprising 503,296 SNPs was employed to generate maximum likelihood (ML) trees using the IQ-TREE (RRID:SCR_017254) v2.2.2.3 software suite [47]. The optimal evolutionary model was selected based on the Bayesian information criterion (BIC). The robustness of the resultant ML trees was assessed utilizing the ultrafast bootstrap (UFboot) method, with 1,000 bootstrap replicates to estimate branch support. Visualization of the ML phylogenetic tree was facilitated through the Interactive Tree Of Life (iTOL) v4 online platform [48]. Principal component analysis (PCA) was conducted using PLINK (RRID:SCR_001757) v1.90p [49] and EIGENSOFT (RRID:SCR_004965) v6.1.4 [50] on the complete set of SNPs, applying filters for minor allele frequency (MAF) greater than 0.05 and allowing less than 10% missing data. The genetic structure of the population was delineated using ADMIXTURE (RRID:SCR_001263) software [51], with the number of presumed ancestral populations (K) ranging from 1 to 10. The most probable number of ancestral genetic clusters was inferred from the cross-validation error curve at the point of minimum K value. Diversity indices (π) and the population differentiation statistic (*F_ST_*) were computed using VCFtools (RRID:SCR_001235) v0.1.15 [52] on the filtered SNP dataset. For each subpopulation, these values were determined in a sliding window approach with a 20-kb window size and a 5-kb step increment.

### Genome-wide association study

In the genome-wide association study (GWAS), SNP loci with more than 10% missing data across accessions were excluded from analysis. Subsequently, SNPs with an MAF below 5% were subjected to GWAS. The mixed linear model (MLM) in TASSEL (RRID:SCR_012837) v5.2.51 [53] was applied to investigate the association between SNPs and oleoresin yield, adjusting for population structure (Q matrix) and kinship (K matrix) to control for confounding factors. The optimal Q matrix was ascertained using ADMIXTURE (RRID:SCR_001263) software, while the kinship matrix (K) was calculated using the KinshipPlugin within TASSEL. Associations were deemed significant at a *P* value threshold of ≤1.0E−5, which corrects for multiple testing and reduces the likelihood of type I errors.

### Transcription factors

The TFs were predicted using PlantRegMap according to the “Family assignment rules” of PlantTFDB (RRID:SCR_003362) [54]. The candidate TFs were further manually filtered by removing those without any conserved protein domains. For the phylogenetic tree construction, TFs of the same family were aligned by the MAFFT (RRID:SCR_011811) v7.520, with “–auto” option and “–maxiterate 1000,” and trimmed ambiguously aligned regions using trimAl (RRID:SCR_017334) v1.4 [55] with the “-automated1” option. Then, the ML tree was constructed by IQ-TREE v2.2.2.3 [47] with its best-fitting model of amino acid evolution and 1,000 ultrafast bootstrapping replicates [56].

### Horizontal transfer gene identification

To detect genes that may be acquired from distinct organisms, we employed a robust and conservative phylogeny-based approach, as described in a previous study with some modifications [57]. For each gene’s protein sequence, we used a 2-step workflow:

Step 1: We first performed the BLASTP (RRID:SCR_001010) in DIAMOND (RRID:SCR_009457) v2.1.6 [58] search against a custom database (reference protein sequences RefSeq and all proteins from PPGR) with an e-value cutoff of 10^−10^. HGTfinder v1 [57] was employed to parse the BLAST hits, based on their taxonomic information, into 3 different lineages (RECIPIENT: Streptophyta; GROUP: Viridiplantae; OUTGROUP: non-Viridiplantae). Five values were calculated: bbhO represents the BLAST bitscore of the best hit in the OUTGROUP lineage, bbhG represents the bitscore of the best hit in the GROUP lineage but not in the RECIPIENT lineage, and maxB represents the bitscore of the query to itself. The Alien Index (AI) was then calculated as (bbhO/maxB) − (bbhG/maxB), and outg_pct was determined as the percentage of species from the OUTGROUP lineage in the list of the top 1,500 hits that have different taxonomic species names. Genes that met the criteria of having an AI value greater than 0 and an outg_pct higher than 80% were considered highly credible horizontal gene transfer (HGT) genes.
Step 2: We retrieved the 1,500 most similar homologs from the Refseq database (as mentioned above). These homologs were then aligned using MAFFT (RRID:SCR_011811) v7.520 [59], with the “auto” option. Ambiguously aligned regions were trimmed using trimAl (RRID:SCR_017334) v1.4 [55] with the “automated1” option. The resulting alignments were used to infer the ML tree using IQ-TREE v2.2.2.3 [47]. The best-fitting model of amino acid evolution was employed, and 1,000 ultrafast bootstrapping replicates were performed. To root each ML tree, we utilized the ape and phangorn R packages [60, 61]. The rooted trees were then manually inspected.

### Identification of stress-resistance genes

BLAST (RRID:SCR_004870; v.2.2.28; -e 1e^−10^) was used to search for homologs in *P. massoniana* using the amino acid sequences of WRKY and AP2 in *A. thaliana* [62, 63] as references. To annotate nucleotide‐binding site‐leucine‐rich repeats (NLRs) in *P. massoniana*, we used the NLR-ID pipeline [64], and amino acid sequences were aligned to the NB-ARC HMM [65] of the NB-ARC domain using hmmalign with the default parameters (HMMER [RRID:SCR_005305] v.3.0) [66].

### Terpenoid biosynthesis pathway

Sequences encoding key enzymes of the terpene biosynthesis pathway [67, 68] were used as references to identify candidate functional homologs in *P. massoniana* using BLASTP v.2.2.28 (-e 1e^−30^).

### RNA *in situ* hybridization

*In situ* hybridization with digoxigenin (DIG)–labeled probes was performed as described previously [69]. Stem apex, needle, and root of *P. massoniana* were fixed in FAA solution (3.7% formaldehyde, 5% acetic acid, and 5% ethanol). The fixed tissues were dehydrated in a graded ethanol series, embedded in paraffin using a modular automated tissue processor (Leica ASP200S), and sectioned using a sliding microtome (Leica). After dewaxing and rehydrating, the sections were reacted with proteinase K (Roche), washed in phosphate-buffered saline, and subjected to acetylation. The sections were next prehybridized in hybridization buffer for 1 hour and incubated with a DIG-labeled riboprobe (Shanghai Gefan Biotechnology Co., Ltd.) for 48 hours at 65°C. After hybridization, the sections were rinsed, and the peroxidase reaction was initiated by adding 0.05% 3,3-diaminobenzidine-4 HCl (DAB) and 0.003% H_2_O_2_.

### Genetic transformation and molecular verification of transgenic plants

The CDS of *PmPGK* was cloned into the pBI121-GFP vector, generating a 35S:*PmPGK*-GFP construct. The 35S:*PmPGK*-GFP construct was introduced into the poplar 84 K transformation as described previously [70]. The presence of transgenic lines was confirmed through PCR analysis, and the expression levels of *PGK* were quantified using quantitative reverse transcription PCR (RT-qPCR) with pine 18S rRNA as an endogenous control.

### Dual‐luciferase assay

The dual-luciferase (LUC) assay was performed as previously described [71]. The coding sequence of *PmMYB4* was inserted into the pGreen II 62-SK vector to create an effector construct, while the promoter sequence of *PmCYP450.15* (2,000 bp) was cloned into the pGreenII 0800-LUC vector to generate a reporter construct. These constructs were cotransfected into *Nicotiana benthamiana* leaves, and the plants were incubated for 48 to 60 hours [72]. The leaves were sprayed with D-fluorescein (1 mM) and imaged using an LB983 Night Owl II fluorescence imaging system (Berthold Technologies) to detect luminescence. The relative luminescence intensity was quantified using Image-Pro Plus (RRID:SCR_007369) 6.0 software (Media Cybernetics), with each experiment being conducted in triplicate, including 3 biological replicates and 4 technical replicates.

### Electrophoretic mobility shift assay

For the electrophoretic mobility shift (EMSA), the full-length *PmMYB4* was expressed in *Escherichia coli* strain Rosetta (DE3) using the pHMGWA expression vector. The His-tagged PmMYB4 protein was induced by 0.05 mM isopropyl-*β*-D-1-thiogalactopyranoside and purified using Ni-NTA Agarose according to the manufacturer’s instructions (Qiagen). Biotinylated probes containing the GCC-box or mutated elements were synthesized and used in EMSA with the Light Shift Chemiluminescent EMSA Kit (Pierce). The DNA protein-binding reactions were performed in a buffer containing 5 mM MgCl_2_, 50 mM KCl, 10 mM EDTA, 2.5% glycerol, 50 ng/µL Poly (dI‐dC), and 0.05% NP-40, followed by separation on a 6.5% nondenaturing polyacrylamide gel and transfer to a nylon membrane for chemiluminescence detection.

### Subcellular localization

The subcellular localization of PmMYB4 and PmbZIP2 was determined by cloning its CDS into the pBI121-GFP vector, resulting in a *35S:PmMYB4-GFP* and *35S:PmbZIP2-GFP* construct. This construct was transiently expressed in tobacco protoplasts using established methods [73]. The fluorescence from the transformed protoplasts was visualized using a Zeiss LSM710 confocal microscope after 12 to 16 hours. The cell membrane was stained with FM4-64 to provide a reference for subcellular localization, with fluorescence detected at excitation/emission maxima of 515/640 nm.

## Results

### Chromosome-scale sequencing and assembly of the *P. massoniana* genome

We employed a hybrid sequencing approach combining high-coverage short reads, Pacific Biosciences (PacBio) long reads, and Hi-C chromatin interaction data to generate a high-quality chromosome-level genome assembly of *P. massoniana*. To evaluate the accuracy and completeness of the genome assembly, we performed several quality control measures. First, we mapped the short reads back to the assembled genome, achieving a mapping rate of 99.2%, which indicates high accuracy in the assembly (Supplementary Fig. S3A). Second, we used BUSCO to assess the completeness of the assembly (Fig. 1B; Supplementary Fig. S3B). The total length of the assembled genome was approximately 21.91 Gb, comprising 49,639 contigs with an N50 contig length of 4 Mb (Supplementary Figs. S1 and S2). The contigs were further scaffolded using Hi-C data, anchoring them to 12 pseudochromosomes that represent the haploid genome, with the lengths of pseudochromosomes ranging from 1.49 to 2.37 Gb, accounting for 99.89% of the assembled sequences (Fig. 1C). The final assembly had an N50 scaffold length of 1.5 Gb, with 96.7% of the genome anchored to the chromosomes (Supplementary Table S1). The genome contained 95.3% of the conserved single-copy orthologs from the embryophyta dataset, confirming the high quality of the assembly (Supplementary Table S2).

### Repetitive sequences and transposable elements

The *P. massoniana* genome assembly harbored 81.2% (17.79 Gb) of the repetitive sequence (Fig. 1D; Supplementary Table S3), of which the LTR retrotransposons (LTR-RTs) and Long Interspersed Nuclear Element (LINE) represented 60.15% and 3.06% of the assembly, respectively. Notably, Gypsy LTR-RT elements and Copia LTR-RT elements accounted for 58.38% of the genome (Supplementary Table S3). The Gypsy LTR-RTs (43.59%) were disproportionately abundant in *P. massoniana* compared to other gymnosperms, a phenomenon potentially attributable to recent species-specific bursts in multiple subfamilies of LTR-RTs (Supplementary Fig. S5). The majority of LTR-RT expansions occurred within the last 5 to 30 million years (Supplementary Fig. S5), coinciding with the Miocene epoch (5.33–23.03 million years ago [MYA]), a period characterized by global cooling, leading up to the ice ages [74–76].

### Gene family evolution in *P. massoniana*

In total, we annotated 80,366 protein-coding genes and 132,148 transcripts based on extensive RNA-seq data from 156 biological samples representing various tissues and stress conditions (Supplementary Table S4). Of these, 89.23% aligned with entries in databases such as Swiss-Prot, KEGG, and Gene Ontology.

**Figure 2:**
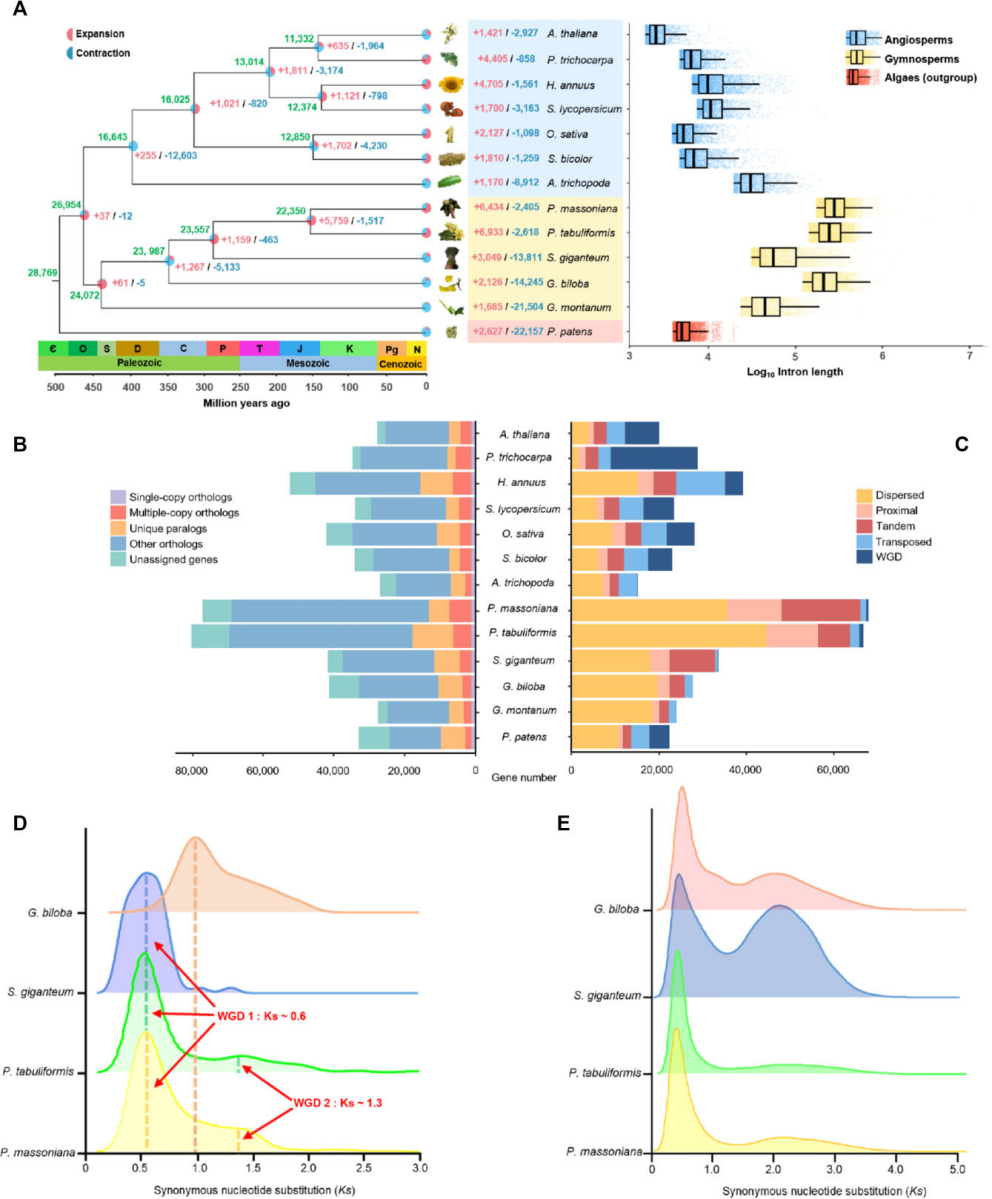
Gene family evolution in *P. massoniana*. (A) Gene family expansion and contraction across the 13 plant species. The numbers of gene families in last common ancestors are highlighted in green; the expansion and contraction of gene families in the subbranches are highlighted in red and blue, respectively. Number on the coordinate axis represents the divergence time of each branch. In the right panel, boxplots indicate the intron lengths of 13 species. (B) Barplots indicate the number of different gene categories. (C) The number of genes derived from different duplication event, including WGD, transposed, tandem, proximal and dispersed duplication. (D) The distribution of Ks values of the WGD gene pairs of *P. massoniana, P. tabuliformis, G. biloba*, and *S. giganteum*. (E) The distribution of Ks values of the dispersed gene pairs of *P. massoniana, P. tabuliformis, G. biloba*, and *S. giganteum*.

To explore the evolutionary trajectory of *P. massoniana*, we conducted a comprehensive phylogenetic analysis using 135 single-copy orthologous genes from 13 plant species, including 12 published genomes and *P. massoniana*. This analysis allowed us to identify and classify 30,558 gene families in the *P. massoniana* genome, comprising 69,152 genes. Of these gene families, 5,215 were found to be conserved across all 13 species, while 9,846 were unique to *P. massoniana*. Additionally, 73,608 genes were identified as having orthologs in the other 12 plant genomes (Fig. 2A, B).

Through molecular clock analysis, we estimated that the most recent common ancestor (MRCA) of the Coniferopsida lineage, which includes *P. massoniana*, contained approximately 23,557 gene families. Following the divergence of the Pinaceae and Cupressaceae lineages around 289 MYA, the Pinaceae family, including *P. massoniana*, underwent substantial gene family expansion, gaining 5,759 new gene families while losing 1,517 (Fig. 2A). In addition, 6,434 gene families have expanded in the *P. massoniana* lineage, while 2,405 gene families have contracted (Fig. 2A). Notably, many of the expanded gene families are associated with stress resistance and terpene biosynthesis, including members of the cytochrome P450 (CYP450) and TPS families, which play crucial roles in oleoresin production. On the other hand, the average lengths of coding sequences, exons, and introns were 1,007 bp, 1,005 bp, and 12,680.84 bp, respectively. Notably, *P. massoniana* exhibited the longest average intron length compared to other analyzed species (Fig. 2A; Supplementary Table S5).

Our analysis revealed 57,207 duplicated genes, primarily resulting from dispersed duplication (56%) and WGD events (0.79%) (Fig. 2C). Syntenic analysis with *P. tabuliformis* revealed 1,471 syntenic blocks, encompassing 25,936 anchor genes (Supplementary Table S6). The analysis indicated a low level of synteny, suggesting rapid chromosomal rearrangements within the Pinaceae family. Two distinct peaks in synonymous substitution divergence (Ks) were identified, suggesting that *P. massoniana* has experienced 2 WGD events approximately 320 MYA and 260 MYA (Fig. 2D).

### Functional enrichment of expanded gene families

Functional annotation of the expanded gene families in *P. massoniana* revealed a strong association with stress response mechanisms and plant–pathogen interactions. Pathways related to responses to xenobiotic stimuli, cellular responses to water deprivation, and terpene biosynthesis were significantly overrepresented among the expanded gene families (Supplementary Fig. S4; Supplementary Table S7). Several pathways related to stress responses, flavonoid biosynthesis, and long-term protection have expanded in *P. massoniana* (Supplementary Fig. S4; Supplementary Table S7). These results suggest that *P. massoniana* has evolved a robust defense strategy to cope with environmental stressors and pathogen challenges.

### Dispersed gene duplications and evolutionary dynamics

To examine the dynamics of gene duplication in *P. massoniana*, we analyzed the distribution of synonymous substitution rates (Ks) for dispersed gene duplicates across 4 gymnosperm genomes, including *P. massoniana, G. biloba*, and *Sequoiadendron giganteum* (Fig. 2E). The *P. massoniana* genome exhibited a continuous distribution of Ks values, suggesting that dispersed gene duplications have been an ongoing process in this species. In contrast, *G. biloba* and *S. giganteum* displayed 2 distinct Ks peaks, indicating episodic duplication events.

Interestingly, we observed a strong correlation between transposable element (TE) activity and dispersed gene duplication (DSD) events in the *P. massoniana* genome (Supplementary Fig. S5). The *Pinus* lineage showed a recent (<10 MYA) proliferation of LTR-RTs, which likely contributed to the expansion of dispersed gene duplicates. In contrast, the other gymnosperm species exhibited older LTR-RT bursts, suggesting differing evolutionary trajectories for TE activity and DSD events across gymnosperms.

### Intron length and gene expression

Consistent with previous studies, we found that gymnosperms, including *P. massoniana*, possess significantly longer introns compared to angiosperms (Fig. 2B). In *P. massoniana*, 18,536 introns exceeded 20 kb in length, with an average intron length of 11.56 kb. Using PacBio long-read sequencing, we validated the authenticity of these long introns, confirming that they are not assembly artifacts but genuine features of the genome (Supplementary Fig. S6).

Our analysis revealed a strong negative correlation between intron length and gene family size, particularly in low-copy-number genes (Supplementary Fig. S7). Low-copy-number genes tend to have longer introns and are more conserved across species, suggesting that intron retention may confer evolutionary advantages, such as enhancing gene regulation and transcriptional efficiency. We also observed that the genome size is positively correlated with intron length (Supplementary Table S8). Additionally, highly expressed genes in *P. massoniana* were more likely to have longer introns, a pattern consistent with observations in model plant species like *A. thaliana* and *O. sativa*. The average lengths of genes in gymnosperms were longer than in angiosperms, implying that genome composition is related to gene length. Also, the gene-coding sequences of gymnosperms and angiosperms were of constant average lengths despite the marked intergenic variation in exon sequence length (Supplementary Fig. S8).

### Stress resistance and horizontal gene transfer

In the genome of *P. massoniana*, we identified 550 NLR genes, with the majority (107 gene pairs) likely resulting from dispersed duplication and 65 pairs from tandem duplication (Supplementary Fig. S9). Our analysis of transcription correlation coefficients revealed that the correlations between gene pairs of genome-wide tandem duplicates (13,088 gene pairs) were significantly lower than WGD gene pairs (*P* < 0.001, permutation test), but correlations between gene pairs derived from tandem duplication were not significantly different from those of dispersed duplication events.

Gymnosperms had the smallest percentage of TFs among all genes (2.74%–3.93%; Supplementary Fig. S10). WRKY TFs, which are essential for plant stress tolerance and disease resistance [77], were found to be activated by various treatments and stresses. We detected 31 WRKY TFs, with 27 being expressed in response to a variety of stresses, including prolonged drought, aluminum (Al) stress, and methyl jasmonate (MeJA) treatment (Supplementary Figs. S9C, S11–S12). The expansion of cold-responsive *AP2/ERF* genes, specifically the III group of C-repeat binding factors (*CBFs*), is associated with plant adaptation to paleoenvironmental changes [78, 79]. In *P. massoniana*, we identified 24 *AP*2/*ERF* genes, and transcriptome analysis of 31 samples subjected to various stresses revealed that these genes were actively expressed, with half showing specific expression patterns (Supplementary Figs. S9C, S11–S12).

We identified 689 genes likely acquired through HGT, including 59 from fungi and 109 from bacteria (Supplementary Table S9). These horizontally acquired genes included several carbohydrate-active enzymes (CAZymes) involved in cell wall biosynthesis, as well as glycoside hydrolases (GH71 and GH3), which are associated with plant defense and stress responses. Notably, we detected the expansion of ATP-binding cassette (ABC) transporter proteins, which are known to contribute to stress tolerance and environmental adaptation in terrestrial plants. Additionally, several horizontally acquired phosphoglycerate kinase (PGK) genes were identified, with some showing evidence of involvement in salt stress resistance (Supplementary Fig. S13). These findings suggest that HGT has contributed to the adaptive potential of *P. massoniana* by introducing genes that enhance its ability to cope with environmental stressors.

### Resin terpene biosynthesis and gene expression patterns

**Figure 3:**
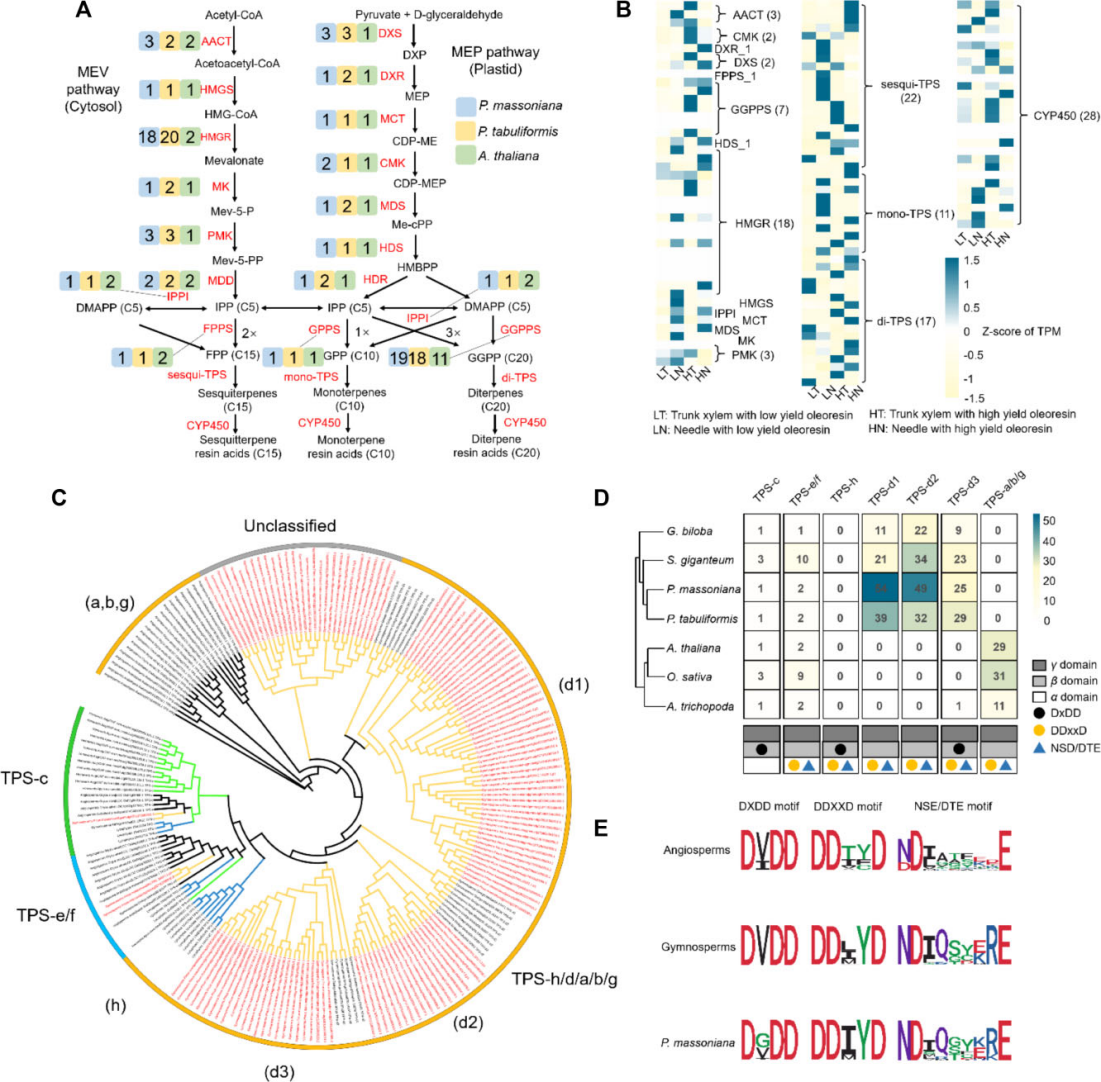
The resin terpene biosynthesis pathways and phylogenetics of the *TPS* family and conversed motifs in *P. massoniana*. (A) The resin terpene biosynthesis pathways in *P. massoniana*. (B) Different genes of key enzymes in terpenoid pathways are shown in heatmaps. LT represents the sample of trunk xylem with low yield oleoresin, LN represents the sample of the needle with low-yield oleoresin, HT represents the sample of trunk xylem with high-yield oleoresin, and HN represents the sample of the needle with high-yield oleoresin. (C) Phylogenetic ML tree of *TPS* genes of *P. massoniana*. (D) Number of homologous genes in each subfamily of *TPS* genes from 4 gymnosperms (*P. massoniana, P. tabuliformis, G. biloba, S. giganteum*) and 3 angiosperms (*A. thaliana, O. sativa, A. trichopoda*). The composition of domains (*α, β*, and *γ*) and conversed motifs (“DXDD,” “DDXXD,” and “NSE/DTE”) are noted for each subfamily. (E) The conversed motifs (“DXDD,” “DDXXD,” and “NSE/DTE”) in angiosperms, gymnosperms, and *P. massoniana*.

Resin terpene biosynthesis plays a critical role in the defense mechanisms of conifer species. In *P. massoniana*, we identified 219 candidate genes encoding enzymes involved in the 22 steps of resin terpene biosynthesis (Fig. 3). These include enzymes responsible for the biosynthesis of isopentenyl pyrophosphate (IPP), geranyl diphosphate (GPP), and the final terpenoid products.

Comparative transcriptomic analysis between high- and low-oleoresin-yield *P. massoniana* genotypes revealed higher expression levels of rate-limiting enzymes such as DXS and HMGR in high-yield genotypes (Fig. 3B). Furthermore, 5 GGPPS genes, involved in synthesizing the diterpene precursor, showed enhanced expression in high-oleoresin-yield genotypes. However, TPS genes, responsible for synthesizing the final terpenes, did not exhibit clear expression differences between genotypes, suggesting that regulation of terpene biosynthesis may occur at other enzymatic steps.

We further evaluated the expression levels of 10 key genes in stem, needle, and root using RNA *in situ* hybridization (Supplementary Table S10). It confirmed that key genes of the resin terpene biosynthesis pathway were expressed in several cell types, including the epidermis, sclerenchyma, cortex, and xylem resin cells (Supplementary Fig. S14). This indicates that oleoresin biosynthesis is a highly regulated process, with its yield potentially dependent on the activity of rate-limiting enzymes.

### Regulatory networks controlling oleoresin biosynthesis

**Figure 4:**
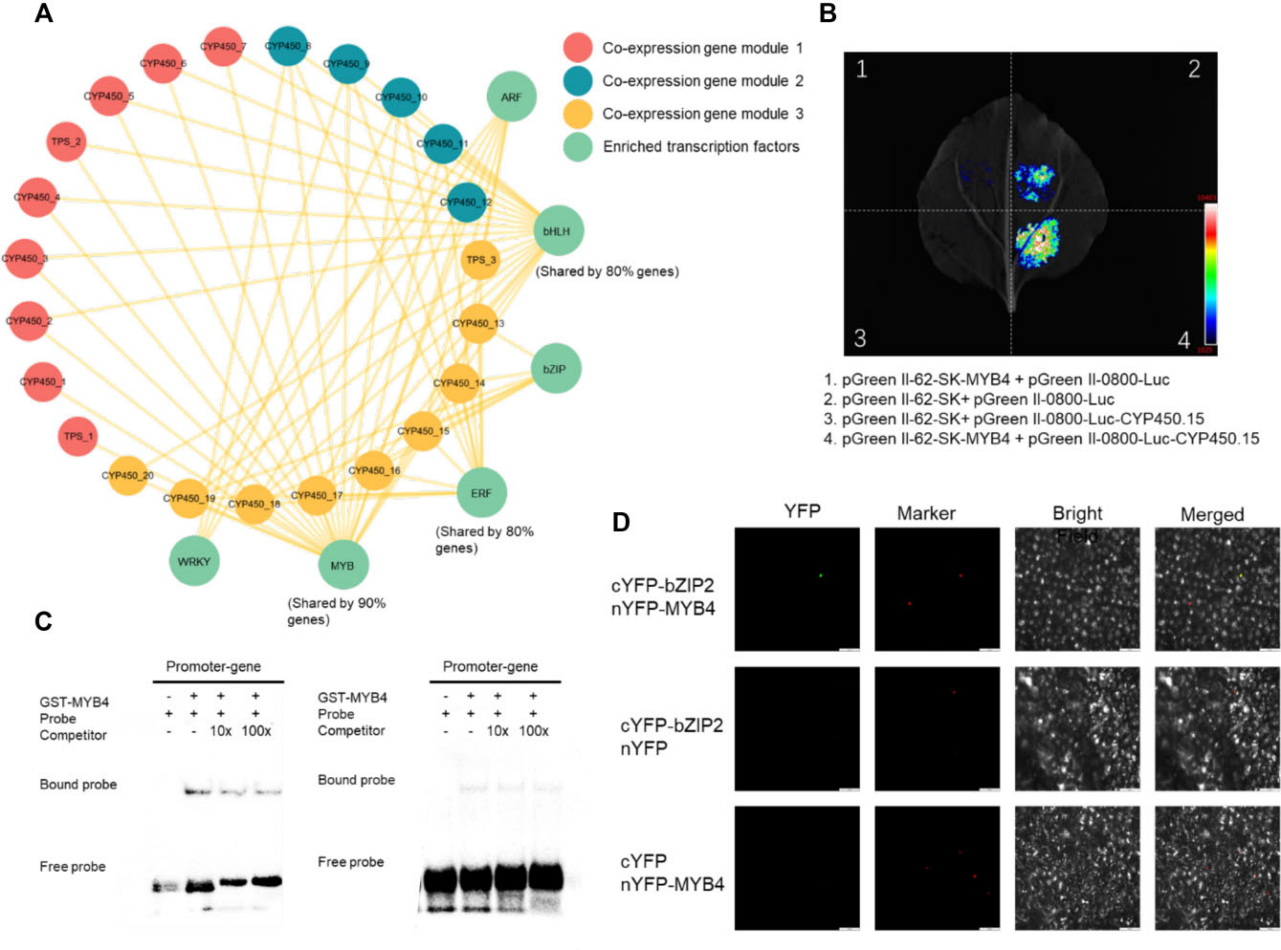
Enrichment analysis of the promoters of coexpression modules of *CYP450, TPS* genes and interactions between PmMYB4 and PmCYP450.15, PmbZIP2. (A) Coexpression modules of *CYP450* and *TPS* genes generated by WGCNA analysis. The transcription factors labeled specifically were shared by more than 80% of all genes. *TPS_1: gmmutg18241G000030.2; TPS_2: gmmutg17794G000150.1; TPS_3: gmmutg42131G000020.1; CYP450_1: gmmutg68017G000020.1; CYP450_2: gmmutg26354G000020.1; CYP450_3: gmmutg20443G000020.1; CYP450_4: gmmutg178448G000020.1; CYP450_5: STRG.13291.1.p1; CYP450_6: gmmutg133003G000010.1; CYP450_7: gmmutg388649G000010.2; CYP450_8: gmmutg17794G000030.1; CYP450_9: gmmutg1680G000040.1; CYP450_10: MSTRG.47921.2.p1; CYP450_11: gmmutg101230G000060.1; CYP450_12: gmmutg5617G000080.1; CYP450_13: STRG.13290.1.p1; CYP450_14: gmmutg6309G000040.2; CYP450_15: MSTRG.30814.1.p1; CYP450_16: STRG.25141.1.p1; CYP450_17: gmmutg11433G000020.1; CYP450_18: gmmutg1483G000030.1; CYP450_19: gmmutg269G000120.1; CYP450_20: gmmutg1771G000090.1; PmMYB4*: gmmutg10484G000010.1; *PmbZIP2*: gmmutg3867G000050.1. (B) Representative luciferase luminescence image of *N. benthamiana* leaves coinfiltrated with the agrobacterial strains containing PmCYP450.15pro‐Luc and PmMYB4‐62‐SK. Tobacco leaves injected with empty vector controls, pGreenII 0800‐LUC and pGreenII 62‐SK, were used as a negative control. (C) EMSA assays were applied to identify the interactions between *GSTMYB4* protein and the promoter gene. Here, 10 and 100 unlabeled probes and probe mutants were used in the competition experiment. (D) Subcellular localization of cYFP-bZIP4 and nYFP-MYB4 in transiently expressed tobacco leaves. Scale bar = 10 µm.

To further explore the regulation of oleoresin biosynthesis, we performed promoter analyses and coexpression studies focusing on SNP-associated key genes involved in terpene synthesis. These analyses revealed 3 major gene coexpression modules (coexpression modules 1–3), which included genes from the CYP450 and TPS families that are critical for oleoresin biosynthesis (Fig. 4).

Six upstream TFs were identified as potential regulators of these modules: ARF, bHLH, bZIP, ERF, MYB, and WRKY. Among these, MYB binding sites were present in 90% of the promoters of the genes in the coexpression modules, suggesting a central role for MYB in the regulation of oleoresin biosynthesis. Notably, the transcription factor PmMYB4 emerged as a key regulatory component, with strong evidence indicating its interaction with downstream CYP450 genes.

Experimental validation using LUC assays and EMSAs confirmed the direct interaction between PmMYB4 and the promoter regions of key CYP450 genes (Fig. 4). This direct regulation suggests that PmMYB4 plays a pivotal role in modulating the expression of genes involved in oleoresin synthesis. Additionally, PmMYB4 was found to colocalize with another transcription factor, PmbZIP2, to jointly regulate a subset of TPS and CYP450 genes, further indicating a complex regulatory network controlling oleoresin biosynthesis.

### The population genetic structure and the genetic basis of oleoresin yield

Reanalyzing 204 samples from various geographical locations [2] using PCA, STRUCTURE, and Phylogenetic ML tree revealed consistent 3 distinct genetic clusters, which aligned with the geographical distribution of *P. massoniana*, corresponding to the South, Southeast, and West China populations (Supplementary Fig. S15). These clusters were well defined and showed high correspondence with the 3 major geographical regions of *P. massoniana*, indicating a strong influence of geographic effect on the genetic structure of this species. A phylogenetic tree constructed for a subset of single-copy orthologous genes showed a clear partitioning of gene copies into the 3 identified genetic clusters, suggesting that these clusters have a historical and potentially adaptive significance. From here, the populations have expanded outward, primarily along 2 trajectories: one toward the south and the other toward the southeast (Supplementary Fig. S15).

The Transcriptome-Wide Association Study (TWAS) of oleoresin yield in *P. massoniana* identified 6,064 key genetic markers (SNPs) significantly associated with oleoresin synthesis and production (*P* ≤ 0.01). These SNPs comprise several functional protein families and motifs. Notably, kinesin family proteins, which are involved in intracellular transport, were highlighted by the presence of the PFAM domains Kinesin, suggesting a potential role in the cellular mechanisms underlying oleoresin production. The zinc phosphodiesterase ELAC protein, which contains Lactamase B 2 and Lactamase B 4 domains, potentially links RNA processing to oleoresin synthesis. The GTP diphosphokinase CRSH protein, localized in the chloroplast, which contains EF-hand 1, EF-hand 5, HD 4, and RelA SpoT domains, is crucial for energy metabolism and could be a key regulator in the biosynthesis of oleoresin. Notably, among the strongly associated SNPs, one set of important SNPs related to oleoresin biosynthesis includes the terpene synthase family (Supplementary Table S11). SNPs belonging to the terpene synthase family, marked by terpene synthesis and terpene synthesis C domains, are key enzymes in the biosynthesis of terpenes, which are major components of oleoresin. Additionally, the protein NRT1 PTR family, associated with transport and metabolism of nitrogenous compounds, could be indirectly linked to oleoresin yield. SNPs within the small heat shock protein (HSP20) family are known to protect cells against various stresses, potentially including those encountered during oleoresin synthesis. In addition, among the loci significantly associated with oleoresin yield in GWAS, 51 SNPs are annotated to the cytochrome P450 family and *TPS* genes (Supplementary Table S11). This superfamily of enzymes is known for their role in the metabolism of endogenous and exogenous substances, including the biosynthesis of oleoresin. There are also other SNPs showing a strong association with the oleoresin yield, such as proline-rich nuclear receptor coactivator motif, glycosyl hydrolase family 3 N terminal domain, ethylene-responsive transcription factor, protein NRT1 PTR family, N-oligosaccharyl transferase (OST) complex, and the calcium-dependent phosphotriesterase superfamily protein. They are all implicated in the regulation or biosynthesis of oleoresin. These findings enhance our understanding of the genetic underpinnings of oleoresin yield and provide a foundation for future genetic improvement strategies in Masson’s pine.

### The synthesis of oleoresin may be under the control of a coordinated regulatory system involving multiple TFs

SNP-based association analyses provide a suitable approach for identification of key genes and genetic regulatory networks; thus, it may be useful for exploring the regulation of genes in the resin terpene biosynthesis pathway. Here, we performed a multifaceted approach combining multiple full-length SNP-associated key genes and the coexpression network. As a result, we identified several key modules of *CYP450* and *TPS* genes associated with the biosynthesis of oleoresin that displayed significant coexpression patterns. For instance, key CYP450 genes include CYP720B4 and CYP720B6, while critical TPS genes include TPS1 and TPS6. These genes were found to be part of 3 major coexpression modules (coexpression gene modules 1–3), which in turn were linked to 6 upstream transcription factors (ARF, bHLH, bZIP, ERF, MYB, WRKY) that likely regulate their expression (Fig. 4). Notably, MYB binding site was shared by 90% of the promoters of these genes. Thus, the transcription factor PmMYB4 was identified as a key regulatory component in this network, with evidence suggesting its interaction with downstream *CYP450* genes.

Further molecular studies using LUC assays and EMSAs provided experimental evidence for the direct interaction between PmMYB4 and the promoter regions of *CYP450* genes, indicating a role for PmMYB4 in the transcriptional regulation of these genes (Fig. 4). Additionally, PmMYB4 may act in concert with PmbZIP2 to regulate a subset of downstream *TPS* and *CYP450* genes, with both factors showing colocalization at the molecular level.

The discovery of these regulatory interactions suggests that the synthesis of oleoresin in *P. massoniana* is under the control of a coordinated regulatory system involving multiple TFs. For instance, TFs such as WRKY and bZIP have been implicated in stress responses and may also play a role in the context of oleoresin biosynthesis. This coordinated regulation could be crucial for the plant’s ability to adapt to various environmental stresses and optimize oleoresin production.

## Discussion

Gymnosperms, a diverse and ancient lineage of plants that originated around 270 MYA, are renowned for their ecological importance and evolutionary distinctiveness [80]. Conifers, as a unique lineage of gymnosperms, have captured the attention of biologists with their remarkable diversity, ecological significance, and evolutionary history. In this study, we report the first chromosome-level genome assembly of *P. massoniana*, which provides a rich resource for understanding the genomic underpinnings of a species that is both ecologically significant and economically valuable and offers insight into the evolution of the gymnosperm genome. Our findings reveal a complex interplay between genomic architecture, gene family expansions, and adaptive traits, underscoring the evolutionary strategies that enable *P. massoniana* to thrive across southern China.

### Genomic features and gene family expansion

The assembled genome of *P. massoniana* is approximately 21.91 Gb in size, consistent with other conifer genomes, which are known for their large size and high proportion of repetitive sequences. Similar to other pine species, such as *P. tabuliformis* and *P. taeda*, the large genome size of *P. massoniana* is primarily driven by the accumulation of LTR transposable elements, particularly those belonging to the Gypsy and Copia families (Supplementary Table S3) [3, 5]. The expansion of these elements, especially Gypsy LTR-RTs, within the last 5 to 30 million years suggests a relatively recent and rapid increase in their activity (Supplementary Fig. S5). This observation is consistent with the hypothesis that transposable elements can drive genome size increase in plants [3, 4, 81]. The correlation between transposable element bursts, particularly of the Gypsy family, and the accumulation of long introns in the *P. massoniana* genome indicates a significant role for these elements in shaping the genome’s architecture. This phenomenon is not unique to *P. massoniana* but is also observed in other gymnosperm genomes, such as *Cunninghamia lanceolata, P. abies*, and *Torreya grandis* [3–6]. Our data support the notion that intron size in gymnosperms is highly variable and generally longer than in angiosperms (Supplementary Figs. S7 and S8), which may have implications for gene expression and regulation.

### The adaptive evolution of *P. massoniana*

Our analysis revealed significant expansions in several gene families associated with stress responses and plant–pathogen interactions, including genes related to the CYP450 and TPS families (Fig. 3C, D). These gene families are known to be involved in the biosynthesis of secondary metabolites, such as terpenoids, which play critical roles in plant defense mechanisms. The expansion of these gene families in *P. massoniana* suggests that these genes have been under positive selection, likely contributing to the species’ ability to adapt to various biotic and abiotic stresses. The identification of dispersed duplicates and HGT events further highlights the role of gene duplication and foreign gene acquisition in the adaptive evolution of *P. massoniana* (Supplementary Fig. S13). Dispersed duplicate genes, which are scattered throughout the genome rather than being arranged in tandem, have been implicated in conferring adaptive advantages by enabling functional diversification. In our study, we observed signatures of adaptive evolution in these dispersed duplicates, particularly in genes involved in stress responses and metabolic pathways (Supplementary Fig. S9). This suggests that gene duplication has played a critical role in the evolutionary success of *P. massoniana*.

HGT events, although relatively rare in plants, have been increasingly recognized as important contributors to plant evolution. In *P. massoniana*, we identified several potential HGT events involving genes related to stress responses and terpenoid biosynthesis. These findings suggest that the acquisition of foreign genes may have facilitated the evolution of novel traits in *P. massoniana*, further enhancing its adaptability. In our analysis, gene families related to terpene biosynthesis, particularly the *CYP450* and *TPS* families, exhibited significant expansion. These families play crucial roles in the synthesis of oleoresin, which serves not only as a defense mechanism against pests and pathogens but also as a vital economic resource for the timber industry.

### Evolutionary history

The large genome size of *P. massoniana*, predominantly due to a high content of repetitive sequences—particularly LTR retrotransposons—highlights a dynamic evolutionary history. Our results indicate that approximately 81.2% of the genome comprises repetitive elements, with Gypsy retrotransposons making up 43.59%. Such expansions often correlate with environmental adaptations, as transposable elements can drive genomic diversification and contribute to phenotypic variation [3, 4]. The proliferation of LTR retrotransposons in *P. massoniana*, which coincides with the Miocene epoch (Supplementary Fig. S5), aligns with significant climatic changes, such as global temperatures that likely drove adaptation, as shown in previous studies [75, 76].

Furthermore, the identification of 2 WGD events provides a framework for understanding the evolution of gene families in *P. massoniana* (Fig. 2D). WGDs are known to facilitate gene family expansion, allowing species to adapt to new ecological niches [29]. Our discovery of 57,207 duplicated genes (Fig. 2C), mainly resulting from dispersed duplications, supports the notion that genomic redundancy can act as a reservoir for evolutionary innovation, especially in response to biotic and abiotic stressors [82, 83].

### Regulatory networks in oleoresin production

The identification of significant SNP markers associated with oleoresin yield presents valuable opportunities for genomic-assisted breeding. Our study identified 6,064 SNPs linked to oleoresin synthesis (Supplementary Table S11), including those within functional protein families such as kinesin and various transcription factors. Notably, the expansion of the TPS family and the presence of key transcription factors, including PmMYB4, underscore the complexity of the regulatory networks governing oleoresin production.

However, the need for specificity in identifying which *CYP450* and *TPS* genes are involved in oleoresin biosynthesis is crucial. While our findings indicate that PmMYB4 may play a central role in regulating these pathways, further functional validation is necessary to establish definitive connections between these genes and oleoresin yield (Fig. 4). The reliance on expression patterns without functional evidence could lead to misinterpretations. Therefore, future research should employ targeted gene-editing techniques, such as CRISPR/Cas9, to establish direct links between gene function and oleoresin biosynthesis. By focusing on high oleoresin-yielding genotypes and utilizing identified SNP markers along with transcription factors like PmMYB4, we can develop cultivars with enhanced oleoresin production and improved stress resistance.

### Horizontal gene transfer

The presence of horizontally transferred genes in the *P. massoniana* genome adds another layer of complexity to its evolutionary narrative. Our identification of 689 genes likely acquired through horizontal gene transfer (Supplementary Fig. S13; Supplementary Table S9), including those from fungi and bacteria, suggests that *P. massoniana* has benefited from genetic material that enhances its adaptability to environmental stresses (e.g., drought, pest infestations, and pathogen attacks). This phenomenon is consistent with the understanding that HGT can facilitate the acquisition of beneficial traits that promote survival and diversification in changing environments [84].

The functional characterization of these HGT-acquired genes, particularly those involved in stress response and cell wall biosynthesis, warrants further investigation. Understanding how these genes integrate into the existing genetic framework of *P. massoniana* could provide insights into the evolutionary pressures that shaped its current genomic architecture.

### Future directions

Despite the significant insights provided by this study, several areas require further exploration. The specificity of gene functions related to oleoresin biosynthesis needs to be clarified, particularly concerning the roles of various CYP450 and TPS genes. Additionally, the regulatory interactions identified warrant deeper investigation to validate the proposed connections and elucidate the mechanisms underlying oleoresin production. Furthermore, the role of horizontal gene transfer in shaping the adaptive traits of *P. massoniana* should be investigated through functional studies that assess how these genes contribute to stress tolerance and overall fitness. The integration of genomic resources with ecological and physiological studies will provide a more comprehensive understanding of how *P. massoniana* adapts to its environment.

In conclusion, our study advances the understanding of the genomic and evolutionary dynamics of *P. massoniana*, emphasizing its adaptive strategies and the complexity of gene interactions in oleoresin biosynthesis. The insights gained will not only enhance the conservation and management of this ecologically and economically important species but also contribute to the broader understanding of conifer biology and evolution. Future research should focus on addressing the gaps identified in this study to further refine our knowledge of the genetic mechanisms that enable *P. massoniana* to thrive in diverse habitats.

## Additional Files

**Supplementary Fig. S1**. The estimated genome size of *P. massoniana* based on *k*-mer and flow cytometry. (A) The plot of 41-mer coverage-frequency distribution. The blue columns are the actual observed values. The black fitting line is the 41-mer left after removing error parts, and only this part of data is used to estimate genome size. The yellow fitting line comes from the 41-mer distribution in the nonrepeating regions of the genome. The orange-red fitting line corresponds to the 41-mer with low depth, which is generated by sequencing error. The black dotted line is the integral number of coverages of the predicted lowest depth peak. (B) The plots of genome size estimation of *Pinus taeda* and *P. massoniana* based on flow cytometry.

**Supplementary Fig. S2**. Genome assembly pipeline for *P. massoniana*. First, quality control was performed on the raw sequencing data using the SOAPnuke software. Then, Hi-C sequencing data were used to anchor the draft genome with Juicer. 3D-DNA was used for scaffold clustering, sorting, and orientation. BUSCO was used for assembly result evaluation.

**Supplementary Fig. S3**. Transcriptome mapping rate and assessment of gene set completeness by BUSCO. (A) Transcriptome mapping rate of 156 samples of *P. massoniana*. Each bar indicates the transcriptome mapping rate for each sample. (B) The assessment of the gene completeness of 5 gymnosperm genomes by BUSCO. White bars represent percentage of complete; black bars represent percentage of fragmented; gray bars represent percentage of uncovered.

**Supplementary Fig. S4**. Enrichment of the expanded genes in Pinaceae and *P. massoniana*. (A) GO enrichment analysis of expansion genes in Pinaceae. (B) *P. massoniana*. (C) KEGG enrichment analysis of expansion genes in Pinaceae. (D) *P. massoniana*. The red and blue arrows point to the terms associated with plant resistance and growth, respectively. The plots are statistics of term frequencies in (E) GO and (F) KEGG enrichment of *P. massoniana*.

**Supplementary Fig. S5**. Synonymous nucleotide substitution of 4 gymnosperms. (A) Distribution of insertion time calculated by LTR-RTs and (B) dispersed duplication time in *P. massoniana* using mutation rates of 2.2 × 10^−9^ (per base per year). (C) The distribution of insertion time of LTR/Copia. (D) The distribution of insertion time of LTR/Gyspy. (E) The distribution of insertion time of LTR in the intron regions of the 4 species (*P. massoniana, P. tabuliformis, G. biloba*, and *S. giganteum*).

**Supplementary Fig. S6**. The evidence that long intron genes are supported by PacBio data. (A) The full-length transcript that supported ultra-long genes (>20 kb). The different colors refer to the percentage of single-transcript coverage of each gene. The 5 longest genes of (C) *P. massoniana* that had a similar exon–intron structure as their (B) *A. thaliana* homologs.

**Supplementary Fig. S7**. Relationship between intron, gene expression, and gene family size. (A) Distribution among gene family size, intron length, and intron number of *A. thaliana* and *P. massoniana*. (B) Distribution among gene family size and gene average expression, maximum expression, and expression breadth of *P. massoniana*.

**Supplementary Fig. S8**. The average lengths of CDSs, exons, and introns of 13 species used in research. (A) Boxplots of CDS lengths, (B) exon lengths, and (C) intron lengths of 13 species. The black vertical bar in the center of the box represents the mean value, the upper and lower limits of the box indicate the upper and lower quantiles, and the whiskers correspond to the data range within 1.5× the interquartile range. Different colors in boxes represent angiosperms, gymnosperms, and algaes (outgroup).

**Supplementary Fig. S9**. Resistance genes in *P. massoniana*. (A) Phylogenetic tree of NLR genes identified in *P. massoniana*. (B) Transcriptional correlation of R-gene pairs in dispersed (107 pairs) and tandem (65 pairs) duplication, as well as genome gene pairs in WGD (410 pairs), tandem (13,088 pairs), and dispersed (13,358 pairs) duplication and random (100 pairs) gene pairs. The 2 groups with significant difference are marked with “*.” (C) The number of transcription factors associated with resistance and growth identified in 4 gymnosperms and *A. thaliana*. (D) Phylogenetic tree of lignin biosynthesis-related genes identified in *P. massoniana*.

**Supplementary Fig. S10**. Correlation between transcription factor ratio and genome size. (A) Dotplot shows correlation between transcription factors identified in 13 species and their genome size. (B) Different colors in dots represent angiosperms, gymnosperms, and algaes (outgroup).

**Supplementary Fig. S11**. *WRKY* and *AP*2 genes in *P. massoniana*. Phylogenetic tree of (A) *WRKY* and (B) *AP*2 genes identified in *P. massoniana*. Different color blocks represent different subclusters of *WKRY* and *AP*2 genes.

**Supplementary Fig. S12**. The expression profiles of *WRKY* and *AP*2 genes of *P. massoniana* under different stress or treatment. The colors represent the different absolute expression levels, as illustrated by the legend.

**Supplementary Fig. S13**. Examples of horizontally acquired genes in *P. massoniana*. Phylogenetic tree of (A) phosphoglycerate kinases (*PGK*), (B) ABC family transporter proteins, (C) carbohydrate active enzymes (CAZymes), (D) glycoside hydrolases, and (E) *NRT*1 genes. Numbers beside branches represent bootstrap values from neighbor-joining algorithm. (F) Phenotype of *WT* and *PGK* OE‐2, OE‐3, OE‐5, and OE‐8 under 5-day salt stress. Bar = 4 cm.

**Supplementary Fig. S14**. RNA *in situ* hybridization analyses of 7 selected key genes with distinct signals. Scale bar = 50 µm; PmAACT: Gmmutg963G000050.1; PmHMGS: Gmmutg64965G000010.1; PmMK: Gmmutg51494G000020.2; PmDXR: Gmmutg2453G000010.1; PmMCT: Gmmutg46752G000010.1; PmMDS: Gmmutg12323G000030.1; PmHDS: Gmmutg24519G000020.1. C: cortex; H: hypodermis cell; M: mesophyll cell; P: pith; Ph: phloem; R: ray cell; S: sclerenchyma; SS: sunken stoma; T: transfusion tissue; X: xylem resin cells.

**Supplementary Fig. S15**. Population structure of 204 Masson pines. (A) Principal component analysis scatterplot. All genotypes were grouped in 3 clusters: west, south, and southeast. (B) Model-based Bayesian clustering of 204 Masson pines performed using ADMIXTURE with the number of ancestry kinships (*K*) set to 1–6. Each group is denoted by a vertical bar composed of different colors in proportions corresponding to its proportion of genetic ancestry from each of these ancestral populations. (C) Phylogenetic ML tree of the 204 Masson pines based on the 503,296 SNPs. (D) Diversity indices (π) and the population differentiation statistic (*F_ST_*) of 3 groups of *P. massoniana*.

**Supplementary Table S1**. Statistics of *P. massoniana* genome assembly.

**Supplementary Table S2**. Statistics of chromosome level genome assembly by using Hi-C.

**Supplementary Table S3**. Statistics of annotation result of repeated sequence in *P. massoniana*.

**Supplementary Table S4**. Details of the transcriptome data of *P. massoniana*.

**Supplementary Table S5**. Gene structure variation across the 13 species used in this study.

**Supplementary Table S6**. Syntenic blocks between *P. massoniana* and *P. tabuliformi*.

**Supplementary Table S7**. Functional enrichment of the expanded genes in *P. massoniana*.

**Supplementary Table S8**. Correlation between intact-TE content, genome size, intron length, and exon length.

**Supplementary Table S9**. Horizontal transfer genes identified in *P. massoniana*.

**Supplementary Table S10**. The primer sequences for the 10 genes in the resin terpene biosynthesis pathway.

**Supplementary Table S11**. The associated *P450* and *TPS* genes detected by GWAS.

giaf056_Supplemental_Files

giaf056_Authors_Response_To_Reviewer_Comments_original_submission

giaf056_GIGA-D-24-00472_original_submission

giaf056_GIGA-D-24-00472_Revision_1

giaf056_Reviewer_1_Report_original_submission Nian Wang -- 1/15/2025

giaf056_Reviewer_2_Report_original_submission Yang Dong, Ph.D -- 1/16/2025

## Abbreviations

ABC: ATP-binding cassette; AI: Alien Index; BIC: Bayesian information criterion; BLAST: Basic Local Alignment Search Tool; BUSCO: Benchmarking Universal Single-Copy Orthologs; CDS: coding sequence; DAB: 3,3-diaminobenzidine-4 HCl; DIG: digoxigenin; EMSA: electrophoretic mobility shift assay; gDNA: genomic DNA; GO: Gene Ontology; GPP: geranyl diphosphate; GWAS: genome-wide association study; HGT: horizontal gene transfer; IPP: isopentenyl pyrophosphate; iTOL: Interactive Tree Of Life; KEGG: Kyoto Encyclopedia of Genes and Genomes; LTR: long terminal repeat; LTR-RT: long terminal repeat retrotransposon; LUC: luciferase; MAF: minor allele frequency; MeJA: methyl jasmonate; ML: maximum likelihood; MLM: mixed linear model; MRCA: most recent common ancestor; MYA: million years ago; NGS: next-generation sequencing; OST: N-oligosaccharyl transferase; PCA: principal component analysis; PGK: phosphoglycerate kinase; RT-qPCR: quantitative reverse transcription PCR; SNP: single nucleotide polymorphism; TE: transposable element; TF: transcription factor; TPS: terpene synthase; TWAS: Transcriptome-Wide Association Study; WGD: whole-genome duplication.

## Data Availability

The genomic and transcriptomic sequence data generated in this study are available under NCBI BioProject accession PRJNA1240911. The genomic and transcriptomic sequence data reused in this study are available under the NCBI BioProject accessions: PRJNA561037, PRJNA576798, PRJNA595650, PRJNA637602, PRJNA636599, PRJNA647052, PRJNA648715, PRJNA655997, PRJNA656380, PRJNA693351, PRJNA707606, PRJNA743934, PRJNA744619, PRJNA748221, PRJNA749363, PRJNA781761, PRJNA863936, and PRJNA667166. All additional supporting data are available in the *GigaScience* repository, GigaDB [85].
