# Supplementary material for: Chromosome-level genome assembly of Pinus massoniana provides insights into conifer adaptive evolution
Source: Gigascience. 2025 May 30;14:giaf056. doi: 10.1093/gigascience/giaf056 (PMC12122422; doi:10.1093/gigascience/giaf056)
Supplement: giaf056_Supplemental_Files [file giaf056_supplemental_files.zip › Supplemental Materials_04182025.docx]

**Supplemental materials for** **“Chromosome-Level Genome Assembly of *Pinus massoniana* provides insights into Conifer Adaptive Evolution”**

Hu Chen^1,2,3†^, Xinghu Qin^4,5,7,8†^, Yinghao Chen^1,2,3†^, Haoyu Zhang^4,5,6^, Yuanheng Feng^1,2,3^, Jianhui Tan^1,2,3^, Xinhua Chen^1,2,3^, La Hu^1,2,3^, Junkang Xie^1,2,3^, Jianbo Xie^4,5,6*^, Zhangqi Yang^1,2,3*^

^1^Key Laboratory of National Forestry and Grassland Administration on Cultivation of Fast-Growing Timber in Central South China, Guangxi Forestry Research Institute

^2^Guangxi Key Laboratory of Superior Timber Trees Resource Cultivation, Guangxi Forestry Research Institute

^3^Guangxi Key Laboratory of Special Non-wood Forests Cultivation and Utilization, Guangxi Forestry Research Institute

^4^State Key Laboratory of Tree Genetics and Breeding, College of Biological Sciences and Technology, Beijing Forestry University, Beijing 100083, China

^5^National Engineering Research Center of Tree Breeding and Ecological Restoration, College of Biological Sciences and Technology, Beijing Forestry University, Beijing 100083, China

^6^The Tree and Ornamental Plant Breeding and Biotechnology Laboratory of National Forestry and Grassland Administration, Beijing Forestry University, Beijing 100083, China

^7^School of Ecology and Nature Conservation, Beijing Forestry University & The Capital Biodiversity Conservation Institute, Beijing 100083, China

^8^ China (BJFU) -UK (St Andrews) International Joint Machine Learning Laboratory for Biodiversity Research, Beijing Forestry University, Beijing 100083, China.

^†^These authors contributed equally to this work.

## To whom correspondence should be addressed: Zhangqi Yang: [yangzhangqi@163.com](mailto:yangzhangqi@163.com); Correspondence may also be addressed to Jianbo Xie, Email: [jbxie@bjfu.edu.cn](mailto:jbxie@bjfu.edu.cn); Tel: +86-10-62336007; Fax: +86-10-62336164.

**Supplemental Figures**

**Figure S1. The estimated genome size of *P. massoniana* based on K-mer and flow cytometry.**

(A) The plot of 41-mer coverage-frequency distribution. The blue columns are the actual observed values. The black fitting line is the 41-mer left after removing error parts, and only this part of data is used to estimate genome size. The yellow fitting line comes from the 41-mer distribution in the non-repeating regions of the genome. The orange-red fitting line corresponds to the 41-mer with low depth, which generated by sequencing error. The black dotted line is the integral number of coverages of the predicted lowest depth peak. (B) The plots of genome size estimation of *Pinus taeda* and *P. massoniana* based on flow cytometry.

**Figure S2. Genome assembly pipeline for *P. massoniana*.**

First, perform quality control on the raw sequencing data using the SOAPnuke software. Then, Hi-C sequencing data were used to anchor the draft genome with Juicer. 3D-DNA was used for scaffolds clustering, sorting and orientation. And BUSCO was used for assembly result evaluation.

**Figure S3. Transcriptome mapping rate and assessment of gene set completeness by BUSCO*.***

(A) Transcriptome mapping rate of 156 samples of *P. massoniana*. Each bar indicates the transcriptome mapping rate for each sample. (B) The assessment of the gene completeness of five gymnosperms genomes by BUSCO. White bars represent percentage of Complete; black bars represent percentage of fragmented; grey bars represent percentage of uncovered.

**Figure S4. Enrichment of the expanded genes in Pinaceae and *P. massoniana.*** (A) GO enrichment analysis of expansion genes in Pinaceae. (B) *P. massoniana*. (C) KEGG enrichment analysis of expansion genes in Pinaceae. D. *P. massoniana*. The red and blue arrows point to the terms associated with plant resistance and growth, respectively. The plots are statistics of term frequencies in E. GO and F. KEGG enrichment of *P. massoniana*.

**Figure S5. Synonymous nucleotide substitution of four gymnosperms*.*** (A) Distribution of insertion time calculated by LTR-RTs and (B) dispersed duplication time in *P. massoniana* using mutation rates of 2.2 × 10^-9^ (per base per year). (C) The distribution of insertion time of LTR/Copia. D. The distribution of insertion time of LTR/Gyspy. E. The distribution of insertion time of LTR in the intron regions of the four species (*P. massoniana, P. tabuliformis,* *G. biloba,* and *S. giganteum*).

**Figure S6. The evidence that long intron genes are supported by Pacbio data*.* (**A) The full-length transcript that supported ultra-long genes (>20 kb). The different colors refer to the percentage of single transcript coverage of each gene. The five longest genes of (C) *P. massoniana* that had similar exon-intron structure as their (B) *A. thaliana* homologs.

**Figure S7. Relationship among intron, gene expression and gene family size.** (A) Distribution among gene family size and intron length, intron number of *A. thaliana* and *P. massoniana*. B. Distribution among gene family size and gene average expression, maximum expression, expression breadth of *P. massoniana*.

**Figure S8. The average lengths of CDSs, exons and introns of 13 species used in research*.*** (A) Boxplots of CDSs lengths, (B) exons lengths and C. introns lengths of 13 species. The black vertical bar in the centre of the box represents the mean value, the upper and lower limits of the box indicate the upper and lower quantiles, and the whiskers correspond to the data range within 1.5× the interquartile range. Different colors filled in boxes represent angiosperms, gymnosperms and algaes (outgroup).

**Figure S9. Resistance genes in *P. massoniana.*** (A) Phylogenetic tree of NLR genes identified in *P. massoniana*. (B) Transcriptional correlation of R-gene pairs in dispersed (107 pairs) and tandem (65 pairs) duplication, and genome gene pairs in WGD (410 pairs), tandem (13,088 pairs), dispersed (13,358pairs) duplication and random (100 pairs) gene pairs. The two groups with significant difference were marked with “*”. (C) The number of transcription factors associated with resistance and growth identified in four gymnosperms and *A. thaliana*. D. Phylogenetic tree of lignin biosynthesis related genes identified in *P. massoniana*.

**Figure S10. Correlation between transcription factors ratio and genome size.** (A) Dotplot shows correlation between transcription factors identified in 13 species and their genome size. (B) Different colors filled in dots represent angiosperms, gymnosperms and algaes (outgroup).

**Figure S11. *WRKY* and *AP*2 genes in *P. massoniana*.** Phylogenetic tree of (A) *WRKY* and (B) *AP*2 genes identified in *P. massoniana*. Different color blocks represent different sub-clusters of *WKRY* and *AP*2 genes.

**Figure S12. The expression profiles of *WRKY* and *AP*2 genes of *P. massoniana* under different stress or treatment.** The colors represent the different absolute expression levels as illustrated by the legend.

**Figure S13. Examples of horizontally acquired genes in *P. massoniana*.** Phylogenetic tree of (A) phosphoglycerate kinases (*PGK*), (B) ABC family transporter proteins, (C) carbohydrate active enzymes (CAZymes), (D) glycoside hydrolases and (E) *NRT*1 genes. Numbers beside branches represent bootstrap values from Neighbor-Joining Algorithm. (F) Phenotype of *WT*, and *PGK* OE‐2, OE‐3, OE‐5 and OE‐8 under 5-days salt stress. Bar = 4 cm.

**Figure S14. RNA in situ hybridization analyses of seven selected key genes with distinct signals.** Scale bar= 50 μm; PmAACT: Gmmutg963G000050.1; PmHMGS: Gmmutg64965G000010.1; PmMK: Gmmutg51494G000020.2; PmDXR: Gmmutg2453G000010.1; PmMCT: Gmmutg46752G000010.1; PmMDS: Gmmutg12323G000030.1; PmHDS: Gmmutg24519G000020.1. H: hypodermis cell; M: mesophyll cell; P: pith; R: ray cell; SS: sunken stoma; S: sclerenchyma; T: transfusion tissue; C: cortex; Ph: phloem; X: xylem resin cells.

**Figure S15. Population structure of 204 Masson pines*.*** (A) Principal component analysis scatter plot. All genotypes were grouped in three clusters: West, South, South east. (B) Model-based Bayesian clustering of 204 Masson pines performed using ADMIXTURE with the number of ancestry kinships (*K*) set to 1-6. Each group is denoted by a vertical bar composed of different colors in proportions corresponding to its proportion of genetic ancestry from each of these ancestral populations. (C) Phylogenetic maximum likelihood (ML) tree of the 204 Masson pines based on the 503,296 SNPs. (D) Diversity indices (π) and the population differentiation statistic (*F_ST_*) of three groups of *P. massoniana*.

**Supplemental Tables**

Table S1. Statistics of *P. massoniana* genome assembly.

Table S2. Statistics of chromosome level genome assembly by using Hi-C.

Table S3. Statistics of annotation result of repeated sequence in *P. massoniana*.

Table S4. Details of the transcriptome data of *P. massoniana*.

Table S5. Gene structure variation across the 13 species used in this study.

Table S6. Syntenic blocks between *P. massoniana* and *P. tabuliformi*.

Table S7. Functional enrichment of the expanded genes in *P. massoniana*.

Table S8. Correlation between intact-TE content, genome size, intron length and exon length.

Table S9. Horizontal transfer genes identified in *P. massoniana*.

Table S10. The primer sequences for the ten genes in resin terpene biosynthesis pathway.

Table S11. The associated *P450* and *TPS* genes detected by GWAS.


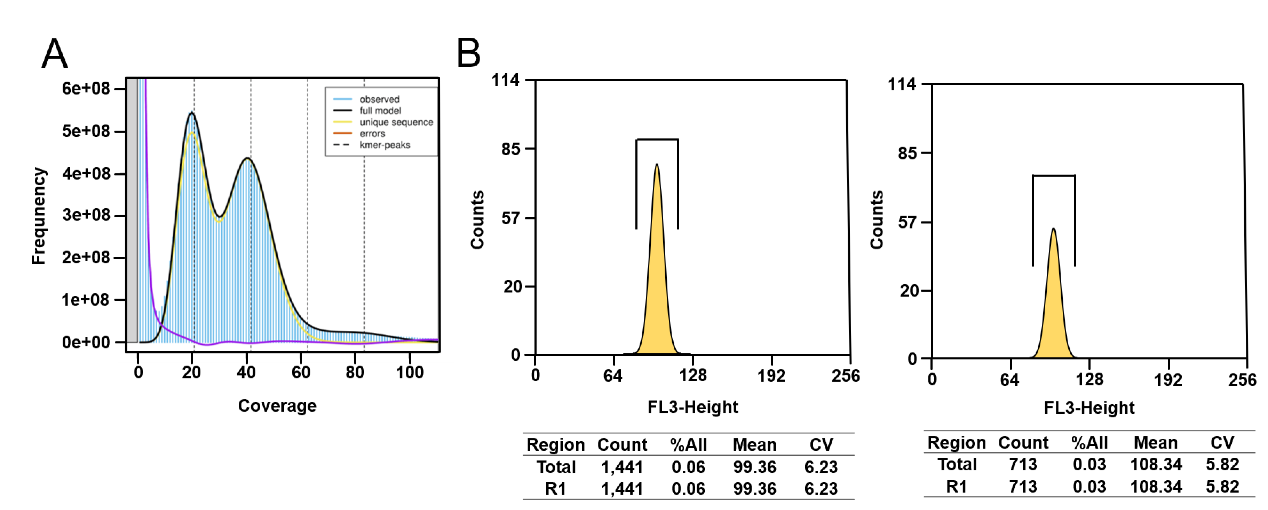


**Figure S1. The estimated genome size of *P. massoniana* based on K-mer and flow cytometry.**

(A) The plot of 41-mer coverage-frequency distribution. The blue columns are the actual observed values. The black fitting line is the 41-mer left after removing error parts, and only this part of data is used to estimate genome size. The yellow fitting line comes from the 41-mer distribution in the non-repeating regions of the genome. The orange-red fitting line corresponds to the 41-mer with low depth, which generated by sequencing error. The black dotted line is the integral number of coverages of the predicted lowest depth peak. (B) The plots of genome size estimation of *Pinus taeda* and *P. massoniana* based on flow cytometry.


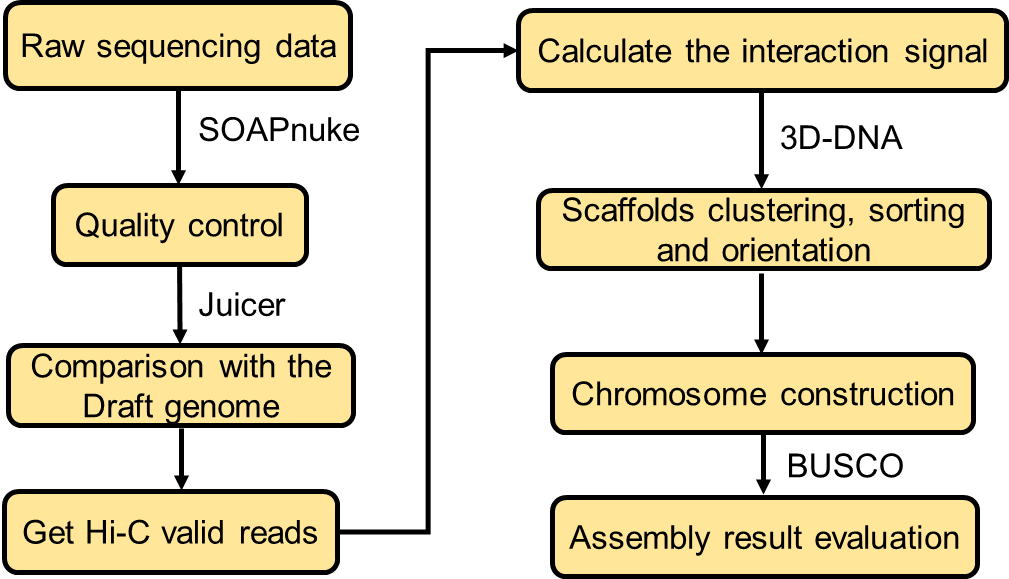


**Figure S2. Genome assembly pipeline for *P. massoniana*.**

First, perform quality control on the raw sequencing data using the SOAPnuke software. Then, Hi-C sequencing data were used to anchor the draft genome with Juicer. 3D-DNA was used for scaffolds clustering, sorting and orientation. And BUSCO was used for assembly result evaluation.


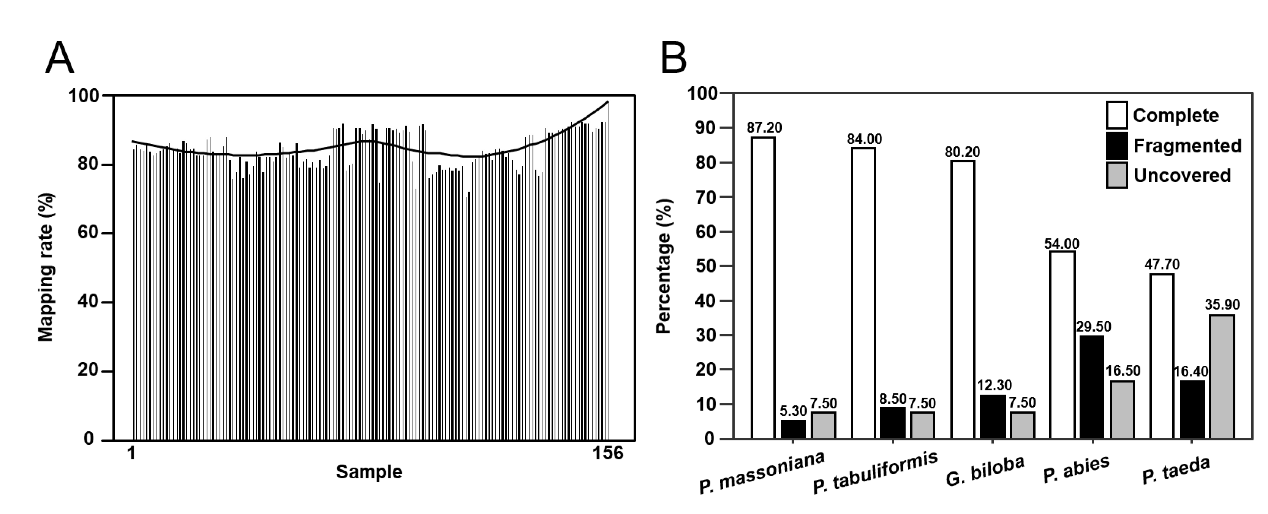


**Figure S3. Transcriptome mapping rate and assessment of gene set completeness by BUSCO*.***

(A) Transcriptome mapping rate of 156 samples of *P. massoniana*. Each bar indicates the transcriptome mapping rate for each sample. (B) The assessment of the gene completeness of five gymnosperms genomes by BUSCO. White bars represent percentage of Complete; black bars represent percentage of fragmented; grey bars represent percentage of uncovered.


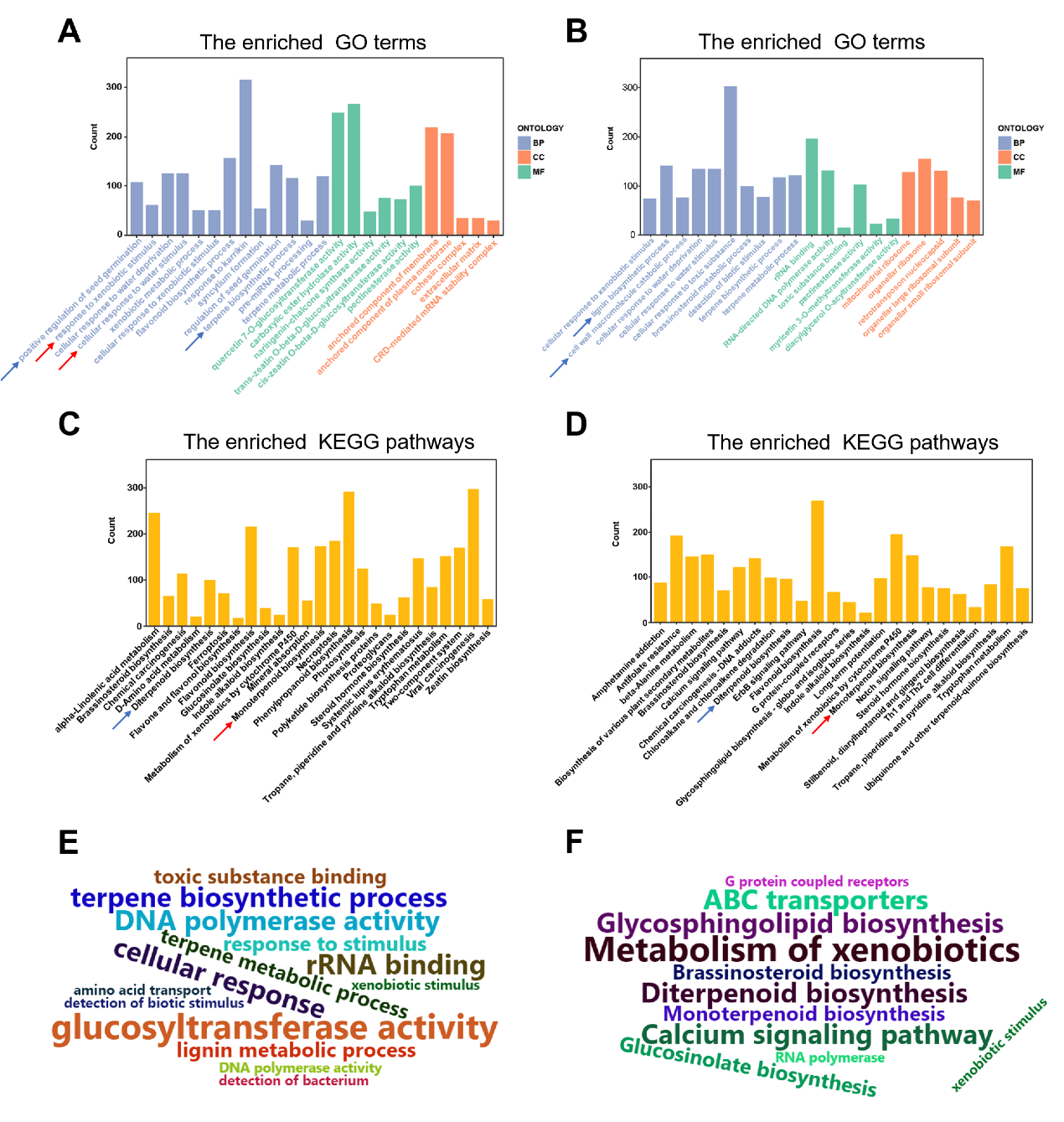


**Figure S4. Enrichment of the expanded genes in Pinaceae and *P. massoniana.*** (A) GO enrichment analysis of expansion genes in Pinaceae. (B) *P. massoniana*. (C) KEGG enrichment analysis of expansion genes in Pinaceae. D. *P. massoniana*. The red and blue arrows point to the terms associated with plant resistance and growth, respectively. The plots are statistics of term frequencies in E. GO and F. KEGG enrichment of *P. massoniana*.


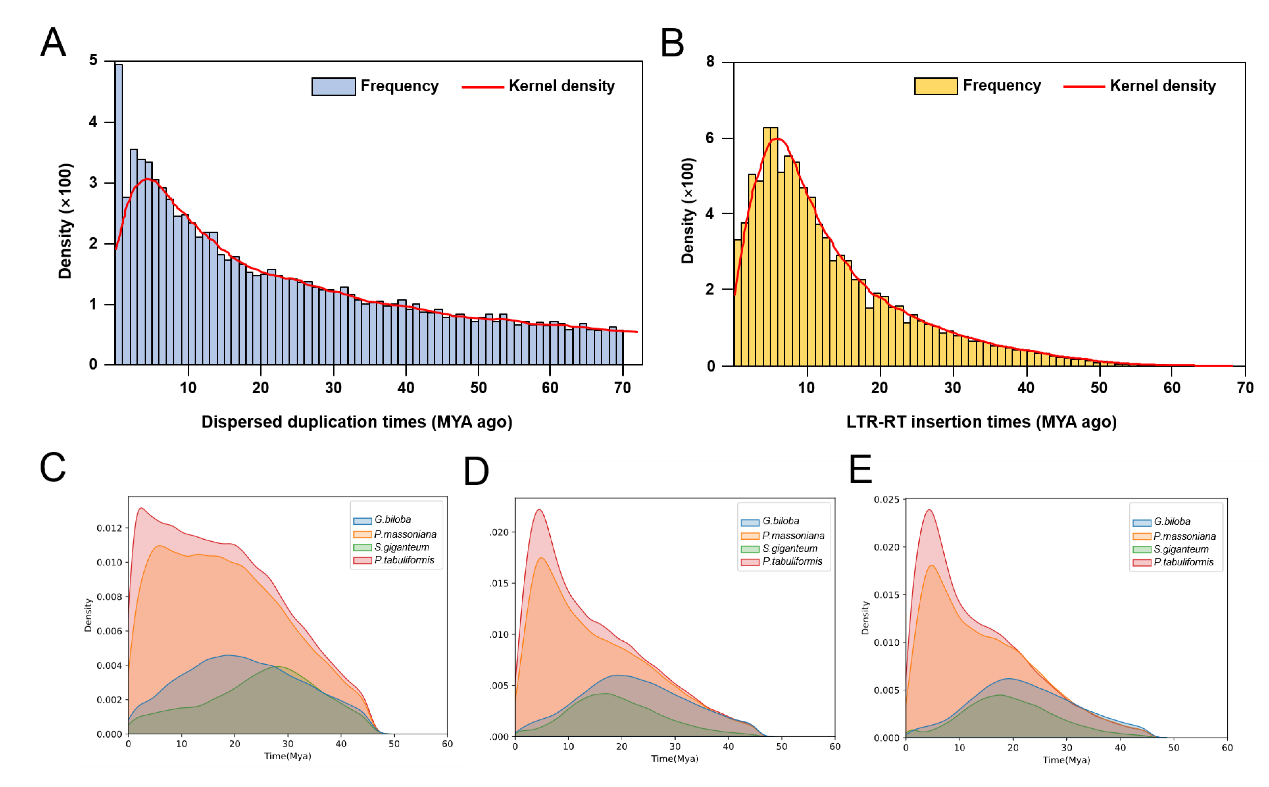


**Figure S5. Synonymous nucleotide substitution of four gymnosperms*.*** (A) Distribution of insertion time calculated by LTR-RTs and (B) dispersed duplication time in *P. massoniana* using mutation rates of 2.2 × 10^-9^ (per base per year). (C) The distribution of insertion time of LTR/Copia. D. The distribution of insertion time of LTR/Gyspy. E. The distribution of insertion time of LTR in the intron regions of the four species (*P. massoniana, P. tabuliformis,* *G. biloba,* and *S. giganteum*).


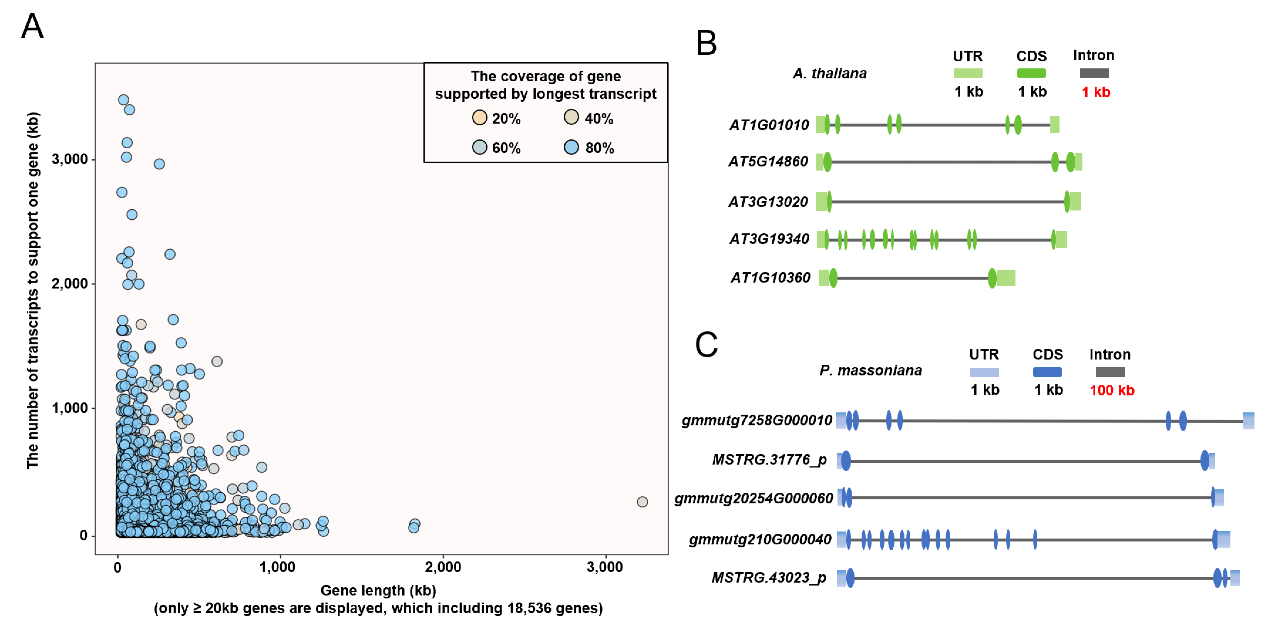


**Figure S6. The evidence that long intron genes are supported by Pacbio data*.* (**A) The full-length transcript that supported ultra-long genes (>20 kb). The different colors refer to the percentage of single transcript coverage of each gene. The five longest genes of (C) *P. massoniana* that had similar exon-intron structure as their (B) *A. thaliana* homologs.


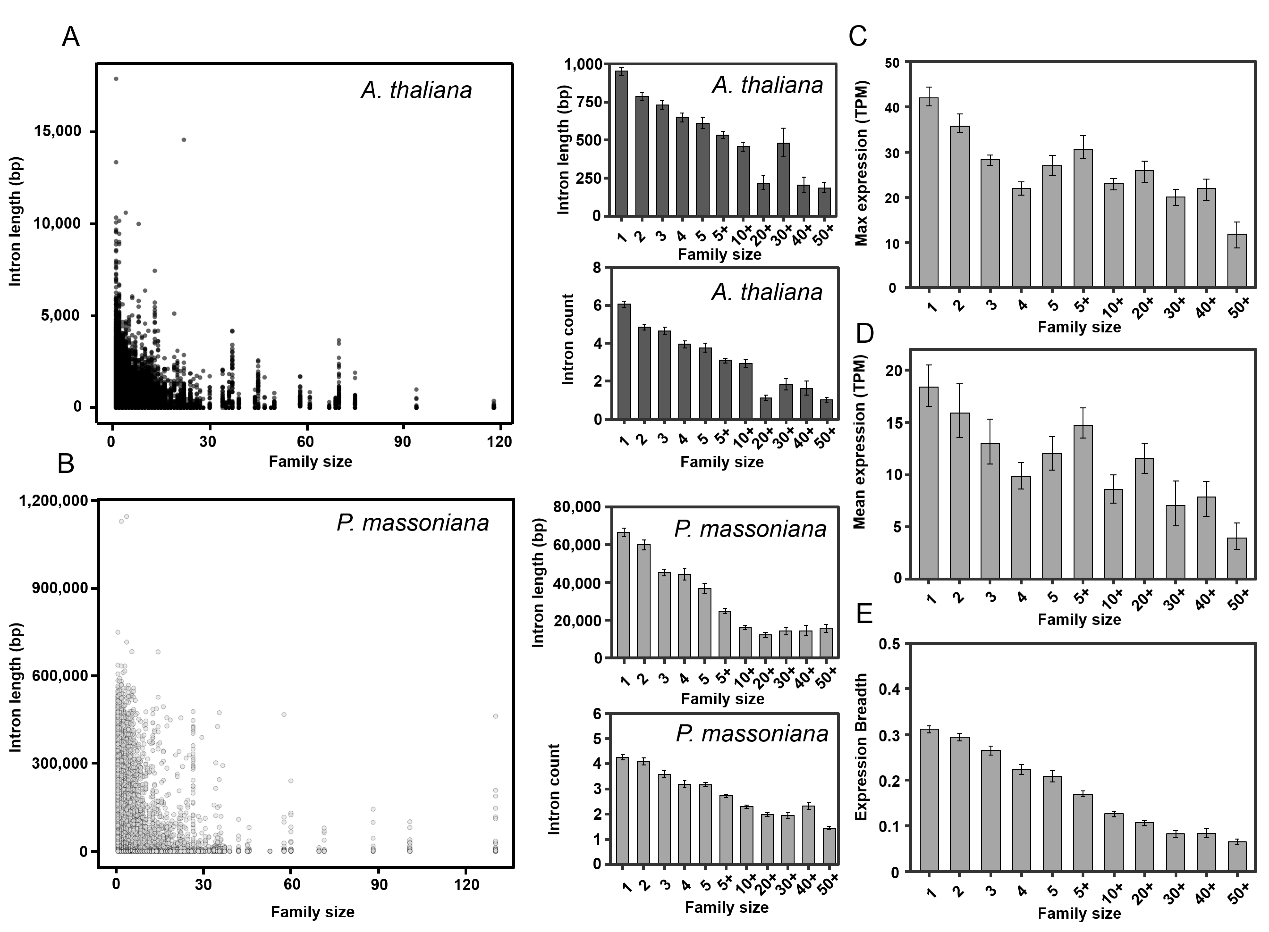


**Figure S7. Relationship among intron, gene expression and gene family size.** (A) Distribution among gene family size and intron length, intron number of *A. thaliana* and *P. massoniana*. B. Distribution among gene family size and gene average expression, maximum expression, expression breadth of *P. massoniana*.


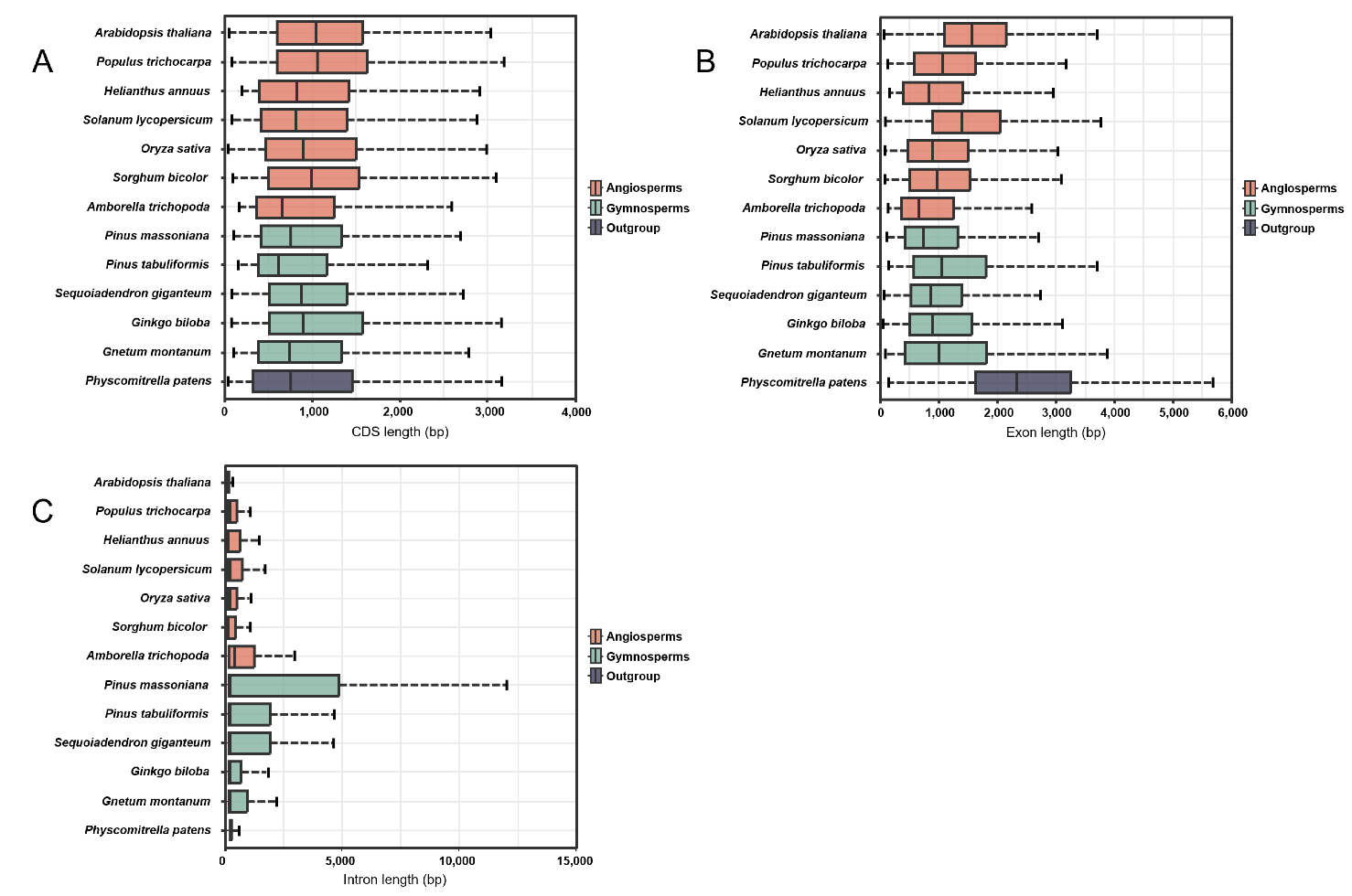


**Figure S8. The average lengths of CDSs, exons and introns of 13 species used in research*.*** (A) Boxplots of CDSs lengths, (B) exons lengths and (C) introns lengths of 13 species. The black vertical bar in the center of the box represents the mean value, the upper and lower limits of the box indicate the upper and lower quantiles, and the whiskers correspond to the data range within 1.5× the interquartile range. Different colors filled in boxes represent angiosperms, gymnosperms and algaes (outgroup).


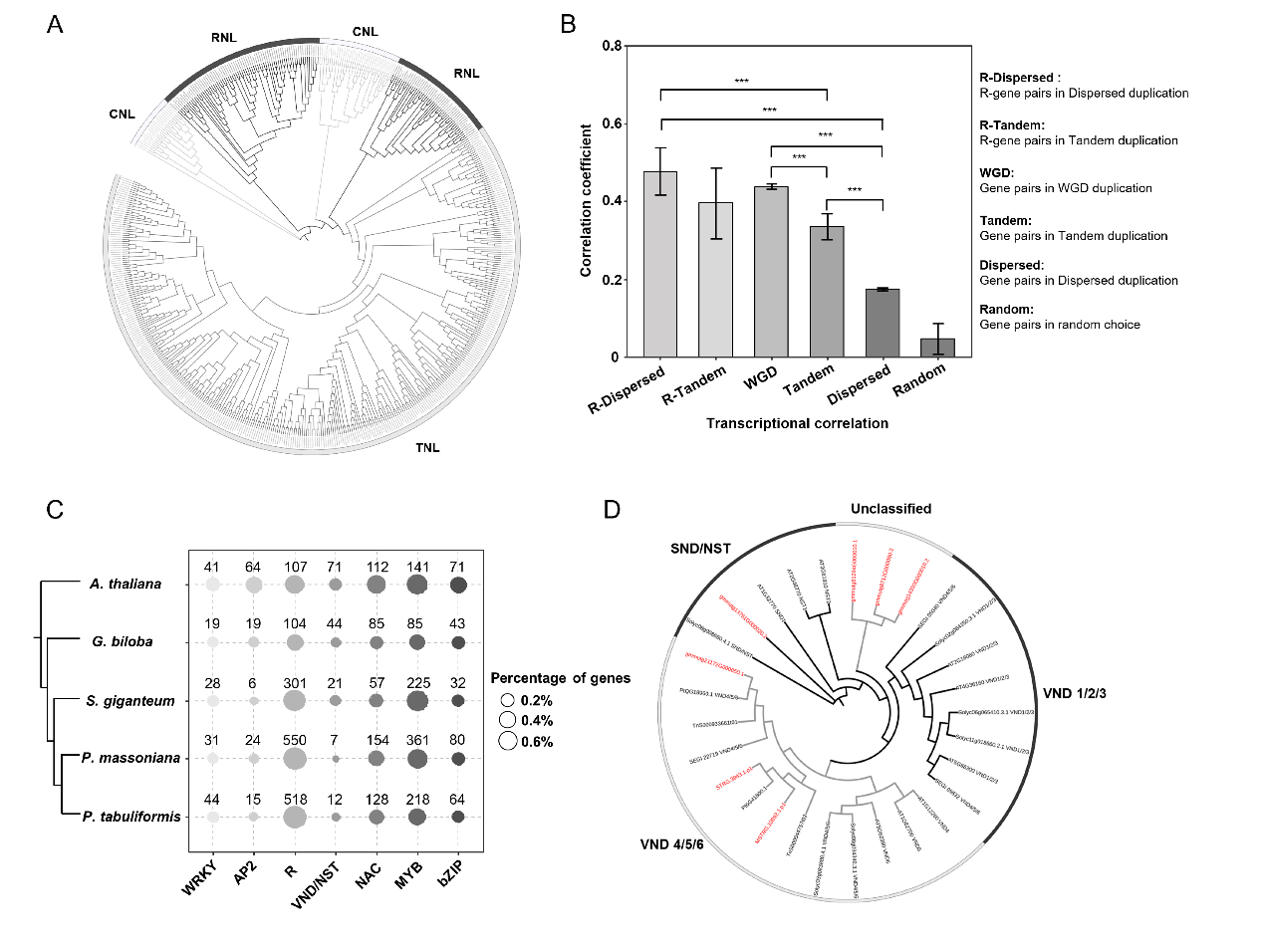


**Figure S9. Resistance genes in *P. massoniana.*** (A) Phylogenetic tree of NLR genes identified in *P. massoniana*. (B) Transcriptional correlation of R-gene pairs in dispersed (107 pairs) and tandem (65 pairs) duplication, and genome gene pairs in WGD (410 pairs), tandem (13,088 pairs), dispersed (13,358 pairs) duplication and random (100 pairs) gene pairs. The two groups with significant difference were marked with “*”. (C) The number of transcription factors associated with resistance and growth identified in four gymnosperms and *A.thaliana*. D. Phylogenetic tree of lignin biosynthesis related genes identified in *P. massoniana*.


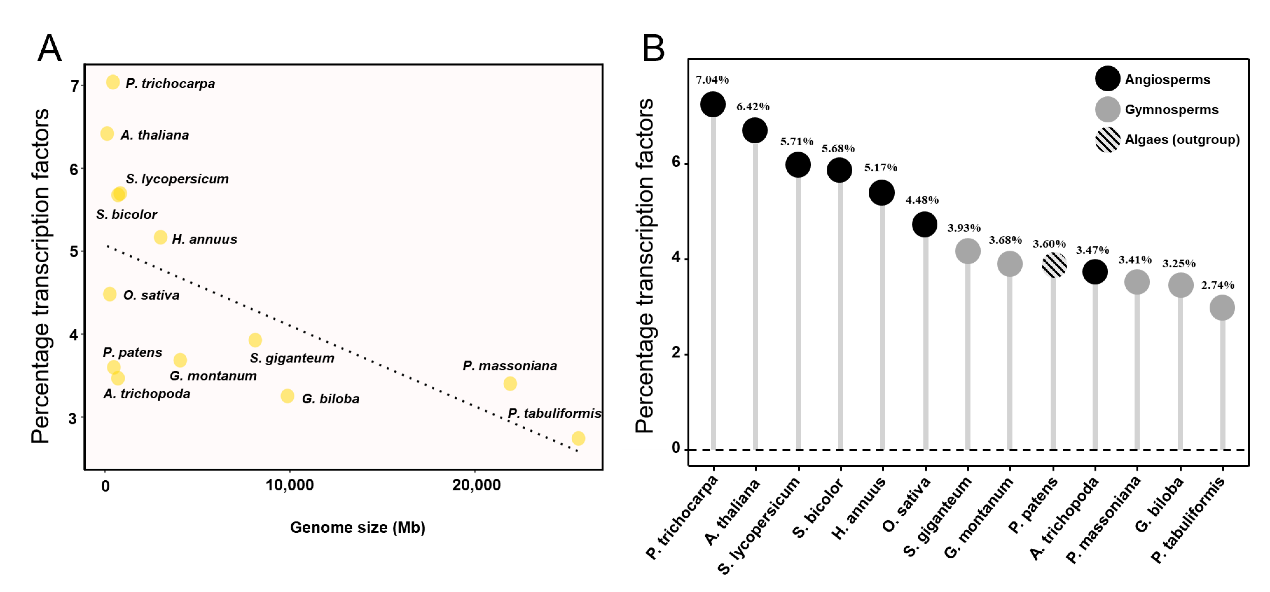


**Figure S10. Correlation between transcription factors ratio and genome size.** (A) Dotplot shows correlation between transcription factors identified in 13 species and their genome size. (B) Different colors filled in dots represent angiosperms, gymnosperms and algaes (outgroup).


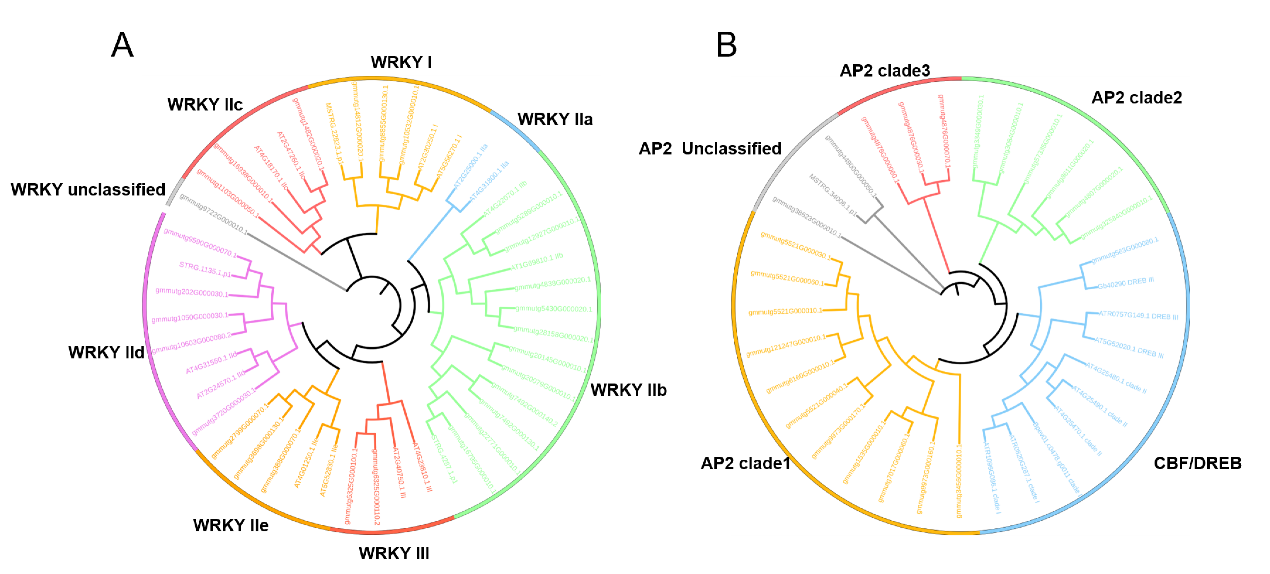


**Figure S11. WRKY and AP2 genes in *P. massoniana*.** Phylogenetic tree of (A) *WRKY* and (B) *AP*2 genes identified in *P. massoniana*. Different color blocks represent different sub-clusters of *WKRY* and *AP2* genes.


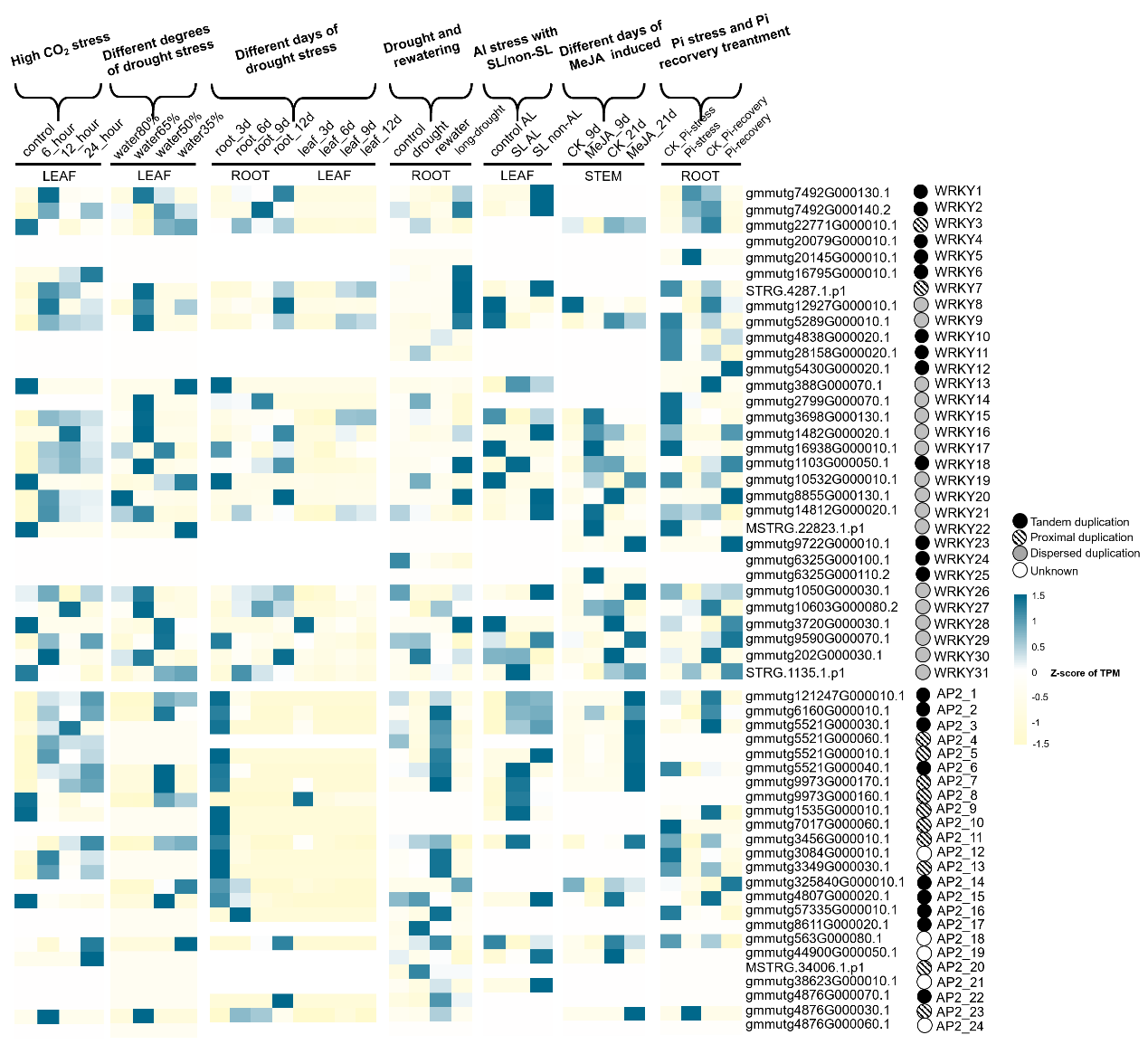


**Figure S12.** The expression profiles of *WRKY* and *AP*2 genes of *P. massoniana* under different stress or treatment. The colors represent the different absolute expression levels as illustrated by the legend.


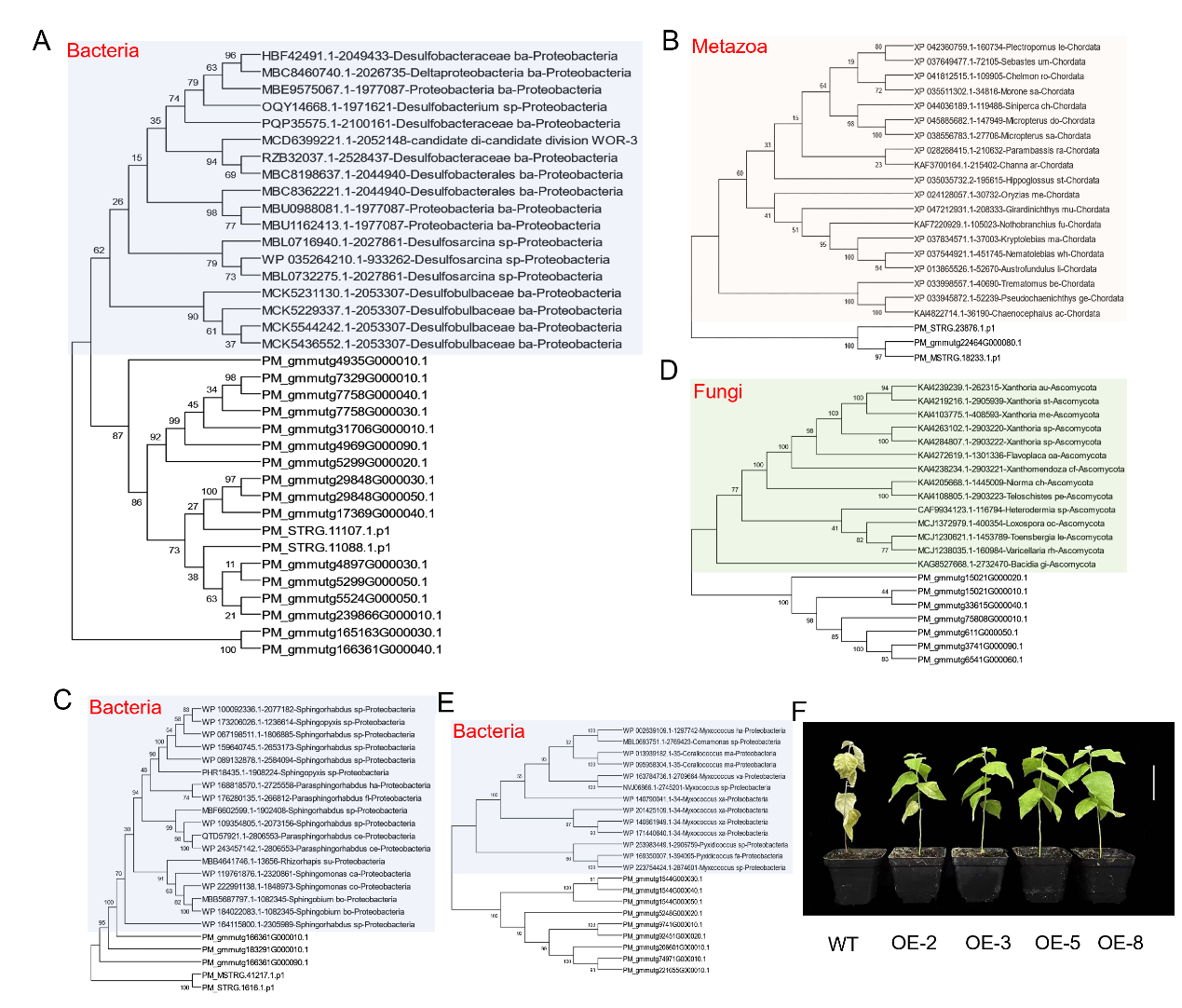


**Figure S13. Examples of horizontally acquired genes in *P. massoniana*.** Phylogenetic tree of (A) phosphoglycerate kinases (*PGK*), (B) ABC family transporter proteins, (C) carbohydrate active enzymes (CAZymes), (D) glycoside hydrolases and (E) *NRT*1 genes. Numbers beside branches represent bootstrap values from Neighbor-Joining Algorithm. (F) Phenotype of *WT*, and *PGK* OE‐2, OE‐3, OE‐5 and OE‐8 under 5-days salt stress. Bar = 4 cm.


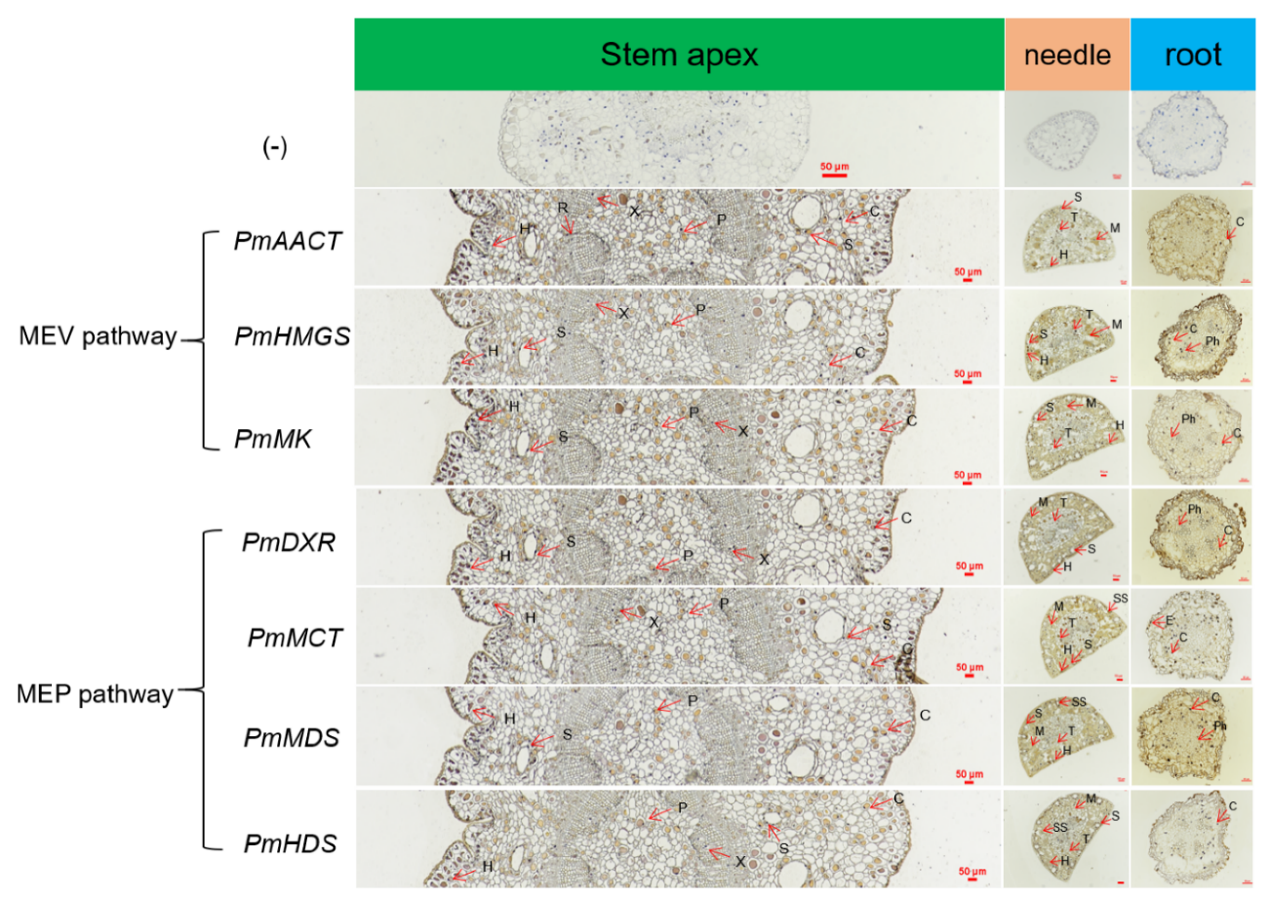


**Figure S14. RNA in situ hybridization analyses of seven selected key genes with distinct signals.** Scale bar= 50 μm; *PmAACT*: *Gmmutg963G000050.1*; *PmHMGS*: *Gmmutg64965G000010.1*; *PmMK*: *Gmmutg51494G000020.2*; *PmDXR*: *Gmmutg2453G000010.1*; *PmMCT*: *Gmmutg46752G000010.1*; *PmMDS*: *Gmmutg12323G000030.1*; *PmHDS*: *Gmmutg24519G000020.1*. H: hypodermis cell; M: mesophyll cell; P: pith; R: ray cell; SS: sunken stoma; S: sclerenchyma; T: transfusion tissue; C: cortex; Ph: phloem; X: xylem resin cells.


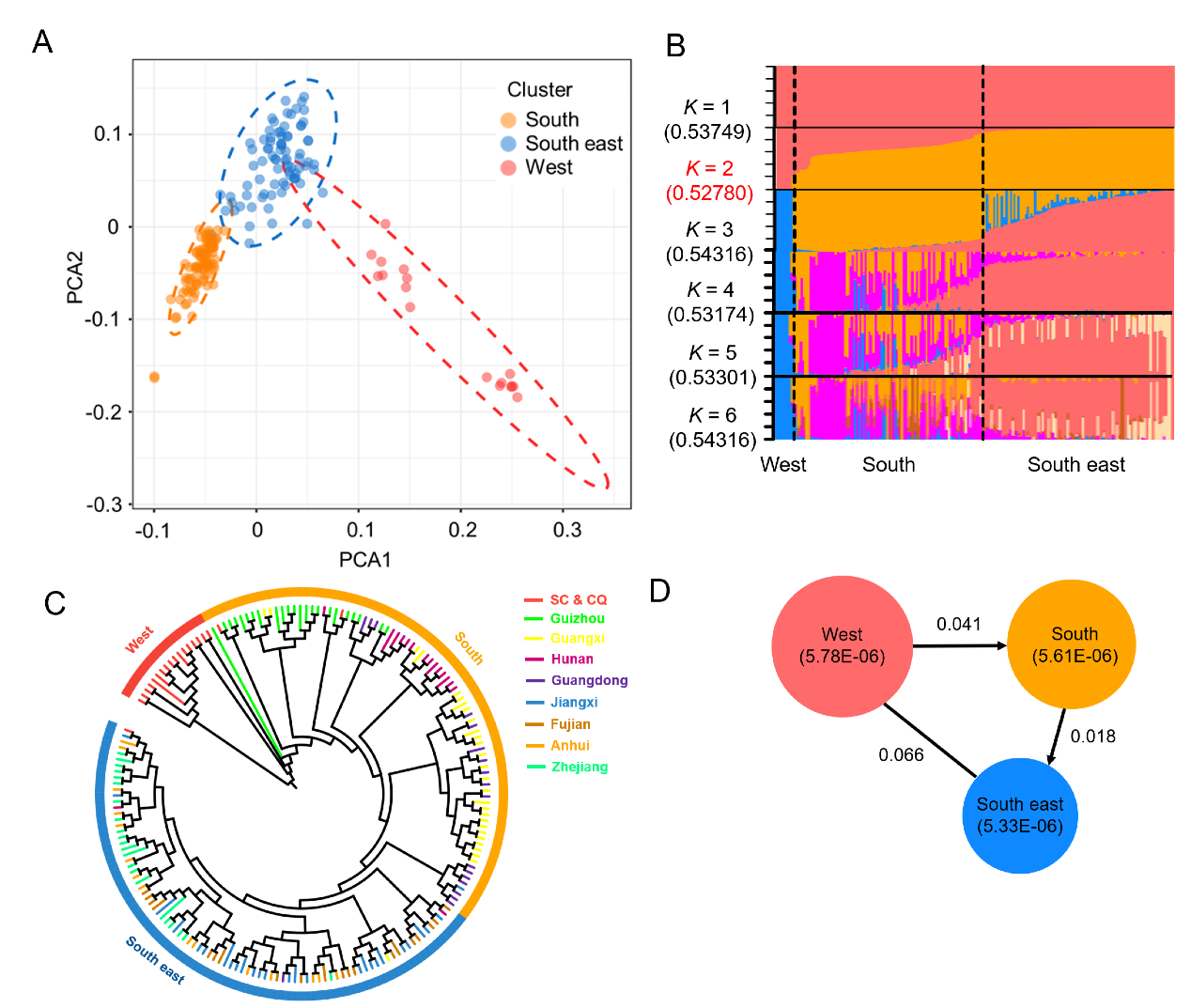


**Figure S15. Population structure of 204 Masson pines*.*** (A) Principal component analysis scatter plot. All genotypes were grouped in three clusters: West, South, South east. (B) Model-based Bayesian clustering of 156 Masson pines performed using ADMIXTURE with the number of ancestry kinships (*K*) set to 1-6. Each group is denoted by a vertical bar composed of different colors in proportions corresponding to its proportion of genetic ancestry from each of these ancestral populations. (C) Phylogenetic maximum likelihood (ML) tree of the 204 Masson pines based on the 503,296 SNPs. (D) Diversity indices (π) and the population differentiation statistic (*F_ST_*) of three groups of *P. massoniana*.
