# Supplementary material for: Chromosome-level genome assembly of Pinus massoniana provides insights into conifer adaptive evolution
Source: Gigascience. 2025 May 30;14:giaf056. doi: 10.1093/gigascience/giaf056 (PMC12122422; doi:10.1093/gigascience/giaf056)
Supplement: giaf056_GIGA-D-24-00472_Revision_1 [file giaf056_giga-d-24-00472_revision_1.pdf]

GigaScience

Chromosome-Level Genome Assembly of Pinus massoniana provides insights into Conifer Adaptive Evolution

--Manuscript Draft--

**Manuscript Number:**	GIGA-D-24-00472R1
**Full Title:**	Chromosome-Level Genome Assembly of Pinus massoniana provides insights into Conifer Adaptive Evolution
**Article Type:**	Research
**Funding Information:**	
**Abstract:**	Pinus massoniana, a conifer of significant economic and ecological value in China, is renowned for its wide adaptability and oleoresin production. We sequenced and assembled the chromosomal-level P. massoniana genome, revealing 80,366 protein-coding genes and significant gene family expansions associated with stress response and plant-pathogen interactions. Long-intron genes, which are predominantly presented in low-copy gene families, are strongly linked to the recent LTR burst in the Pinus genome. By reanalyzing population transcriptomic data, we identified genetic markers linked to oleoresin synthesis, including those within the CYP450 and TPS gene families. The results suggest that the genes of resin terpene biosynthesis pathway can be activated in several cell types and the oleoresin yield may depend on the rate-limiting enzymes. Using multi-omics algorithm, we identified several regulatory factors, including PmMYB4 and PmbZIP2, that interact with TPS and CYP450 genes, potentially playing a role in oleoresin production. This was further validated through molecular genetics analyses. We observed signatures of adaptive evolution in dispersed duplicates and horizontal gene transfer events that have contributed to the species adaptation. This study provides insights for further research into the evolutionary biology of conifers and lays the groundwork for genomic-assisted breeding and sustainable management of Masson pine.
**Corresponding Author:**	jianbo xie<br>Beijing Forestry University College of Biological Sciences and Biotechnology<br>Beijing, CHINA
**Corresponding Author Secondary Information:**	
**Corresponding Author's Institution:**	Beijing Forestry University College of Biological Sciences and Biotechnology
**Corresponding Author's Secondary Institution:**	
**First Author:**	Hu Chen
**First Author Secondary Information:**	
**Order of Authors:**	Hu Chen
	Xinghu Qin
	Yinghao Chen
	Haoyu Zhang
	Yuanheng Feng
	Jianhui Tan
	Xinhua Chen
	La Hu
	Junkang Xie
	jianbo xie
	Zhangqi Yang

Powered by Editorial Manager® and ProduXion Manager® from Aries Systems Corporation

**Order of Authors Secondary Information:**	
**Response to Reviewers:**	Cover letter for “Chromosome-Level Genome Assembly of Pinus massoniana Provides Insights into Conifer Adaptive Evolution”<br><br>Dear Editor and Reviewers,<br><br>Thank you for your valuable feedback on our manuscript, "Chromosome-Level Genome Assembly of Pinus massoniana Provides Insights into Conifer Adaptive Evolution". We appreciate the time and effort you have invested in reviewing our work and providing constructive comments. Below, we address each of your concerns and outline the revisions made to improve the manuscript.<br><br>GIGA-D-24-00472<br>Chromosome-Level Genome Assembly of Pinus massoniana provides insights into Conifer Adaptive Evolution<br>Hu Chen; Xinghu Qin; Haoyu Zhang; Yinghao Chen; Yuangheng Feng; Jianhui Tan; Xinhua Chen; La Hu; Junkang Xie; jianbo xie; Zhangqi Yang<br>GigaScience<br><br>Dear Prof. Xie,<br>Your manuscript "Chromosome-Level Genome Assembly of Pinus massoniana provides insights into Conifer Adaptive Evolution" (GIGA-D-24-00472) has been assessed by our reviewers. Based on these reports, and my own assessment as Editor, I am pleased to inform you that it is potentially acceptable for publication in GigaScience, once you have carried out some essential revisions suggested by our reviewers.<br><br>Their reports, together with any other comments, are below. Please also take a moment to check our website at https://www.editorialmanager.com/giga/ for any additional comments that were saved as attachments.<br><br>Once you have made the necessary corrections, please submit a revised manuscript online at:<br><br>https://www.editorialmanager.com/giga/<br><br>If you have forgotten your username or password please use the "Send Login Details" link to get your login information. For security reasons, your password will be reset.<br><br>Please include a point-by-point within the 'Response to Reviewers' box in the submission system. Please ensure you describe additional experiments that were carried out and include a detailed rebuttal of any criticisms or requested revisions that you disagreed with. Please also ensure that your revised manuscript conforms to the journal style, which can be found in the Instructions for Authors on the journal homepage. If the data and code has been modified in the revision process please be sure to update the public versions of this too.<br><br>The due date for submitting the revised version of your article is 17 Apr 2025.<br><br>We look forward to receiving your revised manuscript soon.<br><br>Best wishes,<br><br>Qing Lan<br>GigaScience<br>www.gigasciencejournal.com<br><br><br>Reviewer reports:<br>Reviewer #1: The manuscript describes a Pinus massoniana genome sequence assembly and various aspects of genome analyses. This study provides insights for further research into the evolutionary biology of conifers and lays the groundwork for genomic-assisted breeding and sustainable management of Masson pine. Overall, this study provides the useful reference genome and potential useful SNP information.

	Nevertheless, I have a few suggestions to further improve the manuscript.<br><br>R: We thank Reviewer 1 for pointing out areas where the text could be clarified. In the revised manuscript, we have made the following improvements:<br><br>We have rewritten sections where the language was ambiguous, ensuring that the technical terms and arguments are presented more clearly. For instance, we have rephrased discussions on genome assembly and comparative genomics to enhance readability.<br>We have incorporated additional concrete examples to support our arguments. For example, we have changed specific figures that detail the genome assembly statistics and comparative analysis results, respectively. These additions provide a stronger foundation for our conclusions.<br><br>1,"As a result, we identified several key modules of CYP450 and TPS, genes associated with the biosynthesis of oleoresin, that displayed significant co-expression patterns". The authors need to be specific. Which P450s and which TPS? These are large gene families. For the P450s the very large majority of genes has no role in oleoresin biosynthesis. This part of the study needs to include knowledge of function, and should not be based solely on patterns of gene expression and association, which as pointed out above, already appears to be flawed.<br>R: Thank you for pointing this out. Specific CYP450 and TPS genes associated with oleoresin biosynthesis were identified and detailed in Table S11 of the supplemental materials. For instance, key CYP450 genes include CYP720B4 and CYP720B6, while critical TPS genes such as TPS1 and TPS6 were highlighted. These were identified based on co-expression patterns and SNP association studies, providing a functional basis beyond expression alone. We have updated the manuscript and add these specific genes into the manuscript (Line 581-582).<br><br>Line 70: Expand on the rationale for selecting the 6-year-old Pinus massoniana tree as the sample source.<br>R: The 6-year-old Pinus massoniana tree was selected for its representation of the mature growth phase when oleoresin production peaks. This stage ensures a robust dataset for investigating stress responses and metabolic activities relevant to oleoresin biosynthesis. We have added this information into the Methods Section (line 101-103).<br><br>Line 188: The enrichment analysis for stress-related gene families would be more compelling if the statistical thresholds and enrichment scores were explicitly stated in the text.<br>R: The enrichment analysis was performed using the clusterProfiler package with statistical thresholds set at a p-value < 0.05 and q-value < 0.05 for GO and KEGG analyses. Enrichment scores and detailed results are available in Supplemental Table S7. We have stated in the text accordingly.<br><br>Line 235: Clarify the specific methodology used for detecting horizontal gene transfer (HGT) events in the second step if there are specific criteria for identifying HGT. This will strengthen the reproducibility of the study.<br>R: Horizontal gene transfer (HGT) events were identified using a two-step workflow. Step one involved BLAST searches to calculate the Alien Index (AI) and outgroup percentage (outg_pct) as outlined in the methods. Step two confirmed candidate genes through phylogenetic analysis using MAFFT with 'auto' option, using trimAl with 'automated1' option, and IQ-TREE with 1,000 ultrafast bootstrapping replicates to construct ML tree. All the criteria and details have been clarified in the manuscript (line 273-290).<br><br>Line 362, Some analyses of "Gene family expansion and contraction" was overlapped with the part "Phylogenetic Analysis and Gene Family Expansion". Not sure it is in the right position, or the title need to be modified.<br>R: Thank you for pointing this out. We merged the paragraphs "Gene Family Expansion and Contraction" and "Phylogenetic Analysis" and the overlap has been addressed.<br><br>Line 421: Are there known interactions for AP2/ERF genes with other stress-related pathways?

Powered by Editorial Manager® and ProduXion Manager® from Aries Systems Corporation

R: Known interactions between AP2/ERF genes and other stress-related pathways are well-documented in literature and supported by expression data under abiotic stresses in Figure S12. These interactions are critical in modulating responses to drought and pathogen resistance, for instance, Guo LY, Wang S, Nie YQ, Shen YR, Ye XX and Wu WW. Convergent evolution of AP2/ERF III and IX subfamilies through recurrent polyploidization and tandem duplication during eudicot adaptation to paleoenvironmental changes. Plant Commun. 2022;3 6:15. doi:Artn 100420. We have also cited these references in our revised manuscript.

Line 462, "provided the experimental evidence for the direct interaction between PmMYB4 and the promoter regions of CYP450 genes": PmMYB4 should not be italicized (here it is a protein).
R: Corrected. Protein names such as PmMYB4 are no longer italicized.

Line 481-487: How many SNP were used, how many significant SNP loci were identified in the GWAS, and how many known genes were successfully annotated to the genome. Do these genes have some overlaps with these genes identified in other analyses, for example WGD or HGT, or these genes provide novel insights?
R: A total of 503,296 SNPs were analyzed, identifying 6,064 SNPs significantly associated with oleoresin synthesis. Among them, 1,056 SNPs were annotated to 10 known genes, some overlapping with WGD and HGT candidate genes, offering novel insights into oleoresin biosynthesis pathways. We have mentioned and stated this in the main text.

Line 487, Please confirm the view of "The GTP diphosphokinase CRSH protein, localized in the chloroplast, which contains EF-hand 1, EF-hand 5, HD 4, and RelA SpoT domains, is crucial for energy metabolism and could be a key regulator in the biosynthesis of oleoresin."
R: The role of the GTP diphosphokinase CRSH protein is validated through its localization in chloroplasts and its functional domains. This aligns with its potential as a key regulator in oleoresin biosynthesis. Experimental details are described in the discussion section.

Line 490, "one set of important SNPs related to oleoresin biosynthesis are the terpene synthase family": change "are" to "is"
R: Corrected. The sentence now reads, "One set of important SNPs related to oleoresin biosynthesis is the terpene synthase family."(line 559-560).

Line 522: "the transcription factor PmMYB4 was identified as a key regulatory component": "PmMYB3" should not be italicized.
R: Corrected. Italicization of protein names has been revised throughout the manuscript.
Line 604: The conclusion briefly mentions the potential applications of the findings but does not elaborate on specific genomic-assisted breeding strategies. Expanding a few words or sentences would make the discussion more actionable for applied research.
R: The conclusion now elaborates on specific applications, including the development of high-yield oleoresin genotypes and strategies to enhance stress resilience using identified SNP markers and transcription factors like PmMYB4. We have included in these in the discussion (line 687-691).

Reviewer #2: With the advancement of sequencing technologies, plant genome research has entered an era of higher precision. Recent publications on the high-quality genomes of species within the Pinus genus have provided valuable resources for researchers to further explore the development and conservation of this economically important group. This study performed high-quality genome assembly and annotation of Pinus massoniana using PacBio long-read sequencing and Hi-C data. Additionally, the analyses of Pinus massoniana's genomic features and evolution, and the mechanism of oleoresin biosynthesis offer new insights into the biology of the Pinus genus. However, regarding the discussion of the adaptation of Pinus massoniana, the current analysis lacks sufficient support. Incorporating an analysis of the correlation between the genomic data, the environmental characteristics of its distribution, and relevant adaptive traits would enhance the persuasiveness and

Powered by Editorial Manager® and ProduXion Manager® from Aries Systems Corporation

scientific rigor of the research.

R: Thanks very much for Reviewer 2 for providing invaluable comments, which could greatly improve the quality of our manuscript. We have read and revised the manuscript according to the reviewer's comments. Below, we reply to the reviewer's comments point-by-point.

I have a few minor suggestions：
Lines 28-29: Understanding the genetic underpinnings of P. massoniana's adaptation to diverse environments is of paramount importance, particularly concerning stress responses and oleoresin biosynthesis. The logic is too abrupt; add causal conjunctions to link it with the preceding discussion smoothly.
Response: Thank you for the suggestion. We have revised the sentence to enhance clarity and flow (line 27-30).

Lines 32-33: However, existing studies predominantly focus on a limited array of conifer species such as Picea abies, Pinus taeda, and Picea glauca. What are the limitations of these studies? What advantages does P. massoniana in this study have in comparison?
Response: The limitations of prior studies and the advantages of P. massoniana are discussed following that sentence in Introduction. We have expanded on it in the revised manuscript (line 36-47).

Lines 51-52: In this context, the sequencing of the P. massoniana genome heralds a new era in comprehending conifer biology. Why? The reasoning is missing.
R: We have added the reasoning for sequencing P. massoniana's genome:

Revised Text: "The sequencing of the Pinus massoniana genome represents a significant advancement in conifer biology. This chromosomal-level assembly provides crucial insights into the molecular mechanisms driving both resin biosynthesis and defense responses within the Pinus genus. By understanding the genetic pathways behind resin production and how conifers defend against pathogens and environmental stress, this research opens new avenues for improving conifer resilience and optimizing the production of resin-based compounds." (line 76-81).

Lines 314-315: Figure 1A-B
Regarding Figure 1B, if there are no methodological innovations, it can be moved to supplementary figures. Consider placing genome quality assessment results instead.
R: We have moved Figure 1B to supplementary Figures and replaced the genome quality assessment results (see Figure 1).
Line 339: Figure 2C
The arrangement of Figure 2 should correspond to the order of results described in the text.
R: We have reorganized the narrative structure of the results to align with the figure (line 362-367; line 382-406; line 412-433). Figure 2C is in line 402, 470.

Lines 359-361: This period of gene family dynamics coincided with critical adaptations in plant evolution, particularly in response to environmental changes.
The evidence is insufficient. Please supplement the analysis with results correlating adaptive traits related to environmental changes and gene family dynamics.
R：We have re-organized the structure of the paragraph. Thus, this statement has been eliminated (line 423-425).
In terms of gene family related to adaptations, we have added specific sentences to environmental adaptations, such as stress-related gene families (e.g., CYP450, TPS, AP2/ERF) that are linked to the species' resilience in challenging environments. These genes correlate with adaptive traits like pathogen resistance, drought tolerance, and oleoresin production (line 394-396; line 486-488; line 495-496).

Lines 364-366: Our results showed that 1,356 gene families have expanded in the P. massoniana lineage, while 982 gene families have contracted (Figure 3A).
There is a mismatch between the figure and the text.
R: We have corrected the mismatch by updating the figure and text to reflect the correct number of expanded and contracted gene families. The revised text now accurately describes the findings:

Revised Text: "Our analysis revealed 6,434 gene families expanded, while 2,405 gene families contracted (Figure 2A)." (line 392-393).

Lines 378-380: The increased capacity for flavonoid production likely contributes to the species' resilience in diverse and challenging environments.
The results do not show this; avoid speculative discussions in the results section.
R: We have revised the speculative text. The suggestion regarding flavonoid production has been removed from the results section (line 444).

Line 530: Discussion
Please cite specific results as evidence in the discussion section (e.g., Figure X, Table X, etc.).
R: We have updated the discussion section to include specific references to figures and tables where applicable.
Lines 539-540: ...... underscoring the evolutionary strategies that enable P. massoniana to thrive in diverse habitats across Southern China.
It would be beneficial to include supplementary data on habitat distribution and compare it with other groups' habitats.
R: Thank you for your insightful suggestion regarding the inclusion of supplementary data on habitat distribution and comparative analysis with other groups' habitats. We acknowledge the value such information would add to our study. Our research primarily focuses on the genomic analysis of P. massoniana, and provides the first-chromosome reference genome for future genetic studies, and the results from population structure, genes under selection, expended and duplicated genes provide insights for the adaptation of P. massoniana. Compiling comprehensive habitat distribution data and conducting comparative habitat analyses could add information on the evolutionary strategies of P. massoniana, however, this also would distract the main focus of our study.
We have changed our statement ("deleted a diverse habitat"). We appreciate your understanding and hope that the genomic insights presented in our study will serve as a foundation for future research exploring more evolutionary aspects of P. massoniana and related species.

Line 578: The population structure analysis......
Consider presenting the related results in the main text of the article.
R: These related results present in the "Genetic basis of Oleoresin Yield", now we have removed this discussion and changed the related result section to "The Population Genetic Structure and the Genetic Basis of Oleoresin Yield" (line 535, line 648-657).

Line 595: ......possibly linked to significant climatic changes during the Miocene epoch. Can you add relevant climate model change curves?

R: We appreciate your suggestion to include climate model change curves related to the Miocene epoch. We tried hard to make such figures including climate changes (curves) during revision, however, the data we inquired from elsewhere differed due to different model selection. While such data would indeed enrich the discussion, the current scope of our study is focused on the genomic aspects of Pinus massoniana, and integrating detailed paleoclimatic models would require extensive additional research and analysis beyond our present objectives. However, we acknowledge the importance of this perspective and suggest that future studies could explore the relationship between historical climate changes and the evolutionary adaptations of P. massoniana in greater detail. The latest manuscript has cited researches of climate characteristics in Miocene, which could align the context of our findings. (line 663-666).
References:
1.Zachos J, Pagani M, Sloan L, Thomas E and Billups K. Trends, rhythms, and aberrations in global climate 65 Ma to present. Science. 2001;292 5517:686-93. doi:10.1126/science.1059412.
2.Reuter M, Kern A K, Harzhauser M, et al. Global warming and South Indian monsoon rainfall-lessons from the Mid-Miocene[J]. Gondwana Research, 2013, 23(3): 1172-1177.

Lines 599-601: ......supports the notion that genomic redundancy can act as a reservoir for evolutionary innovation, especially in response to biotic and abiotic stressors.
Add relevant references.

	R: We have added references to relevant literature supporting the idea that genomic redundancy can foster evolutionary innovation in response to environmental stressors (line 672).<br>References:<br>1.Almeida-Silva F and Van de Peer Y. Whole-genome Duplications and the Long-term Evolution of Gene Regulatory Networks in Angiosperms. Molecular biology and evolution. 2023;40 7 doi:10.1093/molbev/msad141.<br>2.Panchy N, Lehti-Shiu M and Shiu SH. Evolution of Gene Duplication in Plants. Plant Physiol. 2016;171 4:2294-316. doi:10.1104/pp.16.00523.<br><br>Lines 618-619: ......P. massoniana has benefited from genetic material that enhances its adaptability to environmental stresses.<br>All organisms face some degree of environmental stress; specify the types of environmental stresses of P. massoniana.<br>R: We have specified the types of environmental stresses to which P. massoniana is adapted, including drought, pest attacks, and pathogens (line 696).<br><br>Line 637: ......emphasizing its adaptive strategies.....<br>Consider briefly discussing these adaptive strategies in the background section.<br>R: We have introduced a brief discussion on adaptive strategies, including drought resistance and oleoresin biosynthesis, in the background section to provide context for the genomic analyses (line 62-68) .<br><br><br>We are grateful for the reviewers' and editor's constructive feedback, which has significantly improved the manuscript. We believe the revisions address all the concerns raised and enhance the clarity, completeness, and scientific rigor of the study. We are confident that the revised manuscript meets the journal's standards and is now suitable for publication.<br><br>Should further revisions be required, we are more than willing to make additional adjustments.<br><br>Thank you again for your guidance and support.<br><br>Sincerely,<br><br>Prof Jianbo Xie
**Additional Information:**	
**Question**	**Response**
Are you submitting this manuscript to a special series or article collection?	No
**Experimental design and statistics**<br><br>Full details of the experimental design and statistical methods used should be given in the Methods section, as detailed in our Minimum Standards Reporting Checklist. Information essential to interpreting the data presented should be made available in the figure legends.<br><br>Have you included all the information requested in your manuscript?	Yes

**Resources**<br><br>A description of all resources used, including antibodies, cell lines, animals and software tools, with enough information to allow them to be uniquely identified, should be included in the Methods section. Authors are strongly encouraged to cite Research Resource Identifiers (RRIDs) for antibodies, model organisms and tools, where possible.<br><br>Have you included the information requested as detailed in our Minimum Standards Reporting Checklist?	Yes
**Availability of data and materials**<br><br>All datasets and code on which the conclusions of the paper rely must be either included in your submission or deposited in publicly available repositories (where available and ethically appropriate), referencing such data using a unique identifier in the references and in the "Availability of Data and Materials" section of your manuscript.<br><br>Have you have met the above requirement as detailed in our Minimum Standards Reporting Checklist?	Yes
GigaScience has policies and guidelines in place for the use of generative AI-writing tools such as ChatGPT. If you have used such writing tools to assist with writing the manuscript this must be declared and cited in the text. Authors should not list AI-writing tools and other AI-assisted technologies as an author or co-author and should acknowledge that they are fully responsible for text generated or refined by AI-writing tools.<p><br><br>A summary of use (particularly in the introduction or among methods) needs to be included at the end of the paper, and the outputs should also be included as a supplementary file hosted in GigaDB or other open repositories. Please <a	No

href=https://academic.oup.com/gigascience/pages/editorial_policies_and_reporting_standards target="_new" > read our guidelines for more information. <p><br><br>By submitting to GigaScience, you are aware of the journal's AI-writing tools policy, and if you have declared use of such tools below, you have acknowledged this where appropriate in your manuscript and have made a summary of use and outputs available. <p><br><b>AI-assisted writing tools have been used in the preparation of this manuscript?	

Manuscript

Click here to access/download;Manuscript;manuscript_04182025.docx

Chromosome-Level Genome Assembly of *Pinus massoniana* provides insights into Conifer Adaptive Evolution

Hu Chen[1,2,3†], Xinghu Qin[4,5,7,8†], Yinghao Chen[1,2,3†], Haoyu Zhang[4,5,6], Yuanheng Feng[1,2,3], Jianhui Tan[1,2,3], Xinhua Chen[1,2,3], La Hu[1,2,3], Junkang Xie[1,2,3], Jianbo Xie[4,5,6*], Zhangqi Yang[1,2,3*]

[1]Key Laboratory of National Forestry and Grassland Administration on Cultivation of Fast-Growing Timber in Central South China, Guangxi Forestry Research Institute

[2]Guangxi Key Laboratory of Superior Timber Trees Resource Cultivation, Guangxi Forestry Research Institute

[3]Guangxi Key Laboratory of Special Non-wood Forests Cultivation and Utilization, Guangxi Forestry Research Institute

[4]State Key Laboratory of Tree Genetics and Breeding, College of Biological Sciences and Technology, Beijing Forestry University, Beijing 100083, China

[5]National Engineering Research Center of Tree Breeding and Ecological Restoration, College of Biological Sciences and Technology, Beijing Forestry University, Beijing 100083, China

[6]The Tree and Ornamental Plant Breeding and Biotechnology Laboratory of National Forestry and Grassland Administration, Beijing Forestry University, Beijing 100083, China

[7] School of Ecology and Nature Conservation, Beijing Forestry University & The Capital Biodiversity Conservation Institute, Beijing 100083, China

[8] China (BJFU) -UK (St Andrews) International Joint Machine Learning Laboratory for Biodiversity Research, Beijing Forestry University, Beijing 100083, China.

[†]These authors contributed equally to this work.

To whom correspondence should be addressed: Zhangqi Yang: yangzhangqi@163.com; Correspondence may also be addressed to Jianbo Xie, Email: jbxie@bjfu.edu.cn; Tel: +86-10-62336007; Fax: +86-10-62336164.

ORCID iDs: Hu Chen [0000-0002-5691-6259]; Xinghu Qin [0000-0003-2351-3610]; Yinghao Chen [0000-0003-2649-1744]; Haoyu Zhang; Yuanheng Feng; Jianhui Tan; Xinhua Chen [0000-0002-4930-7375]; La Hu [0000-0003-1701-302X]; Junkang Xie [0009-0003-2695-2958]; Jianbo Xie [0000-0002-8650-7675]; Zhangqi Yang.

Abstract

Pinus massoniana, a conifer of significant economic and ecological value in China, is renowned for its wide adaptability and oleoresin production. We sequenced and assembled the chromosomal-level *P. massoniana* genome, revealing 80,366 protein-coding genes and significant gene family expansions associated with stress response and plant-pathogen interactions. Long-intron genes, which are predominantly presented in low-copy gene families, are strongly linked to the recent LTR burst in the *Pinus* genome. By reanalyzing population transcriptomic data, we identified genetic markers linked to oleoresin synthesis, including those within the *CYP450* and *TPS* gene families. The results suggest that the genes of resin terpene biosynthesis pathway can be activated in several cell types and the oleoresin yield may depend on the rate-limiting enzymes. Using multi-omics algorithm, we identified several regulatory factors, including PmMYB4 and PmbZIP2, that interact with *TPS* and *CYP450* genes, potentially playing a role in oleoresin production. This was further validated through molecular genetics analyses. We observed signatures of adaptive evolution in dispersed duplicates and horizontal gene transfer events that have contributed to the species adaptation. This study provides insights for further research into the evolutionary biology of conifers and lays the groundwork for genomic-assisted breeding and sustainable management of Masson pine.

Keywords: *Pinus massoniana*, Conifer evolution, Genomic assembly, Repeat sequences, Gene family expansion, Oleoresin biosynthesis, Population genetics

Introduction

Conifers, an ancient lineage of seed plants, play a crucial role in terrestrial ecosystems globally. Among them, *Pinus massoniana* (NCBI:txid88730) stands out as a dominant species in Southern China, valued for its timber, pulpwood, and especially its rich oleoresin production [1], which significantly contributes to the national economy. Oleoresin not only contributes to the national economy but also serves critical ecological functions, acting as a defensive mechanism against pests and pathogens [2]. Given its ecological and economic significance, understanding the genetic underpinnings of *P. massoniana*'s adaptation to diverse environments becomes essential, particularly in relation to stress responses and oleoresin biosynthesis.

Recent strides in conifer genomics have shed light on the evolutionary adaptations of these species, unraveling unique characteristics and mechanisms that set them apart from other plant lineages [3-6]. However, existing studies predominantly focuses on a limited array of conifer species such as *Picea abies, Pinus taeda*, and *Picea glauca* [7-9], which have provided valuable insights but also face several limitations. These genomes are typically large, complex, and rich in repetitive sequences, such as transposable elements (TEs), which complicate genome assembly and annotation [9]. Early sequencing efforts, often relying on short-read technologies, resulted in fragmented and incomplete genomes, requiring more advanced methods like long-read sequencing and Hi-C for improved assembly. For example, the *Picea abies* genome was initially assembled using short-read sequencing, resulting in a fragmented assembly with large gaps [3]. Even with advances in sequencing technologies such as long-read sequencing (e.g., PacBio), gaps remain in functional annotation, with limited studies on specific traits like oleoresin biosynthesis or complex metabolic pathways. Moreover, many conifer genome

studies, such as those on *Pinus taeda*, have focused primarily on general genomic features or stress-resistance traits like cold tolerance [10], leaving other critical traits, such as the biosynthesis of secondary metabolites (e.g., oleoresin), underexplored. In contrast, *P. massoniana*'s broad ecological range and oleoresin biosynthesis make it an ideal model for studying both evolutionary biology and functional genomics, particularly for understanding stress resistance and metabolic pathways unique to pine species.

Pinus massoniana has developed several adaptive strategies to thrive in diverse and challenging environments. Under low light and drought conditions, it modulates the production of secondary metabolites, such as flavonoids and terpenoids, which play crucial roles in defense mechanisms and stress tolerance [11]. Additionally, *P. massoniana* forms symbiotic relationships with ectomycorrhizal fungi like *Suillus luteus*, enhancing its nutrient absorption and resistance to soil-borne pathogens [12]. These adaptations collectively enable *P. massoniana* to withstand various environmental stresses, contributing to its ecological success and economic importance in subtropical regions.

The exceptional ability of *Pinus spp*., including *P. massoniana*, to synthesize oleoresin—a complex blend of turpentine and rosin crucial for defense against biotic and abiotic stresses—sets them apart [13]. This oleoresin not only ensures the tree's survival but also holds substantial value for various industries, serving as a raw material for chemical and food sectors and a key precursor in biofuel production [14, 15]. Notably, *P. massoniana* accounts for 70% of China's total oleoresin yield, underscoring its significance to the nat ional economy [1]. The evolutionary origins of this biosynthetic machinery in conifers, a trait uncommon in most flowering plants, pose another intriguing question that our study aims to address. The sequencing of the *Pinus massoniana* genome represents a

significant advancement in conifer biology. This chromosomal-level assembly provides crucial insights into the molecular mechanisms driving both resin biosynthesis and defense responses within the *Pinus* genus. By understanding the genetic pathways behind resin production and how conifers defend against pathogens and environmental stress, this research opens new avenues for improving conifer resilience and optimizing the production of resin-based compounds. Although the large genome size in conifers presents challenges for genome-wide analyses and sequencing efforts, the sequencing and analysis of these large genomes provide valuable insights into their evolution, adaptation, and the unique features that allow them to dominate various ecosystems around the world. Additionally, the study of *P. massoniana* genome can offer new avenues for forestry and breeding due to their economic and ecological importance.

In this study, we present the first chromosome-level assembly of the *P. massoniana* genome, leveraging the power of next-generation sequencing technologies and advanced bioinformatics tools. We also performed genomic analyses based on genomic sequences, large-scale RNA-seq data of 156 biological samples and 204 transcriptomic data of wild accessions. Our analysis delves into the structural composition of the genome, the expansion of gene families, the evolution of conifer species, the key genes associated with adaptative traits as well as the key factors that involved in the regulating of resin terpene biosynthesis. The results provide insight into the genomic features and molecular mechanisms related to the resin terpene biosynthesis mechanism of *P. massoniana*. Our study paves the way for future studies on the evolutionary biology of conifers and has practical implications for the sustainable management and utilization of these ecologically and economically important species.

Materials and Methods

Plant materials

For our genomic study, we procured samples from a single, 6-year-old *Pinus massoniana* tree of the Songyun variety (Figure 1A). This 6-year-old *P. massoniana* tree was selected for its representation of the mature growth phase when oleoresin production peaks, ensuring a robust dataset for investigating stress responses and metabolic activities relevant to oleoresin biosynthesis. This variety has undergone official audit by the Office for the Protection of New Varieties of Plants under the State Forestry Administration of the People's Republic of China. The tree is situated in Nanning, China, with precise geographic coordinates of 23°10′N latitude and 107°59′E longitude. To obtain a representative sample, we collected approximately 50 grams of the current year's mature needles from the midsections of the tree's cardinal aspects—north, south, east, and west—and pooled them to ensure a uniform sample for analysis (Figure 1A).

DNA extraction

The genomic DNA (gDNA) was isolated from the pooled needle samples using the DNeasy Plant Mini Kit from QIAGEN, following the provider's guidelines optimized for library construction and sequencing. The yield and purity of the extracted gDNA were evaluated using a Nanodrop One spectrophotometer (NanoDrop Technologies, Wilmington, US) and further confirmed by assessing the integrity with an Agilent 4200 Bioanalyzer (Agilent Technologies, Palo Alto, California).

Genome survey and genome size estimation

Genome survey

To estimate the genome size, heterozygosity and repeat content, we used Jellyfish (RRID:SCR_005491) v2.1.4 [16] with the parameters of '-t 10 -C -m 41 -s 22G' to generate a 41 *K*-mer frequency distribution. Genome size (G) was estimated by $G=k_{num}/k_{depth}$, where the k_{num} represents the total number of *k*-mers, and the k_{depth} denotes the *k*-mer depth of the peak frequency of *k*-mer distribution. Depth of *K*-mer = 1 is considered as an error, and this error rate was used to calculate and correct the genome size. Then, the genome size, heterozygosity and repeat content were estimated by the genomeScope [17] from the short sequencing data using Jellyfish v2.1.4 with the parameters of '-t 10 -C -m 41 -s 22G'. We also estimated the genome size by using the flow cytometry experiment, nuclei were released by chopping the young needles and analyzed with the Beckman-Coulter Moflo XDP Cell Sorter (RRID:SCR_019665) according to the manufacturer's instructions.

Short-read sequencing

For the short-read sequencing, the qualified genomic DNA was randomly cut into fragments of approximately 350 bp in length. The size was further verified by using the Agilent 2100 Bioanalyzer (RRID:SCR_018043; Agilent Technologies, Santa Clara, USA), followed by end repair, polyadenylation, adapter ligation, target fragment selection, and PCR amplification using the Nextera XT DNA Library Prep Kit. Then, the Qubit 2.0 Fluorometer (Life Technologies, Carlabad, USA) and Agilent 2100 (RRID:SCR_018043; Agilent Technologies, Santa Clara, USA) were used to check the preliminary quantitative and insert size of library. Sequencing was performed on BGI BGISEQ-500

platform (RRID:SCR_017979).

Pacific Biosciences Technologies (PacBio), and Hi-C sequencing

PacBio library construction and sequencing

PacBio libraries were constructed with a SMRTbell Template Prep Kit 1.0 (Pacific Biosciences) and the SMARTbell Damage Repair Kit (Pacific Biosciences). Sequencing was performed on the PacBio Sequel platform. In brief, the genomic DNA was sheared into fragments (~20 kb) using a Covaris g-Tube (Covaris). The templates were size-selected using BluePippin (Sage Science, MA, USA) to enrich large DNA fragments (>15 kb), followed by primer annealing and the binding of SMRT bell templates to polymerases with the Sequel Binding Kit.

Hi-C library construction and sequencing

For Hi-C sequencing, fresh needle samples were fixed in 1% formaldehyde to maintain the 3D structure of genome. The genomic DNA was extracted and digested with restriction endonuclease MboI. The sticky ends of the digested fragments were biotinylated, diluted and ligated randomly. The ligated DNA was sheared into 300-600 bp fragments, blunt-end repaired and purified. The libraries were sequenced on the BGI BGISEQ-500 platform and 150 bp paired-end reads were generated.

Genome assembly and chromosome anchoring

A hybrid strategy was used to assemble the genome sequence. The PacBio reads were used for initial contig assembly in Smartdenovo (RRID:SCR_017622) v. 2.3.1 software [18]. Next, the assembled contigs were polished three times using Nextpolish (RRID:SCR_025232) v. 1.3.1 software [19] based

on the short reads. Subsequently, Hi-C sequencing data were used to anchor the draft genome with Juicer (RRID:SCR_017226) v. 1.6. To estimate genome size and heterozygosity, Jellyfish v. 2.1.4 [16] and Genomescope (RRID:SCR_017014) [17] software were used (kmer=41).

Genome evaluation

We used short genomic sequencing data and the Iso-seq full-length transcripts to evaluate the quality of assembly. The quality-controlled short genomic reads were mapped to the genome assembly using BWA-MEM (RRID:SCR_010910) [20], and information on the mapping ratio was collected. Evaluation by Iso-seq was performed in two steps. First, Iso-seq data were assembled into high-quality, full-length transcripts using SMRT-Analysis (RRID:SCR_002942) v.2.3. These full-length transcripts were then aligned to the genome using BLAT (RRID:SCR_011919) [21] to evaluate the structural accuracy of the assembly. In addition, the Benchmarking Universal Single-Copy Orthologs (RRID:SCR_015008) (BUSCO, v4.1.4) [22] with embryophyta_odb10 and eukaryota_odb10 database was used to check the assembly quality and the gene annotation with genome and protein modes, respectively.

Gene prediction

The hybrid approaches were used to predict protein-coding genes: homology-based search, *de novo* gene prediction and RNA sequencing-aided annotation. (1) The assembled genome sequence was used for homology-based prediction using GeMoMa (RRID:SCR_017646) v1.8 [23] with default parameter based on nine homologous species (*Amborell trichopoda*, *Oryza sativa*, *P. tabuliformis*, *Pseudotsuga menziesii*, *Arabidopsis thaliana*, *Cycas panzhihuaensis*, *P. lambertiana*, and *Ginkgo biloba*). (2) SNAP

(RRID:SCR_007936) [24] and AUGUSTUS (RRID:SCR_008417) v3.4.0 [25] were used for ab initio gene predictions. (3) To improve gene prediction, we downloaded all the public available transcriptome samples from public database. Then, the NGS transcriptome data was further assembled by Trinity, and the assembled transcripts were further processed using Tansdecoder v5.5.0, to obtain putative protein sequences. We used GMAP (RRID:SCR_008992) v2018-05-30 [26] to align the reads to the assemble genome and then used Transdecoder v5.5.0 to predict ORF in the transcripts to define putative protein sequences for Iso-seq data. Finally, all acquired results were combined and revised using EVM (RRID:SCR_008992) v1.1 [27] and Maker (RRID:SCR_005309) v3.01.03 [28]. The completeness of *P. massoniana* genome sequence was estimated using BUSCO v5.0 software [22].

Gene family expansion and contraction

Based on the phylogenetic tree, gene-family expansion and the contraction of orthologous gene families were inferred using CAFÉ (RRID:SCR_005983) v.4.2. A random birth and death process was used to model gene gain and loss along each lineage in the phylogenetic tree. To make inferences over a whole phylogeny, a probabilistic graphical model was used to estimate the probability of transitions in gene family size from parent to child nodes [29] in the phylogenetic trees of *P. massoniana* and 12 other *Pinus* species.

Analysis of synteny between *Pinus massoniana* and *Pinus tabuliformis*

We used JCVI (RRID:SCR_017650) v. 1.1.14 and MCscan [30] to identify syntenic gene pairs and blocks between *P. massoniana* and *P. tabuliformis.* The coding sequence (CDS) and genome annotation gff3 files of the two species were the input data and we used ‘jcvi.compara.catalog ortholog’

with default parameters to identify syntenic blocks for each pair. Next, 'jcvi.compara.synteny screen' with the parameters --minspan = 30 --simple was used to filter syntenic blocks.

Repeat annotation and LTR insertion time estimation

Repetitive and transposable elements in the *P. massoniana* genome were identified by RepeatMasker (RRID:SCR_012954) v.4.0.7 [31] using denovo libraries constructed by RepeatModeler (RRID:SCR_015027) [32]. And intact LTRs were identified by LTR_FINDER (RRID:SCR_015247) v.1.0.6 [33] with default parameters. Then, all LTR pairs were aligned by using MUSCLE (RRID:SCR_011812), and the nucleotide distance (K) between them was estimated by using distmat programme in the EMBOSS (RRID:SCR_008493) package. The insertion time was calculated as T = K/2r, where the rate of nucleotide substitution (r) used for gymnosperm species was 2.2×10^{-9}.

Gene duplication analysis

Protein sequences were aligned all-*versus*-all using BLAST (RRID:SCR_004870) (v.2.2.28; -e $1e^{-10}$; -max_target_seqs 5). Next, the all-*versus*-all BLAST results and the gff3 files were used as input data for DupGen_finder [34] software; we used the default parameters to identify different modes of duplicated gene pairs. Syntenic regions with collinearity of paralog pairs were identified using MCScanX (RRID:SCR_022067) [35]. We analyzed the distribution of synonymous substitutions per site (*Ks*) for each paralog to evaluate recent whole-genome duplication (WGD) in *P. massoniana.* ParaAT v.2.0 [36] with the default parameters was used to construct multiple protein-coding DNA alignments. KaKs_Calculator (RRID:SCR_022068) v.2.0 [37] with the default parameters was used to calculate the Ks value for each paralog pair.

RNA sequencing data analysis

The raw RNA-seq data of *P. massoniana* were quality-filtered using fastp (RRID:SCR_016962) software with default parameters [38]. Then, clean reads were mapped to *P. massoniana* using Hisat2 (RRID:SCR_015530) v2.0.9 [39] with the parameter --dta -x -p to generate read alignments for each sample. Gene expression levels were normalized using the number of transcripts per kilobase million reads by the StringTie software (RRID:SCR_016323) (v.1.3.5) with default settings [40].

Functional enrichment analysis

To perform functional enrichment analysis, Gene ontology terms and KEGG pathways were assigned to the genes using eggNOG-mapper (RRID:SCR_021165) v2 [41] with default parameters. R package clusterProfiler (RRID:SCR_016884) v3.0.4 [42] were used to perform GO and KEGG enrichment analysis of the expanded genes.

Phylogenetic and domain analyses of TPS and CYP450 proteins

MEME suite (RRID:SCR_001783) [43] with default parameters was employed to identify the conserved motifs of the TPS and CYP450 proteins. To construct phylogenetic tree, multiple alignments were carried out using Muscle v3.6 [44], and iqtree2 (RRID:SCR_017254) [45] was then used to create maximum likelihood phylogenetic trees with parameters: -T AUTO -st AA -bb 1000; bootstrap values were obtained by 1,000 bootstrap replicates. Phylogenetic trees were both visualized with iTOL (RRID:SCR_018174).

SNP calling

The transcriptome sequencing data [2] of 204 wild accessions from 10 main distribution regions were reanalyzed for SNP calling. Filtered reads were mapped to the genome sequence, using SOAPaligner (RRID:SCR_005503) (SOAP2, version 2.20) with default options [46] which were used for SNP calling.

Phylogenetic and population genetic analysis

To construct a phylogenetic tree, a dataset comprising 503,296 single nucleotide polymorphisms (SNPs) was employed to generate maximum likelihood (ML) trees using the IQ-TREE (RRID:SCR_017254) v2.2.2.3 software suite [47]. The optimal evolutionary model was selected based on the Bayesian Information Criterion (BIC). The robustness of the resultant ML trees was assessed utilizing the ultrafast bootstrap (UFboot) method, with 1,000 bootstrap replicates to estimate branch support. Visualization of the ML phylogenetic tree was facilitated through the Interactive Tree Of Life (iTOL) v4 online platform [48]. Principal component analysis (PCA) was conducted using PLINK (RRID:SCR_001757) v1.90p [49] and EIGENSOFT (RRID:SCR_004965) v6.1.4 [50] on the complete set of SNPs, applying filters for minor allele frequency (MAF) greater than 0.05 and allowing less than 10% missing data. The genetic structure of the population was delineated using ADMIXTURE (RRID:SCR_001263) software [51], with the number of presumed ancestral populations (K) ranging from 1 to 10. The most probable number of ancestral genetic clusters was inferred from the cross-validation error curve at the point of minimum K value. Diversity indices (π) and the population differentiation statistic (F_{ST}) were computed using VCFtools (RRID:SCR_001235) v0.1.15 [52] on the filtered SNP dataset. For each sub-population, these values were determined in a sliding window approach with a 20-kb window size and a 5-kb step increment.

Genome-Wide Association Study (GWAS)

In the GWAS, SNP loci with more than 10% missing data across accessions were excluded from analysis. Subsequently, SNPs with a minor allele frequency (MAF) below 5% were subjected to GWAS. The mixed linear model (MLM) in TASSEL (RRID:SCR_012837) v5.2.51 [53] was applied to investigate the association between SNPs and oleoresin yield, adjusting for population structure (Q matrix) and kinship (K matrix) to control for confounding factors. The optimal Q matrix was ascertained using ADMIXTURE (RRID:SCR_001263) software, while the kinship matrix (K) was calculated using the KinshipPlugin within TASSEL. Associations were deemed significant at a P-value threshold of <=1.0E−5, which corrects for multiple testing and reduces the likelihood of Type I errors.

Transcription factors

The TFs were predicted using PlantRegMap according to the 'Family assignment rules' of PlantTFDB (RRID:SCR_003362) [54]. The candidate TFs were further manually filtered by removing those without any conserved protein domains. For the phylogenetic tree construction, TFs of the same family were aligned by the MAFFT (RRID:SCR_011811) v7.520, with "–auto" option and "--maxiterate 1000", and trimmed ambiguously aligned regions using trimAl (RRID:SCR_017334) v1.4 [55] with "-automated1" option. Then, the ML tree were constructed by IQ-TREE v2.2.2.3 [47] with its best-fitting model of amino acid evolution and 1000 ultrafast bootstrapping replicates [56].

Horizontal transfer gene identification

To detect genes that may be acquired from distinct organisms, we employed a robust and conservative phylogeny-based approach, as described in previous study with some modifications [57]. For each

gene’s protein sequence, we used a two-step workflow:

Step 1, we first performed the BLASTP (RRID:SCR_001010) in DIAMOND (RRID:SCR_009457) v2.1.6 [58] search against a custom database (reference protein sequences RefSeq and all proteins from PPGR) with an e-value cutoff of 10^{-10}. HGTfinder v1 [57] were employed to parse the BLAST hits, based on their taxonomic information, into three different lineages (RECIPIENT: Streptophyta; GROUP: Viridiplantae; OUTGROUP: non-Viridiplantae). Five values were calculated: bbhO, represents the BLAST bitscore of the best hit in the OUTGROUP lineage; bbhG, represents the bitscore of the best hit in the GROUP lineage but not in the RECIPIENT lineage; maxB, represents the bitscore of the query to itself. The Alien Index was then calculated as (bbhO / maxB) - (bbhG / maxB), and outg_pct was determined as the percentage of species from the OUTGROUP lineage in the list of the top 1,500 hits that have different taxonomic species names. Genes that met the criteria of having an AI value greater than 0 and an outg_pct higher than 80% were considered to be highly credible HGT genes.

Step 2, we retrieved the 1,500 most similar homologs from the Refseq database (as mentioned above). These homologs were then aligned using MAFFT (RRID:SCR_011811) v7.520 [59], with the 'auto' option. Ambiguously aligned regions were trimmed using trimAl (RRID:SCR_017334) v1.4 [55] with the 'automated1' option. The resulting alignments were used to infer the maximum likelihood (ML) tree using IQ-TREE v2.2.2.3 [47]. The best-fitting model of amino acid evolution was employed, and 1000 ultrafast bootstrapping replicates were performed. To root each ML tree, we utilized the ape and phangorn R packages [60, 61]. The rooted trees were then manually inspected.

Identification of stress-resistance genes

BLAST (RRID:SCR_004870; v.2.2.28; -e $1e^{-10}$) was used to search for homologs in *P. massoniana* using the amino acid sequences of WRKY and AP2 in *Arabidopsis thaliana* [62, 63] as references. To annotate NLRs in *P. massoniana*, we used NLR-ID pipeline [64] and amino acid sequences were aligned to the NB-ARC HMM [65] of the NB-ARC domain using hmmalign with the default parameters (HMMER (RRID:SCR_005305) v.3.0) [66].

Terpenoid biosynthesis pathway

Sequences encoding key enzymes of the terpene biosynthesis pathway [67, 68] were used as references to identify candidate functional homologs in *P. massoniana* using BLASTP v.2.2.28 (-e $1e^{-30}$).

RNA *in situ* hybridization

In situ hybridization with digoxigenin (DIG)-labeled probes was performed as described previously [69]. Stem apex, needle, and root of *Pinus massoniana* were fixed in FAA solution (3.7% formaldehyde, 5% acetic acid, and 5% ethanol). The fixed tissues were dehydrated in a graded ethanol series, embedded in paraffin using a modular automated tissue processor (Leica ASP200S, Germany), and sectioned using a sliding microtome (Leica). After dewaxing and rehydrating, the sections were reacted with proteinase K (Roche, Switzerland), washed in phosphate-buffered saline, and subjected to acetylation. The sections were next pre-hybridized in hybridization buffer for 1 h and incubated with a digoxigenin-labeled riboprobe (Shanghai Gefan Biotechnology Co., Ltd.) for 48 h at 65°C. After hybridization, the sections were rinsed, and the peroxidase reaction was initiated by adding 0.05% 3,3-diaminobenzidine-4 HCl (DAB) and 0.003% H_2O_2.

Genetic transformation and molecular verification of transgenic plants

The coding sequence (CDS) of *PmPGK* was cloned into the pBI121-GFP vector, generating 35S:*PmPGK*-GFP construct. The 35S:*PmPGK*-GFP construct was introduced into poplar 84K transformation as described previously [70]. The presence of transgenic lines was confirmed through PCR analysis, and the expression levels of *PGK* were quantified using RT-qPCR with pine 18S rRNA as an endogenous control.

Dual-luciferase (LUC) assay

The dual-luciferase assay was performed as previously described [71]. The coding sequence of *PmMYB4* was inserted into the pGreen II 62-SK vector to create an effector construct, while the promoter sequence of *PmCYP450.15* (2000 bp) was cloned into the pGreenII 0800-LUC vector to generate a reporter construct. These constructs were co-transfected into *Nicotiana benthamiana* leaves, and the plants were incubated for 48-60 hours [72]. The leaves were sprayed with D-Fluorescein (1 mM) and imaged using an LB983 Night Owl II fluorescence imaging system (Berthold Technologies) to detect luminescence. The relative luminescence intensity was quantified using Image-Pro Plus (RRID:SCR_007369) 6.0 software (Media Cybernetics), with each experiment being conducted in triplicate, including three biological replicates and four technical replicates.

Electrophoretic mobility shift (EMSA) assay

For the EMSA, the full-length *PmMYB4* was expressed in *Escherichia coli* strain Rosetta (DE3) using the pHMGWA expression vector. The His-tagged PmMYB4 protein was induced by 0.05 mM isopropyl-*β*-D-1-thiogalactopyranoside and purified using Ni-NTA Agarose according to the manufacturer's instructions (Qiagen). Biotinylated probes containing the GCC-box or mutated elements were synthesized and used in EMSA with the Light Shift Chemiluminescent EMSA Kit

(Pierce). The DNA-protein binding reactions were performed in a buffer containing 5 mM MgCl2, 50 mM KCl, 10 mM EDTA, 2.5% glycerol, 50 ng/μL Poly (dI-dC), and 0.05% NP-40, followed by separation on a 6.5% nondenaturing polyacrylamide gel and transfer to a nylon membrane for chemiluminescence detection.

Subcellular localization

The subcellular localization of PmMYB4 and PmbZIP2 was determined by cloning its CDS into the pBI121-GFP vector, resulting in a *35S:PmMYB4-GFP* and *35S:PmbZIP2-GFP* construct. This construct was transiently expressed in tobacco protoplasts using established methods [73]. The fluorescence from the transformed protoplasts was visualized using a Zeiss LSM710 confocal microscope after 12-16 hours. The cell membrane was stained with FM4-64 to provide a reference for subcellular localization, with fluorescence detected at excitation/emission maxima of 515/640 nm.

Results

Chromosome-scale sequencing and assembly of the *P. massoniana* genome

We employed a hybrid sequencing approach combining high-coverage short reads, Pacific Biosciences (PacBio) long reads, and Hi-C chromatin interaction data to generate a high-quality chromosome-level genome assembly of *P. massoniana*. To evaluate the accuracy and completeness of the genome assembly, we performed several quality control measures. First, we mapped the short reads back to the assembled genome, achieving a mapping rate of 99.2%, which indicates high accuracy in the assembly (Figure S3A). Second, we used Benchmarking Universal Single-Copy Orthologs (BUSCO) to assess the completeness of the assembly (Figure 1B; Figure S3B). The total length of the assembled genome was approximately 21.91 Gb, comprising 49,639 contigs with an N50 contig length of 4 Mb (Figure

S1-S2). The contigs were further scaffolded using Hi-C data, anchoring them to 12 pseudochromosomes that represent the haploid genome with the lengths of pseudochromosomes ranged from 1.49–2.37 Gb, accounting for 99.89% of the assembled sequences (Figure 1C). The final assembly had an N50 scaffold length of 1.5 Gb, with 96.7% of the genome anchored to the chromosomes (Table S1). The genome contained 95.3% of the conserved single-copy orthologs from the embryophyta dataset, confirming the high quality of the assembly (Table S2).

Repetitive sequences and transposable elements

The *P. massoniana* genome assembly harbored 81.2% (17.79 Gb) repetitive sequence (Figure 1D; Table S3), of which the LTR retrotransposons and LINE elements represented 60.15% and 3.06% of the assembly, respectively. Notably, Gypsy LTR-RTs elements and Copia LTR-RTs elements accounted for 58.38% of the genome (Table S3). The Gypsy LTR-RTs (43.59%) were disproportionately abundant in *P. massoniana* compared to other gymnosperms, a phenomenon potentially attributable to recent species-specific bursts in multiple subfamilies of LTR-RTs (Figure S5). The majority of LTR-RT expansions occurred within the last 5-30 million years (Figure S5), coinciding with the Miocene epoch (5.33-23.03 MYA), a period characterized by global cooling leading up to the ice ages [74-76].

Gene family evolution in *P. massoniana*

In total, we annotated 80,366 protein-coding genes and 132,148 transcripts based on extensive RNA-seq data from 156 biological samples representing various tissues and stress conditions (Table S4). Of these, 89.23% aligned with entries in databases such as Swiss-Prot, KEGG, and Gene Ontology.

To explore the evolutionary trajectory of *P. massoniana*, we conducted a comprehensive phylogenetic analysis using 135 single-copy orthologous genes from 13 plant species, including 12 published genomes and *P. massoniana*. This analysis allowed us to identify and classify 30,558 gene families in the *P. massoniana* genome, comprising 69,152 genes. Of these gene families, 5,215 were found to be conserved across all 13 species, while 9,846 were unique to *P. massoniana*. Additionally, 73,608 genes were identified as having orthologs in the other 12 plant genomes (Figure 2A-B).

Through molecular clock analysis, we estimated that the most recent common ancestor (MRCA) of the Coniferopsida lineage, which includes *P. massoniana*, contained approximately 23,557 gene families. Following the divergence of the Pinaceae and Cupressaceae lineages around 289 million years ago (MYA), the Pinaceae family, including *P. massoniana*, underwent substantial gene family expansion, gaining 5,759 new gene families while losing 1,517 (Figure 2A). In addition, 6,434 gene families have expanded in the *P. massoniana* lineage, while 2,405 gene families have contracted (Figure 2A). Notably, many of the expanded gene families are associated with stress resistance and terpene biosynthesis, including members of the cytochrome P450 (CYP450) and terpene synthase (TPS) families, which play crucial roles in oleoresin production. On the other hand, the average lengths of coding sequences, exons, and introns were 1,007 bp, 1,005 bp, and 12,680.84 bp, respectively. Notably, *P. massoniana* exhibited the longest average intron length compared to other analyzed species (Figure 2A; Table S5).

Our analysis revealed 57,207 duplicated genes, primarily resulting from dispersed duplication (56%) and whole-genome duplication (WGD) events (0.79%) (Figure 2C). Syntenic analysis with *Pinus tabuliformis* revealed 1,471 syntenic blocks, encompassing 25,936 anchor genes (Table S6). The

analysis indicated a low level of synteny, suggesting rapid chromosomal rearrangements within the Pinaceae family. Two distinct peaks in synonymous substitution divergence (Ks) were identified, suggesting that *P. massoniana* has experienced two WGD events approximately 320 MYA and 260 MYA (Figure 2D).

Functional Enrichment of Expanded Gene Families

Functional annotation of the expanded gene families in *P. massoniana* revealed a strong association with stress response mechanisms and plant-pathogen interactions. Pathways related to responses to xenobiotic stimuli, cellular responses to water deprivation, and terpene biosynthesis were significantly overrepresented among the expanded gene families (Figure S4; Table S7). Several pathways related to stress responses, flavonoid biosynthesis, and long-term protection have expanded in *P. massoniana* (Figure S4; Table S7). These results suggest that *P. massoniana* has evolved a robust defense strategy to cope with environmental stressors and pathogen challenges.

Dispersed Gene Duplications and Evolutionary Dynamics

To examine the dynamics of gene duplication in *P. massoniana*, we analyzed the distribution of synonymous substitution rates (Ks) for dispersed gene duplicates across four gymnosperm genomes, including *P. massoniana*, *Ginkgo biloba*, and *Sequoiadendron giganteum* (Figure 2E). The *P. massoniana* genome exhibited a continuous distribution of Ks values, suggesting that dispersed gene duplications have been an ongoing process in this species. In contrast, *G. biloba* and *S. giganteum* displayed two distinct Ks peaks, indicating episodic duplication events.

Interestingly, we observed a strong correlation between transposable element (TE) activity and dispersed gene duplication (DSD) events in the *P. massoniana* genome (Figure S5). The Pinus lineage showed a recent (<10 MYA) proliferation of long terminal repeat retrotransposons (LTR-RTs), which likely contributed to the expansion of dispersed gene duplicates. In contrast, the other gymnosperm species exhibited older LTR-RT bursts, suggesting differing evolutionary trajectories for TE activity and DSD events across gymnosperms.

Intron Length and Gene Expression

Consistent with previous studies, we found that gymnosperms, including *P. massoniana*, possess significantly longer introns compared to angiosperms (Figure 2B). In *P. massoniana*, 18,536 introns exceeded 20 kb in length, with an average intron length of 11.56 kb. Using PacBio long-read sequencing, we validated the authenticity of these long introns, confirming that they are not assembly artifacts but genuine features of the genome (Figure S6).

Our analysis revealed a strong negative correlation between intron length and gene family size, particularly in low-copy-number genes (Figure S7). Low-copy-number genes tend to have longer introns and are more conserved across species, suggesting that intron retention may confer evolutionary advantages, such as enhancing gene regulation and transcriptional efficiency. We also observed that the genome size is positive correlated with intron length (Table S8). Additionally, highly expressed genes in *P. massoniana* were more likely to have longer introns, a pattern consistent with observations in model plant species like *Arabidopsis thaliana* and *Oryza sativa*. The average lengths of genes in gymnosperms were longer than in angiosperms, implying that genome composition is

related to gene length. Also, the gene-coding sequences of gymnosperms and angiosperms were of constant average lengths despite the marked intergenic variation in exon sequence length (Figure S8).

Stress Resistance and Horizontal Gene Transfer

In the genome of *P. massoniana*, we identified 550 NLR genes, with the majority (107 gene pairs) likely resulting from dispersed duplication and 65 pairs from tandem duplication (Figure S9). Our analysis of transcription correlation coefficients revealed that the correlations between gene pairs of genome-wide tandem duplicates (13,088 gene pairs) were significantly lower than WGD gene pairs ($P < 0.001$, permutation test), but correlations between gene pairs derived from tandem duplication were not significantly different from those of dispersed duplication events.

Gymnosperms had the smallest percentage of TFs among all genes (2.74–3.93%; Figure S10). WRKY TFs, which are essential for plant stress tolerance and disease resistance [77], were found to be activated by various treatments and stresses. We detected 31 WRKY TFs, with 27 being expressed in response to a variety of stresses, including prolonged drought, aluminum (Al) stress, and methyl jasmonate (MeJA) treatment (Figures S9C, S11-S12). The expansion of cold-responsive *AP2/ERF* genes, specifically the III group of C-repeat binding factors (*CBFs*), is associated with plant adaptation to paleoenvironmental changes [78, 79]. In *P. massoniana*, we identified 24 *AP*2/*ERF* genes, and transcriptome analysis of 31 samples subjected to various stresses revealed that these genes were actively expressed, with half showing specific expression patterns (Figures S9C, S11-S12).

We identified 689 genes likely acquired through HGT, including 59 from fungi and 109 from bacteria (Table S9). These horizontally acquired genes included several carbohydrate-active enzymes

(CAZymes) involved in cell wall biosynthesis, as well as glycoside hydrolases (GH71 and GH3), which are associated with plant defence and stress responses. Notably, we detected the expansion of ATP-binding cassette (ABC) transporter proteins, which are known to contribute to stress tolerance and environmental adaptation in terrestrial plants. Additionally, several horizontally acquired phosphoglycerate kinase (PGK) genes were identified, with some showing evidence of involvement in salt stress resistance (Figure S13). These findings suggest that HGT has contributed to the adaptive potential of *P. massoniana* by introducing genes that enhance its ability to cope with environmental stressors.

Resin Terpene Biosynthesis and Gene Expression Patterns

Resin terpene biosynthesis plays a critical role in the defense mechanisms of conifer species. In *P. massoniana*, we identified 219 candidate genes encoding enzymes involved in the 22 steps of resin terpene biosynthesis (Figure 3). These include enzymes responsible for the biosynthesis of isopentenyl pyrophosphate (IPP), geranyl diphosphate (GPP), and the final terpenoid products.

Comparative transcriptomic analysis between high- and low-oleoresin-yield *P. massoniana* genotypes revealed higher expression levels of rate-limiting enzymes such as DXS and HMGR in high-yield genotypes (Figure 3B). Furthermore, five GGPPS genes, involved in synthesizing the diterpene precursor, showed enhanced expression in high-oleoresin-yield genotypes. However, terpene synthase (TPS) genes, responsible for synthesizing the final terpenes, did not exhibit clear expression differences between genotypes, suggesting that regulation of terpene biosynthesis may occur at other enzymatic steps.

We further evaluated the expression levels of ten key genes in stem, needle, and root using RNA *in situ* hybridization (Table S10). It confirmed that key genes of the resin terpene biosynthesis pathway were expressed in several cell types, including the epidermis, sclerenchyma, cortex, and xylem resin cells (Figure S14). This indicates that oleoresin biosynthesis is a highly regulated process, with its yield potentially dependent on the activity of rate-limiting enzymes.

Regulatory Networks Controlling Oleoresin Biosynthesis

To further explore the regulation of oleoresin biosynthesis, we performed promoter analyses and co-expression studies focusing on SNP-associated key genes involved in terpene synthesis. These analyses revealed three major gene co-expression modules (Co-expression Modules 1-3), which included genes from the CYP450 and TPS families that are critical for oleoresin biosynthesis (Figure 4).

Six upstream transcription factors (TFs) were identified as potential regulators of these modules: ARF, bHLH, bZIP, ERF, MYB, and WRKY. Among these, MYB binding sites were present in 90% of the promoters of the genes in the co-expression modules, suggesting a central role for MYB in the regulation of oleoresin biosynthesis. Notably, the transcription factor PmMYB4 emerged as a key regulatory component, with strong evidence indicating its interaction with downstream CYP450 genes. Experimental validation using luciferase (LUC) assays and electrophoretic mobility shift assays (EMSA) confirmed the direct interaction between PmMYB4 and the promoter regions of key CYP450 genes (Figure 4). This direct regulation suggests that PmMYB4 plays a pivotal role in modulating the expression of genes involved in oleoresin synthesis. Additionally, PmMYB4 was found to co-localize with another transcription factor, PmbZIP2, to jointly regulate a subset of TPS and CYP450 genes,

further indicating a complex regulatory network controlling oleoresin biosynthesis.

The Population Genetic Structure and the Genetic Basis of Oleoresin Yield

Reanalyzing of 204 samples from various geographical locations [2] using Principal Component Analysis (PCA), STRUCTURE, and Phylogenetic maximum likelihood (ML) tree revealed consistent three distinct genetic clusters, which aligned with the geographical distribution of *P. massoniana*, corresponding to the South, South East, and West China populations (Figure S15). These clusters were well-defined and showed high correspondence with the three major geographical regions of *P. massoniana*, indicating a strong influence of geographic effect on the genetic structure of this species. Phylogenetic tree constructed for a subset of single copy orthologous genes showed a clear partitioning of gene copies into the three identified genetic clusters, suggesting that these clusters have a historical and potentially adaptive significance. From here, the populations have expanded outward, primarily along two trajectories: one towards the south and the other towards the southeast (Figure S15).

The Transcriptome-Wide Association Study (TWAS) of oleoresin yield in *Pinus massoniana* identified 6,064 key genetic markers (SNPs) significantly associated with oleoresin synthesis and production (*P*<=0.01). These SNPs are comprised of several functional protein families and motifs. Notably, kinesin family proteins, which are involved in intracellular transport, were highlighted by the presence of the PFAM domains Kinesin, suggesting a potential role in the cellular mechanisms underlying oleoresin production. The zinc phosphodiesterase ELAC protein, which contains Lactamase B 2 and Lactamase B 4 domains, potentially links RNA processing to oleoresin synthesis. The GTP diphosphokinase CRSH protein, localized in the chloroplast, which contains EF-hand 1, EF-hand 5, HD 4, and RelA SpoT domains, is crucial for energy metabolism and could be a key regulator

in the biosynthesis of oleoresin. Notably, among the strongly associated SNPs, one set of important SNPs related to oleoresin biosynthesis are the terpene synthase family (Table S11). SNPs belonging to the terpene synthase family, marked by terpene synthesis and terpene synthesis C domains, are key enzymes in the biosynthesis of terpenes, which are major components of oleoresin. Additionally, protein NRT1 PTR family, associated with transport and metabolism of nitrogenous compounds, could be indirectly linked to oleoresin yield. SNPs within the small heat shock protein (HSP20) family are known to protect cells against various stresses, potentially including those encountered during oleoresin synthesis. Besides, among the loci significantly associations with oleoresin yield in GWAS, 51 SNPs are annotated to the cytochrome P450 family and *TPS* genes (Table S11). This superfamily of enzymes is known for their role in the metabolism of endogenous and exogenous substances, including the biosynthesis of oleoresin. There are also other SNPs showing strong association with the oleoresin yield, such as proline-rich nuclear receptor coactivator motif, glycosyl hydrolase family 3 N terminal domain, ethylene-responsive transcription factor, protein NRT1 PTR family, N-oligosaccharyl transferase (OST) complex, scalcium-dependent phosphotriesterase superfamily protein. They were all implicated in the regulation or biosynthesis of oleoresin. These findings enhance our understanding of the genetic underpinnings of oleoresin yield and provide a foundation for future genetic improvement strategies in Masson's pine.

The Synthesis of Oleoresin May Be Under the Control of a Coordinated Regulatory System Involving Multiple TFs

SNP-based association analyses provide a suitable approach for identification of key genes and genetic regulatory networks; thus, it may be useful for exploring the regulation of genes in the resin terpene

biosynthesis pathway. Here, we performed a multifaceted approach combining multiple full-length SNP-associated key genes and co-expression network. As a result, we identified several key modules of *CYP450* and *TPS* genes associated with the biosynthesis of oleoresin, that displayed significant co-expression patterns. For instance, key CYP450 genes include CYP720B4 and CYP720B6, while critical TPS genes such as TPS1 and TPS6. These genes were found to be part of three major co-expression modules (Co-expression gene module 1-3), which in turn were linked to six upstream transcription factors (ARF, bHLH, bZIP, ERF, MYB, WRKY) that likely regulate their expression (Figure 4). Notably, MYB binding site was shared by 90% of the promoters of these genes. Thus, the transcription factor PmMYB4 was identified as a key regulatory component in this network, with evidence suggesting its interaction with downstream *CYP450* genes.

Further molecular studies using luciferase (LUC) assays and electrophoretic mobility shift assays (EMSA) provided experimental evidence for the direct interaction between PmMYB4 and the promoter regions of *CYP450* genes, indicating a role for PmMYB4 in the transcriptional regulation of these genes (Figure 4). Additionally, PmMYB4 may act in concert with PmbZIP2 to regulate a subset of downstream *TPS* and *CYP450* genes, with both factors showing co-localization at the molecular level.

The discovery of these regulatory interactions suggests that the synthesis of oleoresin in *P. massoniana* is under the control of a coordinated regulatory system involving multiple TFs. For instance, TFs such as WRKY and bZIP have been implicated in stress responses and may also play a role in the context of oleoresin biosynthesis. This coordinated regulation could be crucial for the plant's ability to adapt to various environmental stresses and optimize oleoresin production.

Discussion

Gymnosperms, a diverse and ancient lineage of plants that originated around 270 million years ago (MYA), are renowned for their ecological importance and evolutionary distinctiveness [80]. Conifers, as a unique lineage of gymnosperms, have captured the attention of biologists with their remarkable diversity, ecological significance, and evolutionary history. In this study, we report the first chromosome-level genome assembly of *Pinus massoniana*, which provides a rich resource for understanding the genomic underpinnings of a species that is both ecologically significant and economically valuable and provides insight into the evolution of the gymnosperm genome. Our findings reveal a complex interplay between genomic architecture, gene family expansions, and adaptive traits, underscoring the evolutionary strategies that enable *P. massoniana* to thrive across Southern China.

Genomic Features and Gene Family Expansion

The assembled genome of *P. massoniana* is approximately 21.91 Gb in size, consistent with other conifer genomes, which are known for their large size and high proportion of repetitive sequences. Similar to other pine species, such as *Pinus tabuliformis* and *Pinus taeda*, the large genome size of *P. massoniana* is primarily driven by the accumulation of long terminal repeat (LTR) transposable elements, particularly those belonging to the Gypsy and Copia families (Table S3) (Nystedt et al., 2013; Liu et al., 2021). The expansion of these elements, especially Gypsy LTR-RTs, within the last 5-30 million years suggests a relatively recent and rapid increase in their activity (Figure S5). This observation is consistent with the hypothesis that transposable elements can drive genome size increase

in plants [3, 4, 81]. The correlation between transposable element bursts, particularly of the Gypsy family, and the accumulation of long introns in the *P. massoniana* genome indicates a significant role for these elements in shaping the genome's architecture. This phenomenon is not unique to *P. massoniana* but is also observed in other gymnosperm genomes, such as *Cunninghamia lanceolata*, *Picea abies*, and *Torreya grandis* [3-6]. Our data support the notion that intron size in gymnosperms is highly variable and generally longer than in angiosperms (Figure S7-S8), which may have implications for gene expression and regulation.

The Adaptive Evolution of *P. massoniana*

Our analysis revealed significant expansions in several gene families associated with stress responses and plant-pathogen interactions, including genes related to the CYP450 and TPS families (Figure 3C-D). These gene families are known to be involved in the biosynthesis of secondary metabolites, such as terpenoids, which play critical roles in plant defense mechanisms. The expansion of these gene families in *P. massoniana* suggests that these genes have been under positive selection, likely contributing to the species' ability to adapt to various biotic and abiotic stresses. The identification of dispersed duplicates and horizontal gene transfer (HGT) events further highlights the role of gene duplication and foreign gene acquisition in the adaptive evolution of *P. massoniana* (Figure S13). Dispersed duplicate genes, which are scattered throughout the genome rather than being arranged in tandem, have been implicated in conferring adaptive advantages by enabling functional diversification. In our study, we observed signatures of adaptive evolution in these dispersed duplicates, particularly in genes involved in stress responses and metabolic pathways (Figure S9). This suggests that gene duplication has played a critical role in the evolutionary success of *P. massoniana*.

HGT events, although relatively rare in plants, have been increasingly recognized as important contributors to plant evolution. In *P. massoniana*, we identified several potential HGT events involving genes related to stress responses and terpenoid biosynthesis. These findings suggest that the acquisition of foreign genes may have facilitated the evolution of novel traits in *P. massoniana*, further enhancing its adaptability. In our analysis, gene families related to terpene biosynthesis, particularly the *CYP450* and *TPS* families, exhibited significant expansion. These families play crucial roles in the synthesis of oleoresin, which serves not only as a defense mechanism against pests and pathogens but also as a vital economic resource for the timber industry.

Evolutionary History

The large genome size of *P. massoniana*, predominantly due to a high content of repetitive sequences—particularly LTR retrotransposons—highlights a dynamic evolutionary history. Our results indicate that approximately 81.2% of the genome comprises repetitive elements, with Gypsy retrotransposons making up 43.59%. Such expansions often correlate with environmental adaptations, as transposable elements can drive genomic diversification and contribute to phenotypic variation [3, 4]. The proliferation of LTR retrotransposons in *P. massoniana*, which coincides with the Miocene epoch (Figure S5), aligns with significant climatic changes, such as global temperatures that likely drove adaptation as previous studies [75, 76].

Furthermore, the identification of two whole-genome duplication (WGD) events provides a framework for understanding the evolution of gene families in *P. massoniana* (Figure 2D). WGDs are known to facilitate gene family expansion, allowing species to adapt to new ecological niches [29].

Our discovery of 57,207 duplicated genes (Figure 2C), mainly resulting from dispersed duplications, supports the notion that genomic redundancy can act as a reservoir for evolutionary innovation, especially in response to biotic and abiotic stressors [82, 83].

Regulatory Networks in Oleoresin Production

The identification of significant SNP markers associated with oleoresin yield presents valuable opportunities for genomic-assisted breeding. Our study identified 6,064 SNPs linked to oleoresin synthesis (Table S11), including those within functional protein families such as kinesin and various transcription factors. Notably, the expansion of the TPS family and the presence of key transcription factors, including PmMYB4, underscore the complexity of the regulatory networks governing oleoresin production.

However, the need for specificity in identifying which *CYP450* and *TPS* genes are involved in oleoresin biosynthesis is crucial. While our findings indicate that PmMYB4 may play a central role in regulating these pathways, further functional validation is necessary to establish definitive connections between these genes and oleoresin yield (Figure 4). The reliance on expression patterns without functional evidence could lead to misinterpretations; Therefore, future research should employ targeted gene editing techniques, such as CRISPR/Cas9, to establish direct links between gene function and oleoresin biosynthesis. By focusing on high oleoresin-yielding genotypes and utilizing identified single nucleotide polymorphism (SNP) markers along with transcription factors like PmMYB4, we can develop cultivars with enhanced oleoresin production and improved stress resistance.

Horizontal Gene Transfer

The presence of horizontally transferred genes in the *P. massoniana* genome adds another layer of complexity to its evolutionary narrative. Our identification of 689 genes likely acquired through horizontal gene transfer (Figure S13; Table S9), including those from fungi and bacteria, suggests that *P. massoniana* has benefited from genetic material that enhances its adaptability to environmental stresses, for example, drought, pest infestations, and pathogen attacks, etc. This phenomenon is consistent with the understanding that HGT can facilitate the acquisition of beneficial traits that promote survival and diversification in changing environments [84].

The functional characterization of these HGT-acquired genes, particularly those involved in stress response and cell wall biosynthesis, warrants further investigation. Understanding how these genes integrate into the existing genetic framework of *P. massoniana* could provide insights into the evolutionary pressures that shaped its current genomic architecture.

Future Directions

Despite the significant insights provided by this study, several areas require further exploration. The specificity of gene functions related to oleoresin biosynthesis needs to be clarified, particularly concerning the roles of various CYP450 and TPS genes. Additionally, the regulatory interactions identified warrant deeper investigation to validate the proposed connections and elucidate the mechanisms underlying oleoresin production. Furthermore, the role of horizontal gene transfer in shaping the adaptive traits of *P. massoniana* should be investigated through functional studies that assess how these genes contribute to stress tolerance and overall fitness. The integration of genomic

resources with ecological and physiological studies will provide a more comprehensive understanding of how *P. massoniana* adapts to its environment.

In conclusion, our study advances the understanding of the genomic and evolutionary dynamics of *Pinus massoniana*, emphasizing its adaptive strategies and the complexity of gene interactions in oleoresin biosynthesis. The insights gained will not only enhance the conservation and management of this ecologically and economically important species but also contribute to the broader understanding of conifer biology and evolution. Future research should focus on addressing the gaps identified in this study to further refine our knowledge of the genetic mechanisms that enable *P. massoniana* to thrive in diverse habitats.

Acknowledgement

This work was supported by funding from Guangxi Science and Technology Base and Talent Special Project (Guike AD19254004); National Key R&D Program of China (2024YFD2201301-1, No.2022YFD2201600; 2022YFD2200602), Bagui Scholar Special Project (2019A26); Bagui Young Scholar Special Project (2019AQ17); the Project of the National Natural Science Foundation of China (nos. 32371906 and 32022057), Forestry and Grassland Science and Technology Innovation Youth Top Talent Project of China (no. 2020132607). The Fundamental Research Funds for the Central Universities [QNTD202305, BFUKF202413].

Author Contribution

YZQ design of the research; CH, CYH and QXH performed the research; XJB and QXH wrote the

manuscript. YZQ obtained funding and is responsible for this article. ZHY, FYH, TJH, CXH, HL, XJK provided valuable suggestions. All authors read and approved the manuscript. CH, QXH, and CYH contributed equally to this work.

Data Availability

The genomic and transcriptomic sequence data generated in this study are available under NCBI BioProject accession PRJNA1240911. The genomic and transcriptomic sequence data reused in this study are available under the NCBI BioProject accessions: PRJNA561037; PRJNA576798; PRJNA595650; PRJNA637602; PRJNA636599; PRJNA647052; PRJNA648715; PRJNA655997; PRJNA656380; PRJNA693351; PRJNA707606; PRJNA743934; PRJNA744619; PRJNA748221; PRJNA749363; PRJNA781761; PRJNA863936; PRJNA667166. All additional supporting data are available in the GigaScience repository, GigaDB [85].

Conflicts of Interest

The authors declare no conflict of interest.

References

1. Mei LN, Li ZC, Yan YJ, Wen Z, Wen XP, Yang ZQ, et al. Identification and functional study of oleoresin terpenoid biosynthesis-related genes in masson pine (*Pinus massoniana* L.) based on transcriptome analysis. Tree Genetics & Genomes. 2020;16 4 doi:Artn 53 10.1007/S11295-020-01448-W.
2. Liu QH, Xie YN, Liu B, Yin HH, Zhou ZC, Feng ZP, et al. A transcriptomic variation map provides insights into the genetic basis of *Pinus massoniana* Lamb. evolution and the association with oleoresin yield. BMC Plant Biol. 2020;20 1 doi:ARTN 375 10.1186/s12870-020-02577-z.
3. Nystedt B, Street NR, Wetterbom A, Zuccolo A, Lin YC, Scofield DG, et al. The Norway spruce genome sequence and conifer genome evolution. Nature. 2013;497 7451:579-84.

doi:10.1038/nature12211.

4. Niu S, Li J, Bo W, Yang W, Zuccolo A, Giacomello S, et al. The Chinese pine genome and methylome unveil key features of conifer evolution. Cell. 2022;185 1:204-17 e14. doi:10.1016/j.cell.2021.12.006.
5. Liu HL, Wang XB, Wang GB, Cui P, Wu SG, Ai C, et al. The nearly complete genome of *Ginkgo biloba* illuminates gymnosperm evolution. Nat Plants. 2021;7 6:748-56. doi:10.1038/s41477-021-00933-x.
6. Lou H, Song L, Li X, Zi H, Chen W, Gao Y, et al. The *Torreya grandis* genome illuminates the origin and evolution of gymnosperm-specific sciadonic acid biosynthesis. Nat Commun. 2023;14 1:1315. doi:10.1038/s41467-023-37038-2.
7. Birol I, Raymond A, Jackman SD, Pleasance S, Coope R, Taylor GA, et al. Assembling the 20 Gb white spruce (*Picea glauca*) genome from whole-genome shotgun sequencing data. Bioinformatics. 2013;29 12:1492-7.
8. Neale DB, Wegrzyn JL, Stevens KA, Zimin AV, Puiu D, Crepeau MW, et al. Decoding the massive genome of loblolly pine using haploid DNA and novel assembly strategies. Genome Biology,15,3(2014-03-20). 2014;15 3:R59.
9. Gagalova KK, Warren RL, Coombe L, Wong J, Nip KM, Saint Yuen MM, et al. Spruce giga-genomes: structurally similar yet distinctive with differentially expanding gene families and rapidly evolving genes. Plant J. 2022;111 5:1469-85. doi:10.1111/tpj.15889.
10. Kovach A, Wegrzyn JL, Parra G, Holt C, Bruening GE, Loopstra CA, et al. The Pinus taeda genome is characterized by diverse and highly diverged repetitive sequences. BMC Genomics. 2010;11:420. doi:10.1186/1471-2164-11-420.
11. Shi Z, Deng X, Zeng L, Shi S, Lei L and Xiao W. Acclimation Strategy of Masson Pine (Pinus massoniana) by Limiting Flavonoid and Terpenoid Production under Low Light and Drought. Int J Mol Sci. 2022;23 15 doi:10.3390/ijms23158441.
12. Xu J, Jian Z, Zhang Y, Deng X, Lei L, Zeng L, et al. Nutrient Variations and Their Use Efficiency of Pinus massoniana Seedling Tissues in Response to Low Phosphorus Conditions. Forests. 2024;15 2 doi:10.3390/f15020351.
13. Rodrigues-Correa KCD, de Lima JC and Fett-Neto AG. Pine oleoresin: tapping green chemicals, biofuels, food protection, and carbon sequestration from multipurpose trees. Food Energy Secur. 2012;1 2:81-93. doi:10.1002/fes3.13.
14. Rodgers-Melnick E, Mane SP, Dharmawardhana P, Slavov GT, Crasta OR, Strauss SH, et al. Contrasting patterns of evolution following whole genome versus tandem duplication events in Populus. Genome Res. 2012;22 1:95-105. doi:10.1101/gr.125146.111.
15. Vallinayagam R, Vedharaj S, Yang WM, Lee PS, Chua KJE and Chou SK. Pine oil-biodiesel blends: A double biofuel strategy to completely eliminate the use of diesel in a diesel engine. Appl Energ. 2014;130:466-73. doi:10.1016/j.apenergy.2013.11.025.
16. Jellyfish (2011). Jellyfish (Version 2.1.4). https://github.com/gmarcais/Jellyfish.
17. Vurture, Gregory W, Sedlazeck, Fritz J, Nattestad, Maria, et al. GenomeScope: fast reference-free genome profiling from short reads. Bioinformatics. 2017.
18. Liu H, Wu S, Li A and Ruan J. SMARTdenovo: a de novo assembler using long noisy reads. 2021.

19. Hu J, Fan J, Sun Z and Liu S. NextPolish: a fast and efficient genome polishing tool for long-read assembly. Bioinformatics. 2020;36 7:2253-5. doi:10.1093/bioinformatics/btz891.
20. Li H and Durbin R. Fast and accurate short read alignment with Burrows-Wheeler transform. Bioinformatics. 2009;25 14:1754-60. doi:10.1093/bioinformatics/btp324.
21. Kent WJ. BLAT—The BLAST-Like Alignment Tool. Genome Res. 2002;12 4:656-64.
22. Simao FA, Waterhouse RM, Ioannidis P, Kriventseva EV and Zdobnov EM. BUSCO: assessing genome assembly and annotation completeness with single-copy orthologs. Bioinformatics. 2015;31 19:3210-2. doi:10.1093/bioinformatics/btv351.
23. Jens, Keilwagen, Frank, Hartung, Jan and Grau. GeMoMa: Homology-Based Gene Prediction Utilizing Intron Position Conservation and RNA-seq Data. Methods Mol Biol. 2019; 1962:161-77.
24. Korf I. Gene finding in novel genomes. BMC bioinformatics. 2004;5:59. doi:10.1186/1471-2105-5-59.
25. Stanke M, Keller O, Gunduz I, Hayes A, Waack S and Morgenstern B. AUGUSTUS: ab initio prediction of alternative transcripts. Nucleic Acids Res. 2006;34 Web Server issue:W435-9. doi:10.1093/nar/gkl200.
26. Zhou F, Dong DN, Li WW, Jiang XY, Wickert J and Schuh H. GAMP: An open-source software of multi-GNSS precise point positioning using undifferenced and uncombined observations. Gps Solut. 2018;22 2 doi:ARTN 3310.1007/s10291-018-0699-9.
27. Haas BJ, Salzberg SL, Zhu W, Pertea M, Allen JE, Orvis J, et al. Automated eukaryotic gene structure annotation using EVidenceModeler and the Program to Assemble Spliced Alignments. Genome Biol. 2008;9 1:R7. doi:10.1186/gb-2008-9-1-r7.
28. Holt C and Yandell M. MAKER2: an annotation pipeline and genome-database management tool for second-generation genome projects. BMC bioinformatics. 2011;12:491. doi:10.1186/1471-2105-12-491.
29. De Bie T, Cristianini N, Demuth JP and Hahn MW. CAFE: a computational tool for the study of gene family evolution. Bioinformatics. 2006;22 10:1269-71. doi:10.1093/bioinformatics/btl097.
30. Tang H, Bowers JE, Wang X, Ming R, Alam M and Paterson AH. Synteny and Collinearity in Plant Genomes. Science. 2008;320 5875:486-8.
31. Jurka J, Kapitonov VV, Pavlicek A, Klonowski P, Kohany O and Walichiewicz J. Repbase update, a database of eukaryotic repetitive elements. Cytogenetic and Genome Research. 2005;110 1-4:462-7. doi:10.1159/000084979.
32. Price AL, Jones NC and Pevzner PA. De novo identification of repeat families in large genomes. Bioinformatics. 2005;21:I351-I8. doi:10.1093/bioinformatics/bti1018.
33. Xu Z and Wang H. LTR_FINDER: an efficient tool for the prediction of full-length LTR retrotransposons. Nucleic Acids Res. 2007;35:W265-W8. doi:10.1093/nar/gkm286.
34. Qiao X, Li Q, Yin H, Qi K, Li L, Wang R, et al. Gene duplication and evolution in recurring polyploidization-diploidization cycles in plants. Genome Biol. 2019;20 1:38. doi:10.1186/s13059-019-1650-2.
35. MCScanX (2012). MCScanX (Version 1.0.0). https://github.com/wyp1125/MCScanX.
36. Zhang Z, Xiao J, Wu J, Zhang H, Liu G, Wang X, et al. ParaAT: a parallel tool for constructing

multiple protein-coding DNA alignments. Biochem Biophys Res Commun. 2012;419 4:779-81. doi:10.1016/j.bbrc.2012.02.101.
37. Yu ZJ. KaKs_Calculator 2.0: A Toolkit Incorporating Gamma-Series Methods and Sliding Window Strategies. Genomics, Proteomics & Bioinformatics. 2010;8 1:70-80.
38. Chen SF, Zhou YQ, Chen YR and Gu J. fastp: an ultra-fast all-in-one FASTQ preprocessor. Bioinformatics. 2018;34 17:884-90. doi:10.1093/bioinformatics/bty560.
39. Kim D, Paggi JM, Park C, Bennett C and Salzberg SL. Graph-based genome alignment and genotyping with HISAT2 and HISAT-genotype. Nat Biotechnol. 2019;37 8:907-15.
40. Kovaka S, Zimin AV, Pertea GM, Razaghi R, Salzberg SL and Pertea M. Transcriptome assembly from long-read RNA-seq alignments with StringTie2. Genome Biol. 2019;20 1:278. doi:Artn 278 10.1186/S13059-019-1910-1.
41. Cantalapiedra CP, Hernandez-Plaza A, Letunic I, Bork P and Huerta-Cepas J. eggNOG-mapper v2: Functional Annotation, Orthology Assignments, and Domain Prediction at the Metagenomic Scale. Mol Biol Evol. 2021;38 12:5825-9. doi:10.1093/molbev/msab293.
42. Yu GC, Wang LG, Han YY and He QY. clusterProfiler: an R Package for Comparing Biological Themes Among Gene Clusters. Omics. 2012;16 5:284-7. doi:10.1089/omi.2011.0118.
43. Bailey TL, Johnson J, Grant CE and Noble WS. The MEME Suite. Nucleic Acids Res. 2015;43 W1:W39-W49. doi:10.1093/nar/gkv416.
44. Edgar RC. MUSCLE: multiple sequence alignment with high accuracy and high throughput. Nucleic Acids Res. 2004;32 5:1792-7. doi:10.1093/nar/gkh340.
45. Minh BQ, Schmidt HA, Chernomor O, Schrempf D, Woodhams MD, von Haeseler A, et al. IQ-TREE 2: New Models and Efficient Methods for Phylogenetic Inference in the Genomic Era. Mol Biol Evol. 2020;37 5:1530-4. doi:10.1093/molbev/msaa015.
46. Li RQ, Yu C, Li YR, Lam TW, Yiu SM, Kristiansen K, et al. SOAP2: an improved ultrafast tool for short read alignment. Bioinformatics. 2009;25 15:1966-7. doi:10.1093/bioinformatics/btp336.
47. Nguyen LT, Schmidt HA, von Haeseler A and Minh BQ. IQ-TREE: A Fast and Effective Stochastic Algorithm for Estimating Maximum-Likelihood Phylogenies. Mol Biol Evol. 2015;32 1:268-74. doi:10.1093/molbev/msu300.
48. Letunic I and Bork P. Interactive Tree Of Life (iTOL) v4: recent updates and new developments. Nucleic Acids Res. 2019;47 W1:W256-W9. doi:10.1093/nar/gkz239.
49. Purcell S, Neale B, Todd-Brown K, Thomas L, Ferreira MAR, Bender D, et al. PLINK: A tool set for whole-genome association and population-based linkage analyses. Am J Hum Genet. 2007;81 3:559-75. doi:10.1086/519795.
50. Price AL, Patterson NJ, Plenge RM, Weinblatt ME, Shadick NA and Reich D. Principal components analysis corrects for stratification in genome-wide association studies. Nat Genet. 2006;38 8:904-9. doi:10.1038/ng1847.
51. Alexander DH, Novembre J and Lange K. Fast model-based estimation of ancestry in unrelated individuals. Genome Res. 2009;19 9:1655-64. doi:10.1101/gr.094052.109.
52. Danecek P, Auton A, Abecasis G, Albers CA, Banks E, DePristo MA, et al. The variant call format and VCFtools. Bioinformatics. 2011;27 15:2156-8. doi:10.1093/bioinformatics/btr330.
53. Bradbury PJ, Zhang Z, Kroon DE, Casstevens TM, Ramdoss Y and Buckler ES. TASSEL:

software for association mapping of complex traits in diverse samples. Bioinformatics. 2007;23 19:2633-5. doi:10.1093/bioinformatics/btm308.

54. Tian F, Yang DC, Meng YQ, Jin JP and Gao G. PlantRegMap: charting functional regulatory maps in plants. Nucleic Acids Res. 2020;48 D1:D1104-D13. doi:10.1093/nar/gkz1020.

55. Capella-Gutierrez S, Silla-Martinez JM and Gabaldon T. trimAl: a tool for automated alignment trimming in large-scale phylogenetic analyses. Bioinformatics. 2009;25 15:1972-3. doi:10.1093/bioinformatics/btp348.

56. Minh BQ, Nguyen MAT and von Haeseler A. Ultrafast Approximation for Phylogenetic Bootstrap. Mol Biol Evol. 2013;30 5:1188-95. doi:10.1093/molbev/mst024.

57. Shen XX, Opulente DA, Kominek J, Zhou X, Steenwyk JL, Buh KV, et al. Tempo and Mode of Genome Evolution in the Budding Yeast Subphylum. Cell. 2018;175 6:1533-45. doi:10.1016/j.cell.2018.10.023.

58. Buchfink B, Reuter K and Drost HG. Sensitive protein alignments at tree-of-life scale using DIAMOND. Nat Methods. 2021;18 4:366-8. doi:10.1038/s41592-021-01101-x.

59. Katoh K and Standley DM. MAFFT Multiple Sequence Alignment Software Version 7: Improvements in Performance and Usability. Mol Biol Evol. 2013;30 4:772-80. doi:10.1093/molbev/mst010.

60. Paradis E, Claude J and Strimmer K. APE: Analyses of Phylogenetics and Evolution in R language. Bioinformatics. 2004;20 2:289-90. doi:10.1093/bioinformatics/btg412.

61. Schliep KP. phangorn: phylogenetic analysis in R. Bioinformatics. 2011;27 4:592-3. doi:10.1093/bioinformatics/btq706.

62. Wu TY, Goh H, Azodi CB, Krishnamoorthi S, Liu MJ and Urano D. Evolutionarily conserved hierarchical gene regulatory networks for plant salt stress response. Nature plants. 2021;7 6:787-99. doi:10.1038/s41477-021-00929-7.

63. Guo LY, Wang S, Nie YQ, Shen YR, Ye XX and Wu WW. Convergent evolution of AP2/ERF III and IX subfamilies through recurrent polyploidization and tandem duplication during eudicot adaptation to paleoenvironmental changes. Plant Commun. 2022;3 6:15. doi:Artn 100420 10.1016/J.Xplc.2022.100420.

64. plant_rgenes (2019). plant_rgenes (Version ASTREL_v1). https://github.com/krasileva-group/plant_rgenes/.

65. Bailey PC, Schudoma C, Jackson W, Baggs E, Dagdas G, Haerty W, et al. Dominant integration locus drives continuous diversification of plant immune receptors with exogenous domain fusions. Genome Biol. 2018;19 1:23. doi:10.1186/s13059-018-1392-6.

66. Wheeler TJ and Eddy SR. nhmmer: DNA homology search with profile HMMs. Bioinformatics. 2013;29 19:2487-9. doi:10.1093/bioinformatics/btt403.

67. Celedon JM and Bohlmann J. Oleoresin defenses in conifers: chemical diversity, terpene synthases and limitations of oleoresin defense under climate change. New Phytol. 2019;224 4:1444-63. doi:10.1111/nph.15984.

68. Tholl D and Lee S. Terpene Specialized Metabolism in *Arabidopsis thaliana*. The arabidopsis book. 2011;9:e0143. doi:10.1199/tab.0143.

69. Fishilevich E, Bowling AJ, Frey MLF, Wang PH, Lo W, Rangasamy M, et al. RNAi targeting of rootworm *Troponin* I transcripts confers root protection in maize. Insect Biochem Mol Biol.

2019;104:20-9. doi:10.1016/j.ibmb.2018.09.006.
70. Chen SS, Tan SX, Jin ZL, Wu JD, Zhao YY, Xu WJ, et al. The transcriptional landscape of pattern/effector-triggered immunity and how PagWRKY18 involved in it. Plant Cell Environ. 2024;47 6:2074-92. doi:10.1111/pce.14860.
71. Hellens RP, Allan AC, Friel EN, Bolitho K, Grafton K, Templeton MD, et al. Transient expression vectors for functional genomics, quantification of promoter activity and RNA silencing in plants. Plant Methods. 2005;1 doi:Artn 13 10.1186/1746-4811-1-13.
72. Chen HM, Zou Y, Shang YL, Lin HQ, Wang YJ, Cai R, et al. Firefly luciferase complementation imaging assay for protein-protein interactions in plants. Plant Physiol. 2008;146 2:368-76. doi:10.1104/pp.107.111740.
73. Su WL, Bao Y, Lu YY, He F, Wang S, Wang DL, et al. Poplar Autophagy Receptor NBR1 Enhances Salt Stress Tolerance by Regulating Selective Autophagy and Antioxidant System. Front Plant Sci. 2021;11 doi:Artn 568411 10.3389/Fpls.2020.568411.
74. Steinthorsdottir M, Coxall HK, de Boer AM, Huber M, Barbolini N, Bradshaw CD, et al. The Miocene: The Future of the Past. Paleoceanography and Paleoclimatology. 2021;36 4 doi:10.1029/2020pa004037.
75. Zachos J, Pagani M, Sloan L, Thomas E and Billups K. Trends, rhythms, and aberrations in global climate 65 Ma to present. Science. 2001;292 5517:686-93. doi:10.1126/science.1059412.
76. Reuter M, Kern AK, Harzhauser M, Kroh A and Piller WE. Global warming and South Indian monsoon rainfall-lessons from the Mid-Miocene. Gondwana Res. 2013;23 3:1172-7. doi:10.1016/j.gr.2012.07.015.
77. Yang Y, Zhou Y, Chi Y, Fan B and Chen Z. Characterization of Soybean WRKY Gene Family and Identification of Soybean WRKY Genes that Promote Resistance to Soybean Cyst Nematode. Sci Rep-Uk. 2017;7 1:17804.
78. Nie YQ, Guo LY, Cui FQ, Shen YR, Ye XX, Deng DY, et al. Innovations and stepwise evolution of CBFs/DREB1s and their regulatory networks in angiosperms. J Integr Plant Biol. 2022;64 11:2111-25. doi:10.1111/jipb.13357.
79. Guo L, Wang S, Nie Y, Shen Y, Ye X and Wu W. Convergent evolution of AP2/ERF III and IX subfamilies through recurrent polyploidization and tandem duplication during eudicot adaptation to paleoenvironmental changes. Plant Commun. 2022;3 6:15.
80. Davis CC and Schaefer H. Plant evolution: pulses of extinction and speciation in gymnosperm diversity. Curr Biol. 2011;21 24:R995-8. doi:10.1016/j.cub.2011.11.020.
81. Björn N, Street NR, Anna W, Andrea Z, Yao-Cheng L, Scofield DG, et al. The Norway spruce genome sequence and conifer genome evolution. Nature. 2013;497 7451:579-84.
82. Almeida-Silva F and Van de Peer Y. Whole-genome Duplications and the Long-term Evolution of Gene Regulatory Networks in Angiosperms. Molecular biology and evolution. 2023;40 7 doi:10.1093/molbev/msad141.
83. Panchy N, Lehti-Shiu M and Shiu SH. Evolution of Gene Duplication in Plants. Plant Physiol. 2016;171 4:2294-316. doi:10.1104/pp.16.00523.
84. Wu SW, Fang CW, Li ZW, Wang YB, Pan SS, Wu YR, et al. ATP-Binding Cassette G Transporters and Their Multiple Roles Especially for Male Fertility in Arabidopsis, Rice and

Maize. Int J Mol Sci. 2022;23 16 doi:Artn 9304 10.3390/Ijms23169304.
85. Chen H; Qin X; Chen Y; Zhang H; Feng Y; Tan J; Chen X; Hu L; Xie J; Xie J; Yang Z (2025): Supporting data for "Chromosome-Level Genome Assembly of *Pinus massoniana* provides insights into Conifer Adaptive Evolution" GigaScience Database. https://doi.org/10.5524/102688

Figures

Figure 1. Genome assembly and features of *P. massoniana.* Morphology of *P. massoniana* (A1), *P. massoniana* cones at different developmental stages (A2-A9), different growth stages from seed to mature tree (A10-A14), resin tapping (A15), timber (A16). (B) Genome quality assessment results by using BUSCO for *P. massoniana*. (C) Hi-C contact matrices of the twelve pseudomolecules of the final assembly. (D) Distribution of *P. massoniana* genomic features. The tracks from outer to inner circles represent different genomic features as indicated.

Figure 2. Gene family evolution in *P. massoniana.* (A) Gene family expansion and contraction across the thirteen plant species. The numbers of gene families in last common ancestors are highlighted in green; the expansion and contraction of gene families in the sub-branches are highlighted in red and blue, respectively. Number on the coordinate axis represents the divergence time of each branch. In

the right panel, boxplots indicate the intron lengths of 13 species. (**B)** Barplots indicates the number of different gene categories. (**C)** The number of genes derived from different duplication event, including WGD, transposed, tandem, proximal and dispersed duplication. (**D)** The distribution of *Ks* values of the WGD gene pairs of *P. massoniana, P. tabuliformis, G. biloba,* and *S. giganteum*. **(E)** The distribution of *Ks* values of the dispersed gene pairs of the *P. massoniana, P. tabuliformis, G. biloba,* and *S. giganteum*.

Figure 3. The resin terpene biosynthesis pathways and phylogenetics of *TPS* family and conversed motifs in *P. massoniana*. (A) The resin terpene biosynthesis pathways in *P. massoniana*. (**B)** Different genes of key enzymes in terpenoid pathways are shown in heatmaps. LT represents the sample of trunk xylem with low yield oleoresin, LN represents the sample of needle with low yield oleoresin, HT represents the sample of trunk xylem with high yield oleoresin, HN represents the sample of needle with high yield oleoresin. (**C)** Phylogenetic ML tree of *TPS* genes of *P. massoniana*. (**D)** Number of homologous genes in each subfamily of *TPS* genes from four gymnosperms (P. *massoniana*, *P. tabuliformis*, *G. biloba*, *S. giganteum*) and three angiosperms (*A. thaliana*, *O. sativa*, *A. trichopoda*). The composition of domains (α, β, and γ) and conversed motifs (‘DXDD’, ‘DDXXD’, and ‘NSE/DTE’) are noted for each subfamily. (**E)** The conversed motifs (‘DXDD’, ‘DDXXD’, and ‘NSE/DTE’) in angiosperms, gymnosperms and *P. massoniana*.

Figure 4. **Enrichment analysis of the promoters of co-expression modules of *CYP450*, *TPS* genes and interactions between PmMYB4 and PmCYP450.15, PmbZIP2. (A)** Co-expression modules of *CYP450* and *TPS* genes generated by WGCNA analysis, the transcription factors labeled specifically were shared by more than 80% of all genes. *TPS_1*: *gmmutg18241G000030.2*; *TPS_2*: *gmmutg17794G000150.1*; *TPS_3*: *gmmutg42131G000020.1*; *CYP450_1*: *gmmutg68017G000020.1*; *CYP450_2*: *gmmutg26354G000020.1*; *CYP450_3*: *gmmutg20443G000020.1*; *CYP450_4*: *gmmutg178448G000020.1*; *CYP450_5*: *STRG.13291.1.p1*; *CYP450_6*: *gmmutg133003G000010.1*; *CYP450_7*: *gmmutg388649G000010.2*; *CYP450_8*: *gmmutg17794G000030.1*; *CYP450_9*: *gmmutg1680G000040.1*; *CYP450_10*: *MSTRG.47921.2.p1*; *CYP450_11*: *gmmutg101230G000060.1*; *CYP450_12*: *gmmutg5617G000080.1*; *CYP450_13*: *STRG.13290.1.p1*; *CYP450_14*: *gmmutg6309G000040.2*; *CYP450_15*: *MSTRG.30814.1.p1*; *CYP450_16*: *STRG.25141.1.p1*; *CYP450_17*: *gmmutg11433G000020.1*; *CYP450_18*: *gmmutg1483G000030.1*; *CYP450_19*: *gmmutg269G000120.1*; *CYP450_20*: *gmmutg1771G000090.1*; *PmMYB4*: gmmutg10484G000010.1; *PmbZIP2*: gmmutg3867G000050.1. (B) Representative luciferase luminescence image of *Nicotiana benthamiana* leaves co-infiltrated with the agrobacterial strains containing PmCYP450.15pro-Luc and PmMYB4-62-SK. Tobacco leaves injected with empty vector controls, pGreenII 0800-LUC and pGreenII 62-SK, were used as a negative control. (**C)** EMSA

assays was applied to identify the interactions between *GSTMYB4* protein and the promoter gene. Here, 10 and 100 unlabelled probes and probe mutants were used in the competition experiment. **(D).** Subcellular localization of cYFP-bZIP4 and nYFP-MYB4 in transiently expressed tobacco leaves. Scale bar =10μm.

Figure 1

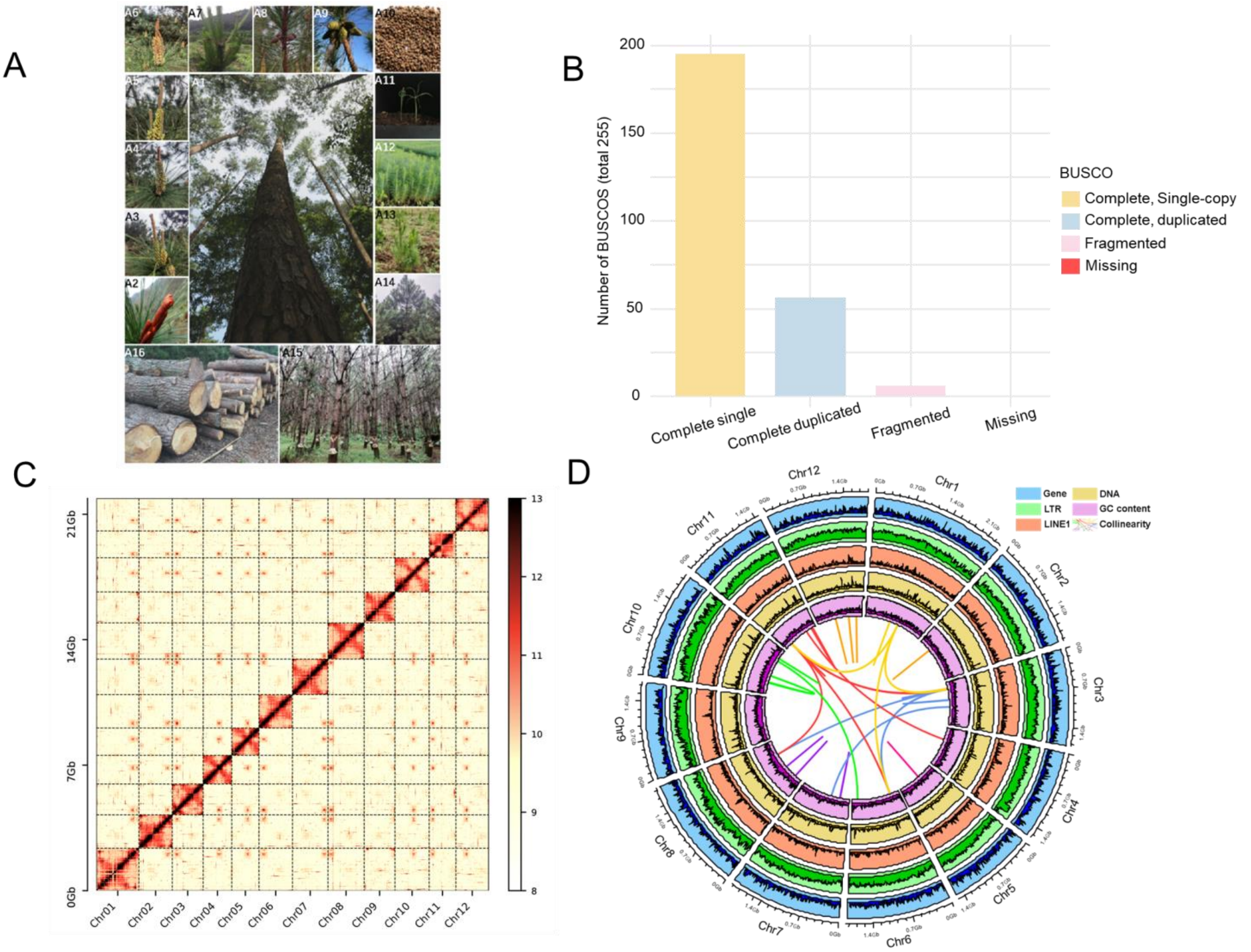
A
A1
A2
A3
A4
A6
A7
A8
A9
A10
A11
A12
A13
A14
A15
A16
B
Number of BUSCOS (total 255)
0
50
100
150
200
Complete single
Complete duplicated
Fragmented
Missing
BUSCO
Complete, Single-copy
Complete, duplicated
Fragmented
Missing
C
0Gb
7Gb
14Gb
21Gb
Chr01
Chr02
Chr03
Chr04
Chr05
Chr06
Chr07
Chr08
Chr09
Chr10
Chr11
Chr12
8
9
10
11
12
13
D
Gene
DNA
LTR
GC content
LINE1
Collinearity
Chr1
Chr2
Chr3
Chr4
Chr5
Chr6
Chr7
Chr8
Chr9
Chr10
Chr11
Chr12

Figure 2

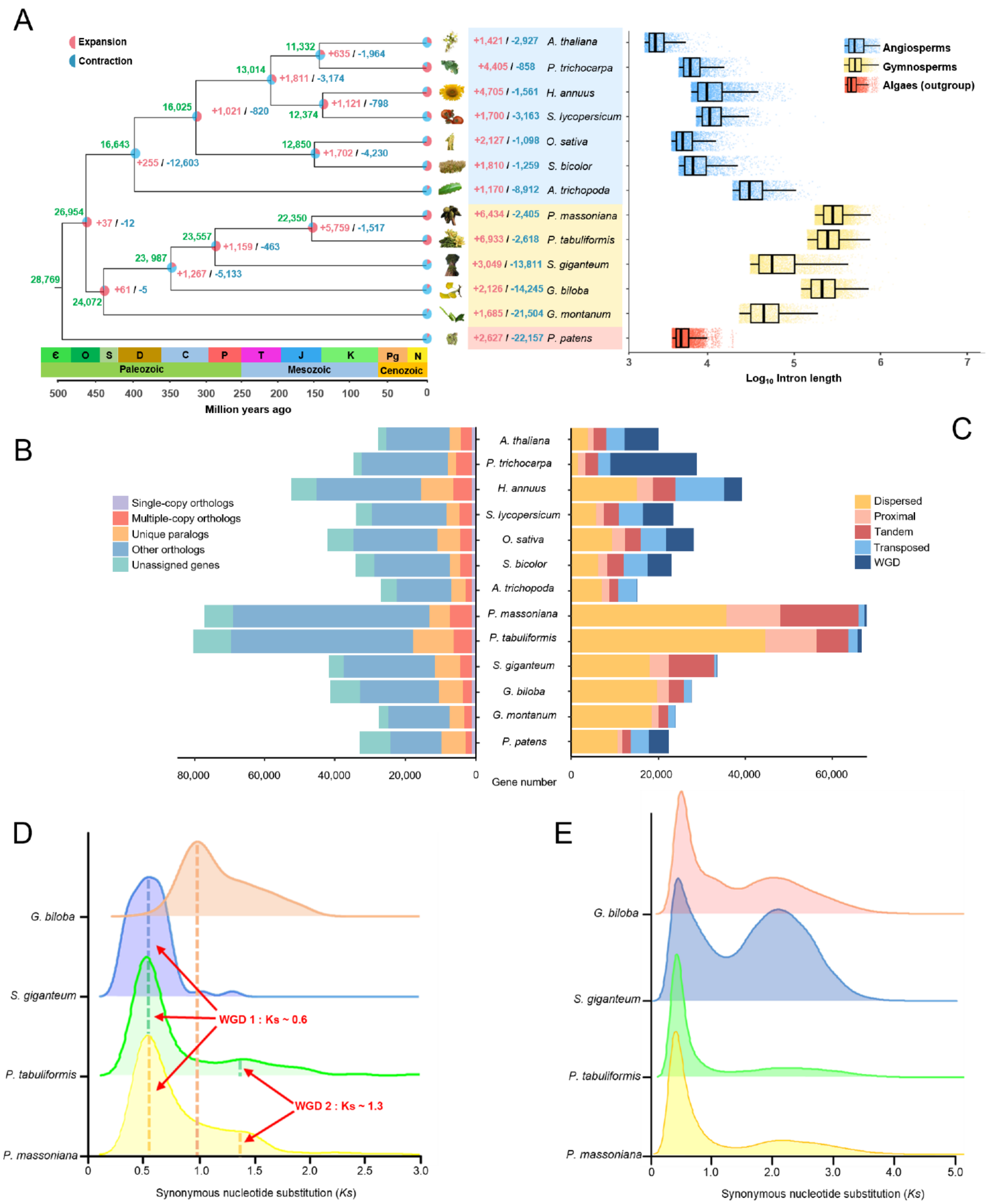

A
Expansion
Contraction
11,332
+635 / -1,964
13,014
+1,811 / -3,174
+1,121 / -798
16,025
+1,021 / -820
12,374
16,643
12,850
+1,702 / -4,230
+255 / -12,603
26,954
+37 / -12
22,350
+5,759 / -1,517
23,557
+1,159 / -463
23, 987
+1,267 / -5,133
28,769
+61 / -5
24,072
+1,421 / -2,927 A. thaliana
+4,405 / -858 P. trichocarpa
+4,705 / -1,561 H. annuus
+1,700 / -3,163 S. lycopersicum
+2,127 / -1,098 O. sativa
+1,810 / -1,259 S. bicolor
+1,170 / -8,912 A. trichopoda
+6,434 / -2,405 P. massoniana
+6,933 / -2,618 P. tabuliformis
+3,049 / -13,811 S. giganteum
+2,126 / -14,245 G. biloba
+1,685 / -21,504 G. montanum
+2,627 / -22,157 P. patens
Є O S D C P T J K Pg N
Paleozoic
Mesozoic
Cenozoic
500 450 400 350 300 250 200 150 100 50 0
Million years ago
Angiosperms
Gymnosperms
Algaes (outgroup)
3 4 5 6 7
Log10 Intron length
B
Single-copy orthologs
Multiple-copy orthologs
Unique paralogs
Other orthologs
Unassigned genes
80,000 60,000 40,000 20,000 0
Gene number
C
Dispersed
Proximal
Tandem
Transposed
WGD
0 20,000 40,000 60,000
D
G. biloba
S. giganteum
P. tabuliformis
P. massoniana
WGD 1 : Ks ~ 0.6
WGD 2 : Ks ~ 1.3
0 0.5 1.0 1.5 2.0 2.5 3.0
Synonymous nucleotide substitution (Ks)
E
G. biloba
S. giganteum
P. tabuliformis
P. massoniana
0 1.0 2.0 3.0 4.0 5.0
Synonymous nucleotide substitution (Ks)

Figure 3

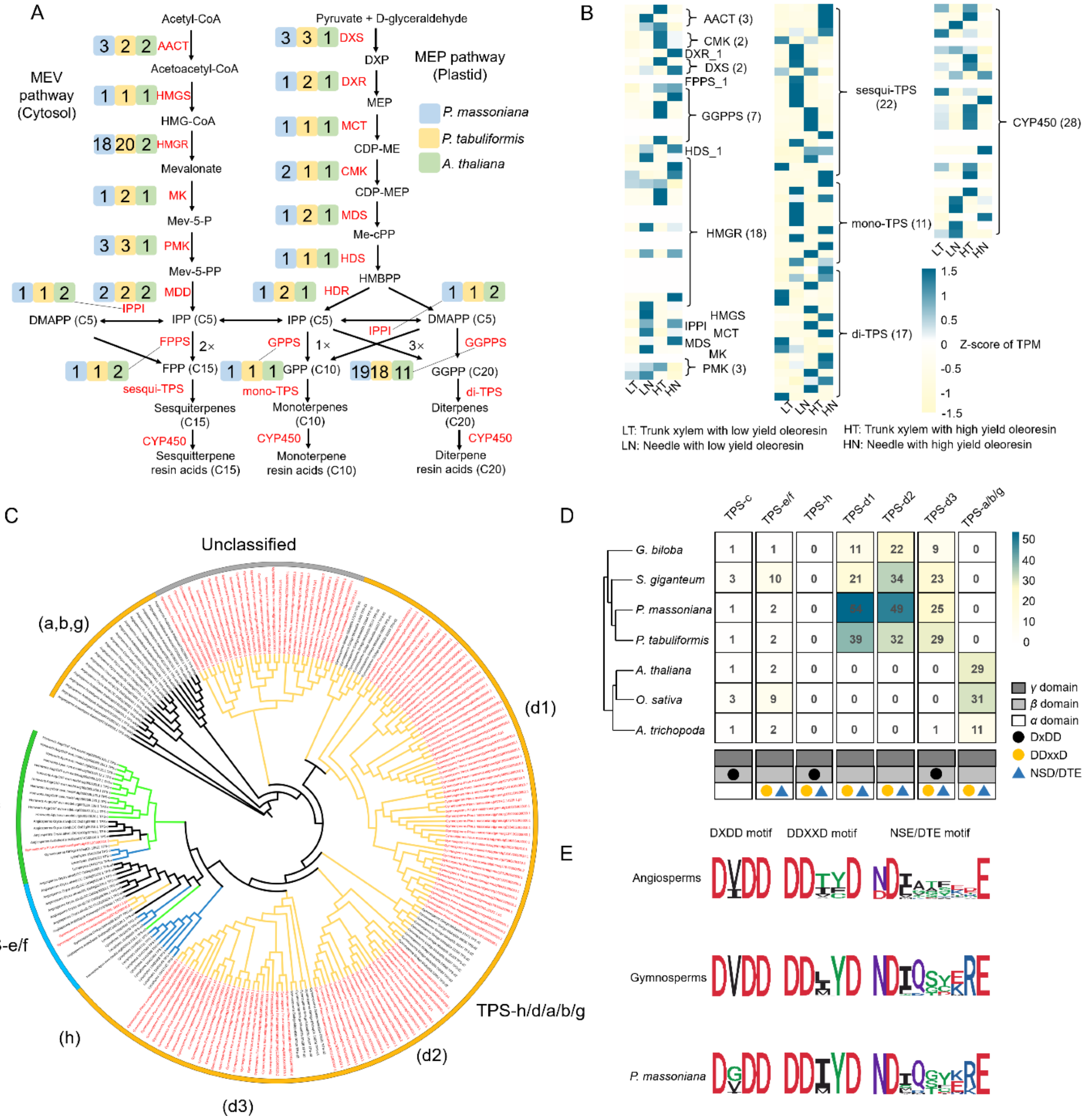
A
Acetyl-CoA
AACT
Acetoacetyl-CoA
HMGS
HMG-CoA
HMGR
Mevalonate
MK
Mev-5-P
PMK
Mev-5-PP
MDD
IPPI
MEV pathway (Cytosol)
DMAPP (C5)
IPP (C5)
FPPS
FPP (C15)
sesqui-TPS
Sesquiterpenes (C15)
CYP450
Sesquitterpene resin acids (C15)
Pyruvate + D-glyceraldehyde
DXS
DXP
DXR
MEP
MCT
CDP-ME
CMK
CDP-MEP
MDS
Me-cPP
HDS
HMBPP
HDR
MEP pathway (Plastid)
P. massoniana
P. tabuliformis
A. thaliana
GPPS
GPP (C10)
mono-TPS
Monoterpenes (C10)
Monoterpene resin acids (C10)
GGPPS
GGPP (C20)
di-TPS
Diterpenes (C20)
Diterpene resin acids (C20)
B
AACT (3)
CMK (2)
DXR_1
DXS (2)
FPPS_1
GGPPS (7)
HDS_1
HMGR (18)
HMGS
IPPI
MCT
MDS
MK
PMK (3)
sesqui-TPS (22)
mono-TPS (11)
di-TPS (17)
CYP450 (28)
LT LN HT HN
Z-score of TPM
LT: Trunk xylem with low yield oleoresin
LN: Needle with low yield oleoresin
HT: Trunk xylem with high yield oleoresin
HN: Needle with high yield oleoresin
C
Unclassified
(a,b,g)
(d1)
TPS-c
TPS-e/f
(h)
TPS-h/d/a/b/g
(d2)
(d3)
D
TPS-c TPS-e/f TPS-h TPS-d1 TPS-d2 TPS-d3 TPS-a/b/g
G. biloba 1 1 0 11 22 9 0
S. giganteum 3 10 0 21 34 23 0
P. massoniana 1 2 0 49 25 0
P. tabuliformis 1 2 0 39 32 29 0
A. thaliana 1 2 0 0 0 0 29
O. sativa 3 9 0 0 0 0 31
A. trichopoda 1 2 0 0 0 1 11
γ domain
β domain
α domain
DxDD
DDxxD
NSD/DTE
E
DXDD motif
DDXXD motif
NSE/DTE motif
Angiosperms
Gymnosperms
P. massoniana

Figure 4

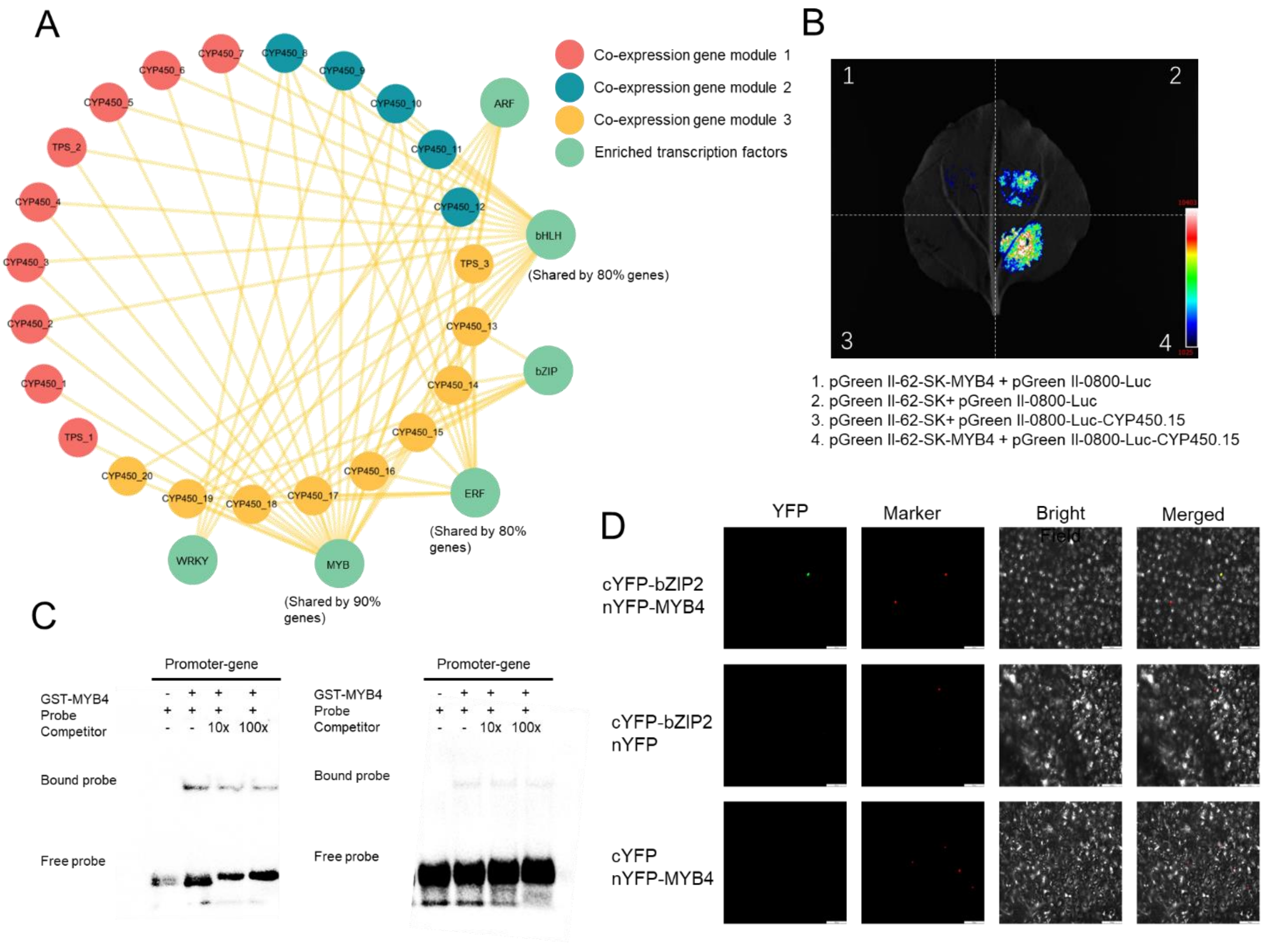

A
Co-expression gene module 1
Co-expression gene module 2
Co-expression gene module 3
Enriched transcription factors
CYP450_1
CYP450_2
CYP450_3
CYP450_4
CYP450_5
CYP450_6
CYP450_7
CYP450_8
CYP450_9
CYP450_10
CYP450_11
CYP450_12
CYP450_13
CYP450_14
CYP450_15
CYP450_16
CYP450_17
CYP450_18
CYP450_19
CYP450_20
TPS_1
TPS_2
TPS_3
ARF
bHLH
(Shared by 80% genes)
bZIP
ERF
(Shared by 80% genes)
MYB
(Shared by 90% genes)
WRKY
B
1
2
3
4
1. pGreen II-62-SK-MYB4 + pGreen II-0800-Luc
2. pGreen II-62-SK+ pGreen II-0800-Luc
3. pGreen II-62-SK+ pGreen II-0800-Luc-CYP450.15
4. pGreen II-62-SK-MYB4 + pGreen II-0800-Luc-CYP450.15
C
Promoter-gene
GST-MYB4 - + + +
Probe + + + +
Competitor - - 10x 100x
Bound probe
Free probe
Promoter-gene
GST-MYB4 - + + +
Probe + + + +
Competitor - - 10x 100x
Bound probe
Free probe
D
YFP
Marker
Bright Field
Merged
cYFP-bZIP2
nYFP-MYB4
cYFP-bZIP2
nYFP
cYFP
nYFP-MYB4

Click here to access/download
Supplementary Material
Supplemental Materials_04182025.docx

Supplemental table

Click here to access/download
Supplementary Material
supplementary tables.xlsx
