# Supplementary material for: Chromosome-level genome assembly of Pinus massoniana provides insights into conifer adaptive evolution
Source: Gigascience. 2025 May 30;14:giaf056. doi: 10.1093/gigascience/giaf056 (PMC12122422; doi:10.1093/gigascience/giaf056)
Supplement: giaf056_Authors_Response_To_Reviewer_Comments_original_submission [file giaf056_authors_response_to_reviewer_comments_original_submission.pdf]

Author's Response To Reviewer Comments

Cover letter for “Chromosome-Level Genome Assembly of Pinus massoniana Provides Insights into Conifer Adaptive Evolution”

Dear Editor and Reviewers,

Thank you for your valuable feedback on our manuscript, "Chromosome-Level Genome Assembly of Pinus massoniana Provides Insights into Conifer Adaptive Evolution". We appreciate the time and effort you have invested in reviewing our work and providing constructive comments. Below, we address each of your concerns and outline the revisions made to improve the manuscript.

GIGA-D-24-00472
Chromosome-Level Genome Assembly of Pinus massoniana provides insights into Conifer Adaptive Evolution
Hu Chen; Xinghu Qin; Haoyu Zhang; Yinghao Chen; Yuangheng Feng; Jianhui Tan; Xinhua Chen; La Hu; Junkang Xie; jianbo xie; Zhangqi Yang
GigaScience

Dear Prof. Xie,
Your manuscript "Chromosome-Level Genome Assembly of Pinus massoniana provides insights into Conifer Adaptive Evolution" (GIGA-D-24-00472) has been assessed by our reviewers. Based on these reports, and my own assessment as Editor, I am pleased to inform you that it is potentially acceptable for publication in GigaScience, once you have carried out some essential revisions suggested by our reviewers.

Their reports, together with any other comments, are below. Please also take a moment to check our website at https://www.editorialmanager.com/giga/ for any additional comments that were saved as attachments.

Once you have made the necessary corrections, please submit a revised manuscript online at:

https://www.editorialmanager.com/giga/

If you have forgotten your username or password please use the "Send Login Details" link to get your login information. For security reasons, your password will be reset.

Please include a point-by-point within the 'Response to Reviewers' box in the submission system. Please ensure you describe additional experiments that were carried out and include a detailed rebuttal of any criticisms or requested revisions that you disagreed with. Please also ensure that your revised manuscript conforms to the journal style, which can be found in the Instructions for Authors on the journal homepage. If the data and code has been modified in the revision process please be sure to update the public versions of this too.

The due date for submitting the revised version of your article is 17 Apr 2025.

We look forward to receiving your revised manuscript soon.

Best wishes,

Qing Lan
GigaScience
www.gigasciencejournal.com

Reviewer reports:

Reviewer #1: The manuscript describes a Pinus massoniana genome sequence assembly and various aspects of genome analyses. This study provides insights for further research into the evolutionary biology of conifers and lays the groundwork for genomic-assisted breeding and sustainable management of Masson pine. Overall, this study provides the useful reference genome and potential useful SNP information. Nevertheless, I have a few suggestions to further improve the manuscript.

R: We thank Reviewer 1 for pointing out areas where the text could be clarified. In the revised manuscript, we have made the following improvements:

We have rewritten sections where the language was ambiguous, ensuring that the technical terms and arguments are presented more clearly. For instance, we have rephrased discussions on genome assembly and comparative genomics to enhance readability.
We have incorporated additional concrete examples to support our arguments. For example, we have changed specific figures that detail the genome assembly statistics and comparative analysis results, respectively. These additions provide a stronger foundation for our conclusions.

1,"As a result, we identified several key modules of CYP450 and TPS, genes associated with the biosynthesis of oleoresin, that displayed significant co-expression patterns". The authors need to be specific. Which P450s and which TPS? These are large gene families. For the P450s the very large majority of genes has no role in oleoresin biosynthesis. This part of the study needs to include knowledge of function, and should not be based solely on patterns of gene expression and association, which as pointed out above, already appears to be flawed.
R: Thank you for pointing this out. Specific CYP450 and TPS genes associated with oleoresin biosynthesis were identified and detailed in Table S11 of the supplemental materials. For instance, key CYP450 genes include CYP720B4 and CYP720B6, while critical TPS genes such as TPS1 and TPS6 were highlighted. These were identified based on co-expression patterns and SNP association studies, providing a functional basis beyond expression alone. We have updated the manuscript and add these specific genes into the manuscript (Line 581-582).

Line 70: Expand on the rationale for selecting the 6-year-old Pinus massoniana tree as the sample source.
R: The 6-year-old Pinus massoniana tree was selected for its representation of the mature growth phase when oleoresin production peaks. This stage ensures a robust dataset for investigating stress responses and metabolic activities relevant to oleoresin biosynthesis. We have added this information into the Methods Section (line 101-103).

Line 188: The enrichment analysis for stress-related gene families would be more compelling if the statistical thresholds and enrichment scores were explicitly stated in the text.
R: The enrichment analysis was performed using the clusterProfiler package with statistical thresholds set at a p-value < 0.05 and q-value < 0.05 for GO and KEGG analyses. Enrichment scores and detailed results are available in Supplemental Table S7. We have stated in the text accordingly.

Line 235: Clarify the specific methodology used for detecting horizontal gene transfer (HGT) events in the second step if there are specific criteria for identifying HGT. This will strengthen the reproducibility of the study.
R: Horizontal gene transfer (HGT) events were identified using a two-step workflow. Step one involved BLAST searches to calculate the Alien Index (AI) and outgroup percentage (outg_pct) as outlined in the methods. Step two confirmed candidate genes through phylogenetic analysis using MAFFT with 'auto' option, using trimAl with 'automated1' option, and IQ-TREE with 1,000 ultrafast bootstrapping replicates to construct ML tree. All the criteria and details have been clarified in the manuscript (line 273-290).

Line 362, Some analyses of "Gene family expansion and contraction" was overlapped with the part "Phylogenetic Analysis and Gene Family Expansion". Not sure it is in the right position, or the title need to be modified.
R: Thank you for pointing this out. We merged the paragraphs "Gene Family Expansion and Contraction" and "Phylogenetic Analysis" and the overlap has been addressed.

Line 421: Are there known interactions for AP2/ERF genes with other stress-related pathways?

R: Known interactions between AP2/ERF genes and other stress-related pathways are well-documented in literature and supported by expression data under abiotic stresses in Figure S12. These interactions are critical in modulating responses to drought and pathogen resistance, for instance, Guo LY, Wang S, Nie YQ, Shen YR, Ye XX and Wu WW. Convergent evolution of AP2/ERF III and IX subfamilies through recurrent polyploidization and tandem duplication during eudicot adaptation to paleoenvironmental changes. Plant Commun. 2022;3 6:15. doi:Artn 100420. We have also cited these references in our revised manuscript.

Line 462, "provided the experimental evidence for the direct interaction between PmMYB4 and the promoter regions of CYP450 genes": PmMYB4 should not be italicized (here it is a protein).
R: Corrected. Protein names such as PmMYB4 are no longer italicized.

Line 481-487: How many SNP were used, how many significant SNP loci were identified in the GWAS, and how many known genes were successfully annotated to the genome. Do these genes have some overlaps with these genes identified in other analyses, for example WGD or HGT, or these genes provide novel insights?
R: A total of 503,296 SNPs were analyzed, identifying 6,064 SNPs significantly associated with oleoresin synthesis. Among them, 1,056 SNPs were annotated to 10 known genes, some overlapping with WGD and HGT candidate genes, offering novel insights into oleoresin biosynthesis pathways. We have mentioned and stated this in the main text.

Line 487, Please confirm the view of "The GTP diphosphokinase CRSH protein, localized in the chloroplast, which contains EF-hand 1, EF-hand 5, HD 4, and RelA SpoT domains, is crucial for energy metabolism and could be a key regulator in the biosynthesis of oleoresin."
R: The role of the GTP diphosphokinase CRSH protein is validated through its localization in chloroplasts and its functional domains. This aligns with its potential as a key regulator in oleoresin biosynthesis. Experimental details are described in the discussion section.

Line 490, "one set of important SNPs related to oleoresin biosynthesis are the terpene synthase family": change "are" to "is"
R: Corrected. The sentence now reads, "One set of important SNPs related to oleoresin biosynthesis is the terpene synthase family."(line 559-560).

Line 522: "the transcription factor PmMYB4 was identified as a key regulatory component": "PmMYB3" should not be italicized.
R: Corrected. Italicization of protein names has been revised throughout the manuscript.
Line 604: The conclusion briefly mentions the potential applications of the findings but does not elaborate on specific genomic-assisted breeding strategies. Expanding a few words or sentences would make the discussion more actionable for applied research.
R: The conclusion now elaborates on specific applications, including the development of high-yield oleoresin genotypes and strategies to enhance stress resilience using identified SNP markers and transcription factors like PmMYB4. We have included in these in the discussion (line 687-691).

Reviewer #2: With the advancement of sequencing technologies, plant genome research has entered an era of higher precision. Recent publications on the high-quality genomes of species within the Pinus genus have provided valuable resources for researchers to further explore the development and conservation of this economically important group. This study performed high-quality genome assembly and annotation of Pinus massoniana using PacBio long-read sequencing and Hi-C data. Additionally, the analyses of Pinus massoniana's genomic features and evolution, and the mechanism of oleoresin biosynthesis offer new insights into the biology of the Pinus genus. However, regarding the discussion of the adaptation of Pinus massoniana, the current analysis lacks sufficient support. Incorporating an analysis of the correlation between the genomic data, the environmental characteristics of its distribution, and relevant adaptive traits would enhance the persuasiveness and scientific rigor of the research.

R: Thanks very much for Reviewer 2 for providing invaluable comments, which could greatly improve the quality of our manuscript. We have read and revised the manuscript according to the reviewer’s comments.

Below, we reply to the reviewer's comments point-by-point.

I have a few minor suggestions：
Lines 28-29: Understanding the genetic underpinnings of P. massoniana's adaptation to diverse environments is of paramount importance, particularly concerning stress responses and oleoresin biosynthesis. The logic is too abrupt; add causal conjunctions to link it with the preceding discussion smoothly.
Response: Thank you for the suggestion. We have revised the sentence to enhance clarity and flow (line 27-30).

Lines 32-33: However, existing studies predominantly focus on a limited array of conifer species such as Picea abies, Pinus taeda, and Picea glauca. What are the limitations of these studies? What advantages does P. massoniana in this study have in comparison?
Response: The limitations of prior studies and the advantages of P. massoniana are discussed following that sentence in Introduction. We have expanded on it in the revised manuscript (line 36-47).

Lines 51-52: In this context, the sequencing of the P. massoniana genome heralds a new era in comprehending conifer biology. Why? The reasoning is missing.
R: We have added the reasoning for sequencing P. massoniana's genome:

Revised Text: "The sequencing of the Pinus massoniana genome represents a significant advancement in conifer biology. This chromosomal-level assembly provides crucial insights into the molecular mechanisms driving both resin biosynthesis and defense responses within the Pinus genus. By understanding the genetic pathways behind resin production and how conifers defend against pathogens and environmental stress, this research opens new avenues for improving conifer resilience and optimizing the production of resin-based compounds." (line 76-81).

Lines 314-315: Figure 1A-B
Regarding Figure 1B, if there are no methodological innovations, it can be moved to supplementary figures. Consider placing genome quality assessment results instead.
R: We have moved Figure 1B to supplementary Figures and replaced the genome quality assessment results (see Figure 1).
Line 339: Figure 2C
The arrangement of Figure 2 should correspond to the order of results described in the text.
R: We have reorganized the narrative structure of the results to align with the figure (line 362-367; line 382-406; line 412-433). Figure 2C is in line 402, 470.

Lines 359-361: This period of gene family dynamics coincided with critical adaptations in plant evolution, particularly in response to environmental changes.
The evidence is insufficient. Please supplement the analysis with results correlating adaptive traits related to environmental changes and gene family dynamics.
R：We have re-organized the structure of the paragraph. Thus, this statement has been eliminated (line 423-425).
In terms of gene family related to adaptations, we have added specific sentences to environmental adaptations, such as stress-related gene families (e.g., CYP450, TPS, AP2/ERF) that are linked to the species' resilience in challenging environments. These genes correlate with adaptive traits like pathogen resistance, drought tolerance, and oleoresin production (line 394-396; line 486-488; line 495-496).

Lines 364-366: Our results showed that 1,356 gene families have expanded in the P. massoniana lineage, while 982 gene families have contracted (Figure 3A).
There is a mismatch between the figure and the text.
R: We have corrected the mismatch by updating the figure and text to reflect the correct number of expanded and contracted gene families. The revised text now accurately describes the findings:
Revised Text: "Our analysis revealed 6,434 gene families expanded, while 2,405 gene families contracted (Figure 2A)." (line 392-393).

Lines 378-380: The increased capacity for flavonoid production likely contributes to the species' resilience

in diverse and challenging environments.
The results do not show this; avoid speculative discussions in the results section.
R: We have revised the speculative text. The suggestion regarding flavonoid production has been removed from the results section (line 444).

Line 530: Discussion
Please cite specific results as evidence in the discussion section (e.g., Figure X, Table X, etc.).
R: We have updated the discussion section to include specific references to figures and tables where applicable.
Lines 539-540: ...... underscoring the evolutionary strategies that enable P. massoniana to thrive in diverse habitats across Southern China.
It would be beneficial to include supplementary data on habitat distribution and compare it with other groups' habitats.
R: Thank you for your insightful suggestion regarding the inclusion of supplementary data on habitat distribution and comparative analysis with other groups' habitats. We acknowledge the value such information would add to our study. Our research primarily focuses on the genomic analysis of P. massoniana, and provides the first- chromosome reference genome for future genetic studies, and the results from population structure, genes under selection, expended and duplicated genes provide insights for the adaptation of P. massoniana. Compiling comprehensive habitat distribution data and conducting comparative habitat analyses could add information on the evolutionary strategies of P. massoniana, however, this also would distract the main focus of our study.
We have changed our statement ("deleted a diverse habitat"). We appreciate your understanding and hope that the genomic insights presented in our study will serve as a foundation for future research exploring more evolutionary aspects of P. massoniana and related species.

Line 578: The population structure analysis......
Consider presenting the related results in the main text of the article.
R: These related results present in the "Genetic basis of Oleoresin Yield", now we have removed this discussion and changed the related result section to "The Population Genetic Structure and the Genetic Basis of Oleoresin Yield" (line 535, line 648-657).

Line 595: ......possibly linked to significant climatic changes during the Miocene epoch.
Can you add relevant climate model change curves?

R: We appreciate your suggestion to include climate model change curves related to the Miocene epoch. We tried hard to make such figures including climate changes (curves) during revision, however, the data we inquired from elsewhere differed due to different model selection. While such data would indeed enrich the discussion, the current scope of our study is focused on the genomic aspects of Pinus massoniana, and integrating detailed paleoclimatic models would require extensive additional research and analysis beyond our present objectives. However, we acknowledge the importance of this perspective and suggest that future studies could explore the relationship between historical climate changes and the evolutionary adaptations of P. massoniana in greater detail. The latest manuscript has cited researches of climate characteristics in Miocene, which could align the context of our findings. (line 663-666).
References:
1. Zachos J, Pagani M, Sloan L, Thomas E and Billups K. Trends, rhythms, and aberrations in global climate 65 Ma to present. Science. 2001;292 5517:686-93. doi:10.1126/science.1059412.
2. Reuter M, Kern A K, Harzhauser M, et al. Global warming and South Indian monsoon rainfall-lessons from the Mid-Miocene[J]. Gondwana Research, 2013, 23(3): 1172-1177.

Lines 599-601: ......supports the notion that genomic redundancy can act as a reservoir for evolutionary innovation, especially in response to biotic and abiotic stressors.
Add relevant references.
R: We have added references to relevant literature supporting the idea that genomic redundancy can foster evolutionary innovation in response to environmental stressors (line 672).
References:
1. Almeida-Silva F and Van de Peer Y. Whole-genome Duplications and the Long-term Evolution of Gene Regulatory Networks in Angiosperms. Molecular biology and evolution. 2023;40 7

doi:10.1093/molbev/msad141.
2. Panchy N, Lehti-Shiu M and Shiu SH. Evolution of Gene Duplication in Plants. Plant Physiol. 2016;171 4:2294-316. doi:10.1104/pp.16.00523.

Lines 618-619: ......P. massoniana has benefited from genetic material that enhances its adaptability to environmental stresses.
All organisms face some degree of environmental stress; specify the types of environmental stresses of P. massoniana.
R: We have specified the types of environmental stresses to which P. massoniana is adapted, including drought, pest attacks, and pathogens (line 696).

Line 637: ......emphasizing its adaptive strategies.....
Consider briefly discussing these adaptive strategies in the background section.
R: We have introduced a brief discussion on adaptive strategies, including drought resistance and oleoresin biosynthesis, in the background section to provide context for the genomic analyses (line 62-68) .

We are grateful for the reviewers' and editor's constructive feedback, which has significantly improved the manuscript. We believe the revisions address all the concerns raised and enhance the clarity, completeness, and scientific rigor of the study. We are confident that the revised manuscript meets the journal's standards and is now suitable for publication.

Should further revisions be required, we are more than willing to make additional adjustments.

Thank you again for your guidance and support.

Sincerely,

Prof Jianbo Xie

Editorial Manager®

Cookie Preference Center

We use cookies which are necessary to make our site work. We may also use additional cookies to analyze, improve and personalize our content and your digital experience. For more information, see our Cookie Policy.

You may choose not to allow some types of cookies. However, blocking some types may impact your experience of our site and the services we are able to offer. See the different category headings below to find out more or change your settings.

Allow all

Manage Consent Preferences

Strictly Necessary Cookies

Always active

These cookies are necessary for the website to function and cannot be switched off in our systems. They are usually only set in response to actions made by you which amount to a request for services, such as setting your privacy preferences, logging in or filling in forms. You can set your browser to block or alert you about these cookies, but some parts of the site will not then work. These cookies do not store any personally identifiable information.

Functional Cookies

These cookies enable the website to provide enhanced functionality and personalisation. They may be set by us or by third party providers whose services we have added to our pages. If you do not allow these cookies then some or all of these services may not function properly.

Performance Cookies

These cookies allow us to count visits and traffic sources so we can measure and improve the performance of our site. They help us to know which pages are the most and least popular and see how visitors move around the site.

Targeting Cookies

These cookies may be set through our site by our advertising partners. They may be used by those companies to build a profile of your interests and show you relevant adverts on other sites. If you do not allow these cookies, you will experience less targeted advertising.

Cookie List

Clear

Apply Cancel

Consent Leg.Interest

Confirm my choices

Powered by onetrust
