# Supplementary material for: Chromosome-level genome assembly of Pinus massoniana provides insights into conifer adaptive evolution
Source: Gigascience. 2025 May 30;14:giaf056. doi: 10.1093/gigascience/giaf056 (PMC12122422; doi:10.1093/gigascience/giaf056)
Supplement: giaf056_Reviewer_1_Report_original_submission — Nian Wang -- 1/15/2025 [file giaf056_reviewer_1_report_original_submission.pdf]

Reviewer Report

Title: Chromosome-Level Genome Assembly of Pinus massoniana provides insights into Conifer Adaptive Evolution

Version: Original Submission **Date:** 1/15/2025

Reviewer name: Nian Wang

Reviewer Comments to Author:

The manuscript describes a Pinus massoniana genome sequence assembly and various aspects of genome analyses. This study provides insights for further research into the evolutionary biology of conifers and lays the groundwork for genomic-assisted breeding and sustainable management of Masson pine. Overall, this study provides the useful reference genome and potential useful SNP information. Nevertheless, I have a few suggestions to further improve the manuscript.
1,"As a result, we identified several key modules of CYP450 and TPS, genes associated with the biosynthesis of oleoresin, that displayed significant co-expression patterns". The authors need to be specific. Which P450s and which TPS? These are large gene families. For the P450s the very large majority of genes has no role in oleoresin biosynthesis. This part of the study needs to include knowledge of function, and should not be based solely on patterns of gene expression and association, which as pointed out above, already appears to be flawed.
Line 70: Expand on the rationale for selecting the 6-year-old Pinus massoniana tree as the sample source.
Line 188: The enrichment analysis for stress-related gene families would be more compelling if the statistical thresholds and enrichment scores were explicitly stated in the text.
Line 235: Clarify the specific methodology used for detecting horizontal gene transfer (HGT) events in the second step if there are specific criteria for identifying HGT. This will strengthen the reproducibility of the study.
Line 362, Some analyses of "Gene family expansion and contraction" was overlapped with the part "Phylogenetic Analysis and Gene Family Expansion". Not sure it is in the right position, or the title need to be modified.
Line 421: Are there known interactions for AP2/ERF genes with other stress-related pathways?
Line 462, "provided the experimental evidence for the direct interaction between PmMYB4 and the promoter regions of CYP450 genes": PmMYB4 should not be italicized (here it is a protein).
Line 481-487: How many SNP were used, how many significant SNP loci were identified in the GWAS, and how many known genes were successfully annotated to the genome. Do these genes have some overlaps with these genes identified in other analyses, for example WGD or HGT, or these genes provide novel insights?
Line 487, Please confirm the view of "The GTP diphosphokinase CRSH protein, localized in the chloroplast, which contains EF-hand 1, EF-hand 5, HD 4, and RelA SpoT domains, is crucial for energy metabolism and could be a key regulator in the biosynthesis of oleoresin."
Line 490, "one set of important SNPs related to oleoresin biosynthesis are the terpene synthase family":

change "are" to "is"
Line 522: "the transcription factor PmMYB4 was identified as a key regulatory component": "PmMYB3" should not be italicized.
Line 604: The conclusion briefly mentions the potential applications of the findings but does not elaborate on specific genomic-assisted breeding strategies. Expanding a few words or sentences would make the discussion more actionable for applied research.

Methods

Are the methods appropriate to the aims of the study, are they well described, and are necessary controls included? Choose an item.

Conclusions

Are the conclusions adequately supported by the data shown? Choose an item.

Reporting Standards

Does the manuscript adhere to the journal's guidelines on minimum standards of reporting? Choose an item.

Choose an item.

Statistics

Are you able to assess all statistics in the manuscript, including the appropriateness of statistical tests used? Choose an item.

Quality of Written English

Please indicate the quality of language in the manuscript: Choose an item.

Declaration of Competing Interests

Please complete a declaration of competing interests, considering the following questions:

- Have you in the past five years received reimbursements, fees, funding, or salary from an organisation that may in any way gain or lose financially from the publication of this manuscript, either now or in the future?
- Do you hold any stocks or shares in an organisation that may in any way gain or lose financially from the publication of this manuscript, either now or in the future?
- Do you hold or are you currently applying for any patents relating to the content of the manuscript?

- Have you received reimbursements, fees, funding, or salary from an organization that holds or has applied for patents relating to the content of the manuscript?
- Do you have any other financial competing interests?
- Do you have any non-financial competing interests in relation to this paper?

If you can answer no to all of the above, write 'I declare that I have no competing interests' below. If your reply is yes to any, please give details below.

I declare that I have no competing interests

I agree to the open peer review policy of the journal. I understand that my name will be included on my report to the authors and, if the manuscript is accepted for publication, my named report including any attachments I upload will be posted on the website along with the authors' responses. I agree for my report to be made available under an Open Access Creative Commons CC-BY license (http://creativecommons.org/licenses/by/4.0/). I understand that any comments which I do not wish to be included in my named report can be included as confidential comments to the editors, which will not be published.

Choose an item.

To further support our reviewers, we have joined with Publons, where you can gain additional credit to further highlight your hard work (see: https://publons.com/journal/530/gigascience). On publication of this paper, your review will be automatically added to Publons, you can then choose whether or not to claim your Publons credit. I understand this statement.

Yes Choose an item.
