# Supplementary material for: Chromosome-level genome assembly of Pinus massoniana provides insights into conifer adaptive evolution
Source: Gigascience. 2025 May 30;14:giaf056. doi: 10.1093/gigascience/giaf056 (PMC12122422; doi:10.1093/gigascience/giaf056)
Supplement: giaf056_Reviewer_2_Report_original_submission — Yang Dong, Ph.D -- 1/16/2025 [file giaf056_reviewer_2_report_original_submission.pdf]

Reviewer Report

Title: Chromosome-Level Genome Assembly of Pinus massoniana provides insights into Conifer Adaptive Evolution

Version: Original Submission **Date:** 1/16/2025

Reviewer name: Yang Dong, Ph.D

Reviewer Comments to Author:

With the advancement of sequencing technologies, plant genome research has entered an era of higher precision. Recent publications on the high-quality genomes of species within the Pinus genus have provided valuable resources for researchers to further explore the development and conservation of this economically important group. This study performed high-quality genome assembly and annotation of Pinus massoniana using PacBio long-read sequencing and Hi-C data. Additionally, the analyses of Pinus massoniana's genomic features and evolution, and the mechanism of oleoresin biosynthesis offer new insights into the biology of the Pinus genus. However, regarding the discussion of the adaptation of Pinus massoniana, the current analysis lacks sufficient support. Incorporating an analysis of the correlation between the genomic data, the environmental characteristics of its distribution, and relevant adaptive traits would enhance the persuasiveness and scientific rigor of the research.
I have a few minor suggestionsï¼š
Lines 28-29: Understanding the genetic underpinnings of P. massoniana's adaptation to diverse environments is of paramount importance, particularly concerning stress responses and oleoresin biosynthesis.
The logic is too abrupt; add causal conjunctions to link it with the preceding discussion smoothly.
Lines 32-33: However, existing studies predominantly focus on a limited array of conifer species such as Picea abies, Pinus taeda, and Picea glauca.
What are the limitations of these studies? What advantages does P. massoniana in this study have in comparison?
Lines 51-52: In this context, the sequencing of the P. massoniana genome heralds a new era in comprehending conifer biology.
Why? The reasoning is missing.
Lines 314-315: Figure 1A-B
Regarding Figure 1B, if there are no methodological innovations, it can be moved to supplementary figures. Consider placing genome quality assessment results instead.
Line 339: Figure 2C
The arrangement of Figure 2 should correspond to the order of results described in the text.
Lines 359-361: This period of gene family dynamics coincided with critical adaptations in plant evolution, particularly in response to environmental changes.
The evidence is insufficient. Please supplement the analysis with results correlating adaptive traits related to environmental changes and gene family dynamics.
Lines 364-366: Our results showed that 1,356 gene families have expanded in the P. massoniana lineage,

while 982 gene families have contracted (Figure 3A).
There is a mismatch between the figure and the text.
Lines 378-380: The increased capacity for flavonoid production likely contributes to the species' resilience in diverse and challenging environments.
The results do not show this; avoid speculative discussions in the results section.
Line 530: Discussion
Please cite specific results as evidence in the discussion section (e.g., Figure X, Table X, etc.).
Lines 539-540: ...... underscoring the evolutionary strategies that enable P. massoniana to thrive in diverse habitats across Southern China.
It would be beneficial to include supplementary data on habitat distribution and compare it with other groups' habitats.
Line 578: The population structure analysis......
Consider presenting the related results in the main text of the article.
Line 595: ......possibly linked to significant climatic changes during the Miocene epoch.
Can you add relevant climate model change curves?
Lines 599-601: ......supports the notion that genomic redundancy can act as a reservoir for evolutionary innovation, especially in response to biotic and abiotic stressors.
Add relevant references.
Lines 618-619: ......P. massoniana has benefited from genetic material that enhances its adaptability to environmental stresses.
All organisms face some degree of environmental stress; specify the types of environmental stresses of P. massoniana.
Line 637: ......emphasizing its adaptive strategies.....
Consider briefly discussing these adaptive strategies in the background section.

Methods

Are the methods appropriate to the aims of the study, are they well described, and are necessary controls included? Choose an item.

Conclusions

Are the conclusions adequately supported by the data shown? Choose an item.

Reporting Standards

Does the manuscript adhere to the journal's guidelines on [minimum standards of reporting?](#) Choose an item.

Choose an item.

Statistics

Are you able to assess all statistics in the manuscript, including the appropriateness of statistical tests used? Choose an item.

Quality of Written English

Please indicate the quality of language in the manuscript: Choose an item.

Declaration of Competing Interests

Please complete a declaration of competing interests, considering the following questions:

- Have you in the past five years received reimbursements, fees, funding, or salary from an organisation that may in any way gain or lose financially from the publication of this manuscript, either now or in the future?
- Do you hold any stocks or shares in an organisation that may in any way gain or lose financially from the publication of this manuscript, either now or in the future?
- Do you hold or are you currently applying for any patents relating to the content of the manuscript?
- Have you received reimbursements, fees, funding, or salary from an organization that holds or has applied for patents relating to the content of the manuscript?
- Do you have any other financial competing interests?
- Do you have any non-financial competing interests in relation to this paper?

If you can answer no to all of the above, write 'I declare that I have no competing interests' below. If your reply is yes to any, please give details below.

I declare that I have no competing interests' below.

I agree to the open peer review policy of the journal. I understand that my name will be included on my report to the authors and, if the manuscript is accepted for publication, my named report including any attachments I upload will be posted on the website along with the authors' responses. I agree for my report to be made available under an Open Access Creative Commons CC-BY license (http://creativecommons.org/licenses/by/4.0/). I understand that any comments which I do not wish to be included in my named report can be included as confidential comments to the editors, which will not be published.

Choose an item.

To further support our reviewers, we have joined with Publons, where you can gain additional credit to further highlight your hard work (see: https://publons.com/journal/530/gigascience). On publication of this paper, your review will be automatically added to Publons, you can then choose whether or not to claim your Publons credit. I understand this statement.

Yes Choose an item.
